# Inland Waters Increasingly Produce and Emit Nitrous Oxide

**DOI:** 10.1021/acs.est.3c04230

**Published:** 2023-08-30

**Authors:** Junjie Wang, Lauriane Vilmin, José M. Mogollón, Arthur H. W. Beusen, Wim J. van Hoek, Xiaochen Liu, Philip A. Pika, Jack J. Middelburg, Alexander F. Bouwman

**Affiliations:** † Department of Earth Sciences, Utrecht University, Princetonlaan 8a, 3584 CB Utrecht, The Netherlands; ‡ Deltares, P.O. Box 177, 2600 MH Delft, The Netherlands; § Department of Industrial Ecology, Leiden University, 2300 RA Leiden, The Netherlands; ∥ PBL Netherlands Environmental Assessment Agency, P.O. Box 30314, 2500 GH The Hague, The Netherlands; ⊥ Faculty of Science, Earth and Climate, Free University of Amsterdam, de Boelelaan 1105, 1081 HV Amsterdam, The Netherlands

**Keywords:** nitrous oxide, greenhouse gas emission, inland waters, N_2_O cycling, long-term temporal changes, spatial distributions, closed N_2_O budget, integrated process-based modeling

## Abstract

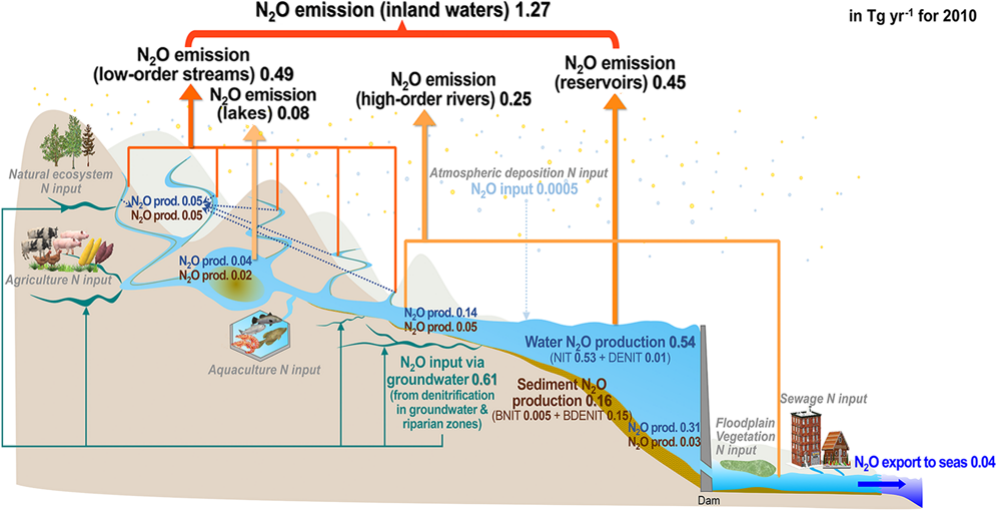

Nitrous oxide (N_2_O) is a long-lived greenhouse gas and currently contributes ∼10% to global greenhouse warming. Studies have suggested that inland waters are a large and growing global N_2_O source, but whether, how, where, when, and why inland-water N_2_O emissions changed in the Anthropocene remains unclear. Here, we quantify global N_2_O formation, transport, and emission along the aquatic continuum and their changes using a spatially explicit, mechanistic, coupled biogeochemistry–hydrology model. The global inland-water N_2_O emission increased from 0.4 to 1.3 Tg N yr^–1^ during 1900–2010 due to (1) growing N_2_O inputs mainly from groundwater and (2) increased inland-water N_2_O production, largely in reservoirs. Inland waters currently contribute 7 (5–10)% to global total N_2_O emissions. The highest inland-water N_2_O emissions are typically in and downstream of reservoirs and areas with high population density and intensive agricultural activities in eastern and southern Asia, southeastern North America, and Europe. The expected continuing excessive use of nutrients, dam construction, and development of suboxic conditions in aging reservoirs imply persisting high inland-water N_2_O emissions.

## Abstract 2

For the first time, this paper presents the methodologically consistent inland-water nitrous oxide budget and their spatiotemporal variations during 1900−2010.

## 1 Introduction

The Earth’s climate is warming at an unprecedented rate, mainly because of increasing greenhouse gas concentrations. Atmospheric nitrous oxide (N_2_O) concentrations have increased from 270 to 333 ppb since 1750^1,2^ (Figure S1 in the Supporting Information, SI), and N_2_O currently contributes ∼10% to the global anthropogenic greenhouse warming effect.^1,3^ N_2_O production is controlled by nitrogen (N) transformation processes in soils and aquatic environments, including denitrification (reduction of nitrite, NO_2_^–^, and nitrate, NO_3_^–^)^4,5^ and nitrification (oxidation of ammonium, NH_4_^+^),^5,6^ and can escape to the atmosphere. These N_2_O-related biogeochemical processes are influenced by multiple environmental factors including temperature, oxygen conditions, acidity, hydrology, concentrations of reactive N forms (resulting from various natural and anthropogenic sources), availability of organic matter, and sediment accumulation.^7−9^ Due to the rapidly growing population and production of food and energy since the beginning of the industrial era, the global N cycle has accelerated by increasing anthropogenic N inputs from agriculture, wastewater, aquaculture, and atmospheric deposition.^10−14^ Between 1900 and 2000, N inputs to global surface waters have almost doubled.^10^ The increasing reactive N availability as substrates for nitrification and denitrification almost inevitably leads to more N_2_O production. Surface waters in globally expanding agricultural areas, particularly low-order streams, are typically N_2_O-supersaturated compared to the atmosphere.^15−21^ As a result, riverine N_2_O emissions have increased rapidly.^22−28^ Reservoirs created by damming rivers are important sources of atmospheric N_2_O,^29−37^ and reservoir N_2_O emissions are growing due to the rapid increase in the global reservoir volume. Another process causing increasing N_2_O emissions from aging reservoirs is the accumulation of organic material and subsequent development of low-oxygen conditions, which may trigger N_2_O production through incomplete denitrification and incomplete nitrification.^37^ In the scope of current efforts to mitigate human emissions and warming, it is therefore essential to assess the contribution of inland waters (i.e., streams, rivers, lakes, and reservoirs) to the global N_2_O budget.

The Intergovernmental Panel on Climate Change (IPCC) guidelines proposed the use of an emission factor (i.e., N_2_O emission is calculated as a fraction of N inputs into the soil-hydrological system). This approach yields a N_2_O-emission estimate of 0.6 (0.1–2.9) Tg N yr^–1^ from global rivers and coastal areas in the 2000s, constituting ∼10% of the global anthropogenic emission of 6.9 Tg N yr^–1^^38^ Although transparent and simple, the IPCC (AR5) approach ignores the spatial heterogeneity and temporal variability of hydrological conditions (construction of dams and reservoirs), N delivery to inland waters due to changes in human activities (land use, fertilizer use, intensification of agriculture, population, wastewater discharge, etc.), and inland-water biogeochemical processes. Alternatively, empirical relations between N_2_O and N are used for estimating N_2_O emissions from individual rivers^17,22,24,39−45^ or specific waterbody types^18,30,32,34−36,46−53^ and to obtain snapshot global-scale N_2_O-emission estimates.^23,26,27,54−61^ These estimates can include various uncertainties due to scant or biased sampling, limited validity of correlations applied, and lumping of data and processes to aggregated levels with temporal and/or spatial variability ignored. Moreover, the recently reported sinks of N_2_O in some aquatic systems complexify the question of inland-water N_2_O fluxes and their spatiotemporal changes and urge an in-depth understanding of the mechanism behind quantities.^62,63^ Process-based models have been developed to quantify N_2_O fluxes from some inland waterbodies including those in the latest IPCC AR6.^7,25,64−66^ However, so far, nonuniform approaches are used to estimate N_2_O emissions from different waterbodies, which are added up to estimate the total inland-water N_2_O emissions.^7,67^ The spatiotemporal changes in global total inland-water N_2_O emissions in the Anthropocene and the underlying mechanisms remain unclear due to the lack of a unified methodology to track various N sources, cover all waterbodies, and resolve key inland-water N processes and their coupling with regulating environmental factors (temperature, hydrology, availability of oxygen and organic matter, and sediments). A noteworthy limitation of existing approaches is that they do not consider the increasing reservoir volume and evolving biogeochemical conditions in aging reservoirs and the delivery of dissolved N_2_O from groundwater to inland waters, a source that was first recognized in the 1980s.^68−70^

In this study, we quantify the spatiotemporal changes in the global freshwater N_2_O budget (input, production, consumption, emission to the atmosphere, and export to coastal waters) during 1900–2010. Specifically, we quantify the role of rivers, streams, lakes, reservoirs, and groundwater in N_2_O budgets regionally and globally as a function of the global acceleration of the N cycle and the increasing number of reservoirs and their aging process. Our analysis uses the Integrated Model to Assess the Global Environment (IMAGE)-Dynamic Global Nutrient Model (IMAGE-DGNM),^71^ a spatially explicit, coupled, process-based description of N cycle in the terrestrial-groundwater-surface water system. This model resolves N_2_O dynamics in surface freshwaters, which quantitatively considers water temperature, oxygen conditions, hydrology, loadings of reactive N forms (from various natural and anthropogenic sources), availability of organic matter, and sediment accumulation.

## 2 Materials and Methods

### 2.1 Existing Implementation of N Cycling in IMAGE-DGNM

**Figure 1:**
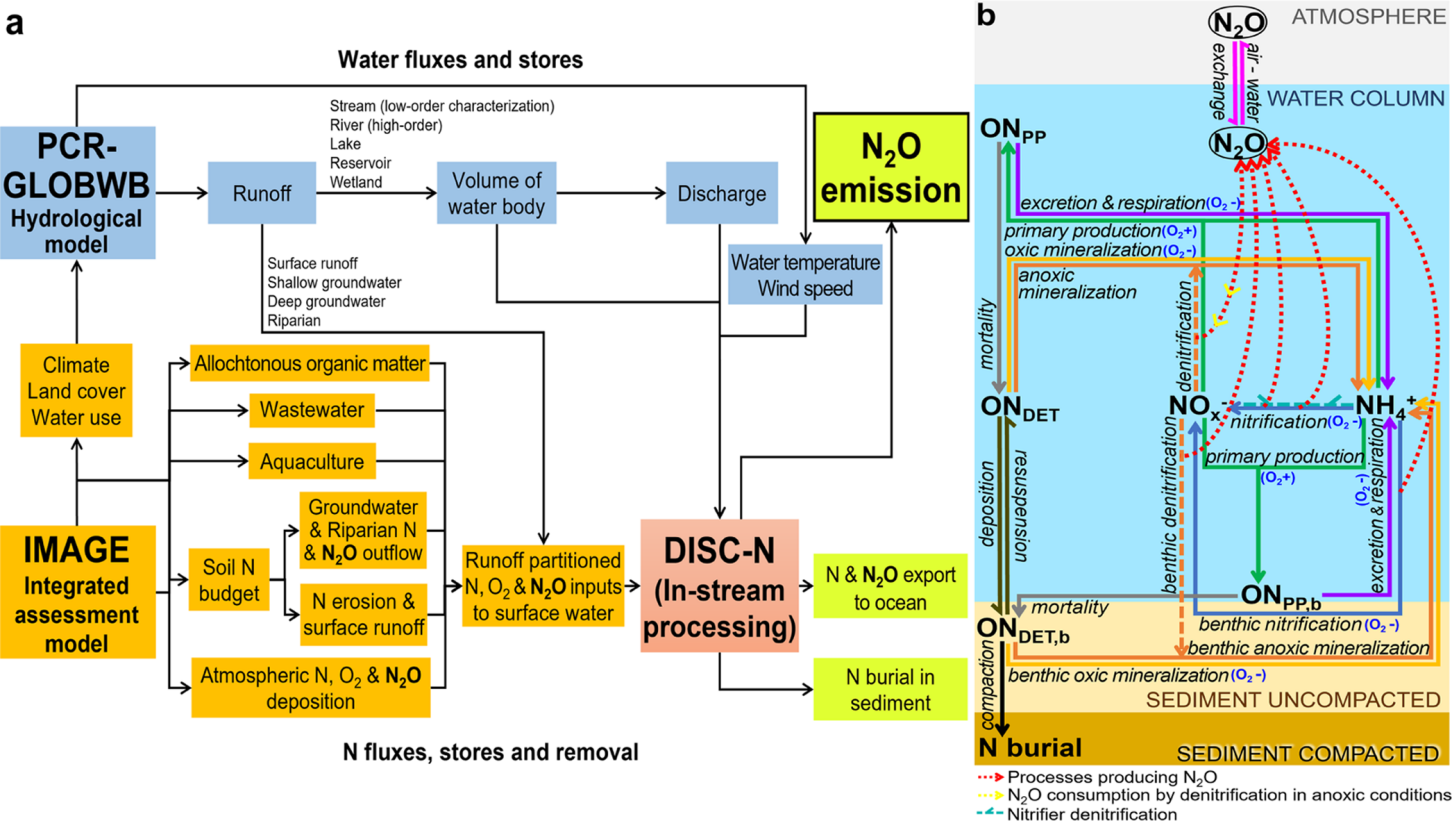
(a) Scheme of N flows and processes (including N_2_O dynamics) in the IMAGE-DGNM model and (b) scheme of N_2_O dynamics in the DISC-NITROGEN module of IMAGE-DGNM at a 0.5 × 0.5° grid scale. Water fluxes in panel (a) link various landscape components: soils, aquifers, riparian zones, streams, rivers, wetlands, floodplains, lakes, and reservoirs. For descriptions of the equations, stoichiometry, and parameters for the inland-water N processes of DISC-NITROGEN shown in panel (b), see the full model description in ref (71).

The Integrated Model to Assess the Global Environment (IMAGE)-Dynamic Global Nutrient Model (IMAGE-DGNM) is a global spatially explicit, integrated modeling framework to simulate dynamic coupled biogeochemical cycling of multiple nutrient and carbon forms from terrestrial systems to surface freshwaters.^71−74^ Within the IMAGE-DGNM framework, the IMAGE model^75^ provides long-term land cover, climate, and water use data to the hydrology model PCR-GLOBWB,^76^ and their coupling provides nutrient delivery fluxes to the process-based DISC module^71−73^ (Figure 1 and Table S2a). The hydrological model PCR-GLOBWB dynamically simulates the water area, volume, runoff, and discharge in the different waterbodies including high-order (Strahler orders ≥6) rivers, lakes, and reservoirs for each year,^76,77^ with characteristics of drainage network, lakes, and reservoirs from refs (78−80), respectively. The hydrological characteristics of low-order (Strahler orders < 6) streams for each 0.5-by-0.5-degree grid are estimated based on the hydrology of high-order rivers using the parameterization method proposed by Wollheim et al.,^81^ with detailed descriptions of equations and parameters used in IMAGE-DGNM in refs (71, 82). Extensive validation in open-access publications has shown that the runoff, water discharge, total water storage, evapotranspiration, precipitation, and other hydrological properties simulated by PCR-GLOBWB (hydrological module in IMAGE-DGNM) agree well with observations.^76,77,83^ In this study, the lake area is from Global Lakes and Wetlands Database (GLWD1),^79^ the reservoir area is from the Global Reservoir and Dam database (GRanD v1.3)^80^ and introduced dynamically based on the construction year, and IMAGE-DGNM estimates that low-order streams account for 61–66% of the total inland-water area during 1900–2010, which is very close to the 69% estimated using data from HydroSHEDS^84^ by Raymond et al.^85^ Therefore, surface water area, a factor proven important for estimating inland-water gas emissions,^85,86^ is properly simulated in IMAGE-DGNM. In this study, a 1 year temporal resolution is used in all modules within IMAGE-DGNM to explore the long-term change in the inland-water N_2_O budget on the global scale.

The implementation of N cycling in the DISC module (DISC-NITROGEN)^71^ describes the inland-water dynamics of ammonium (NH_4_^+^), nitrate + nitrite (NO*_x_*^–^, = NO_2_^–^ + NO_3_^–^), and organic N (ON). ON is composed of detrital ON (ON_DET_) and ON constituting living primary producers (ON_PP_) in the water column as well as ON_DET_ and ON_PP_ accumulated in the benthic layer (i.e., ON_DET,b_ and ON_PP,b_). The inputs of these different N forms to global surface freshwaters for the period 1900–2010 are based on Vilmin et al.,^87^ including those from agricultural and natural ecosystems via surface runoff and groundwater discharge, wastewater, vegetation in flooded areas, atmospheric deposition, and aquaculture (Figure S2). Dynamics of the suspended material and sediments and dissolved oxygen (DO) are explicitly represented, and the accumulation of ON in sediments and consequent oxygen consumption are tracked,^71,88^ since they impact N cycling and N_2_O-related processes under suboxic conditions (Figure 1). Equations and parameters used to describe relevant processes of N transformations, exchange, and transport with the spatiotemporal changes in hydrological, temperature, and oxic conditions, sediment dynamics, and N loading from human and natural sources, are available in ref (71). Process rates and resulting amounts of different biogeochemical species are assessed per year for each 0.5 × 0.5° grid, for every waterbody type (i.e., stream orders from 1 to ≥6, lakes and reservoirs) in global inland waters.

### 2.2 Riverine N_2_O Module: Modeling of N_2_O Cycling and Emissions from Global Watersheds

In the present work, we extend DISC-NITROGEN with the processes affecting N_2_O dynamics. This requires descriptions of external N_2_O inputs to surface freshwaters as well as inland-water processes in water and sediments and the exchange at the water–atmosphere interface.

#### 2.2.1 Estimation of N_2_O Inputs to Inland Waters

##### 2.2.1.1 N_2_O Input from Groundwater

Groundwaters in both agricultural and forest areas are often N_2_O-supersaturated relative to the atmosphere, with reported concentrations of up to 4000 μg of N_2_O-N/L.^68,69,89−97^ The IMAGE-DGNM approaches for simulating N flows across landscapes and waterscapes (Figure S4) are extensively described in a series of papers.^10,82,98^ Briefly, soil denitrification and leaching to groundwater depend on soil wetness and temperature and a number of physical and chemical soil properties (land use, soil texture, aeration, content of soil organic carbon) relying on simplifications of a range of other published models. Groundwater N transport, denitrification, and travel time are modeled on the basis of the porosity of the parent material and the half-life of NO_3_^–^ (derived from the literature), which is related to lithology. Two groundwater compartments are implemented^82^ (Figure S4): (i) a 50 m thick deep groundwater layer (with high water residence time) that is located below the shallow groundwater system and directly flows to the rivers and streams at greater distances (>1 km) and (ii) a shallow groundwater system (top 5 m of saturated zones where water is retained over short residence times) that can infiltrate into the deep groundwater system or enter surface waterbodies at short distances (<1 m) by either moving through the riparian zone to streams and rivers or bypassing the riparian zone and flowing directly to surface waterbodies (rivers, streams, lakes, and reservoirs). Riparian denitrification depends on the characteristics of the groundwater flow, soil, and climate.^82,99^

We assume that N_2_O discharged from groundwater to surface water (L_N_2_O_grw_) stems from two parts of net N_2_O production: (i) shallow groundwater denitrification (L_N_2_O_grw_sgrw_) and (ii) riparian denitrification (L_N_2_O_grw_rip_), and the N_2_O is transported by groundwater to surface waters where emission or further transport can occur. IMAGE-DGNM considers N_2_O produced in soil profiles as direct emission to the atmosphere;^82,99^ thus, it is assumed not transported by groundwater to surface waters. In the model, the N_2_O possibly produced via nitrification in groundwater^92^ is assumed to be consumed during transport, thus not reaching surface waters. Based on refs (58, 94−96, 100−111) and Figure S5, a constant fraction (*f*_N_2_O_sgrw_ = 1%, median of the 278 reported measurement-based values; see Text S1 for details) of shallow groundwater denitrification (DENIT_sgrw_)^82,99^ is calculated as the associated net N_2_O production (i.e., L_N_2_O_grw_sgrw_, part i), which is transported to surface waters directly or indirectly via deep groundwater or riparian zones (Figure S4), following eq 1
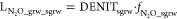
1

The net N_2_O production via riparian denitrification that is transported to surface waters (i.e., part ii) is calculated from the local denitrification and soil pH in each grid using eq 2 ^82,99^
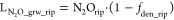
2where N_2_O_rip_ is the total net N_2_O production in the riparian zone of the grid estimated by IMAGE-DGNM^82,99^ and *f*_den_rip_ is the N removal rate (i.e., denitrification rate) used to further partition the two N_2_O pathways in riparian zones: N_2_O_rip_·*f*_den_rip_ is assumed as the direct N_2_O emission to the atmosphere in terrestrial areas, and N_2_O_rip_·(1 – *f*_den_rip_) is assumed as the N_2_O transported by shallow groundwater to surface waters.

##### 2.2.1.2 N_2_O Input from Atmospheric Deposition

We assume that the concentration of dissolved N_2_O in rainfalls equals the atmospheric equilibrium N_2_O concentration (*C*_N_2_O,sat_, in mol·m^–3^); the N_2_O inputs from atmospheric deposition to surface water (L_N_2_O_atm_) are thus calculated as precipitation (in m yr^–1^) × surface water area (in m^2^) × *C*_N_2_O,sat_, and *C*_N_2_O,sat_ is calculated using eq 3 from ref (112):
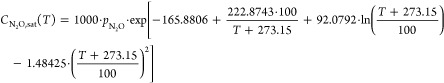
3where *T* is the temperature (in °C) and *p*_N_2_O_ is the partial pressure of N_2_O in the atmosphere. We use the yearly data of global *p*_N_2_O_ estimates for the period 1750–2010 from the Fifth Assessment Report (AR5) of IPCC.^1^

#### 2.2.2 Inland-Water N_2_O Dynamics

For every waterbody type within a grid cell, changes in the amount of inland-water N_2_O (dN_2_O) during the time step (d*t*) can be expressed in a mass-balanced manner as
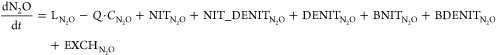
4where L_N_2_O_ is the N_2_O input to the waterbody (from headwaters, upstream grids, and external groundwater input L_N_2_O_grw_ and atmospheric deposition input L_N_2_O_atm_), *Q* is the discharge (m^3^·h^–1^), *C*_N_2_O_ is the inland-water dissolved N_2_O concentration (mol·m^–3^), the net N_2_O production includes that from nitrification (NIT_N_2_O_), nitrifier denitrification (NIT_DENIT_N_2_O_), and denitrification (DENIT_N_2_O_, which can be positive in the case of net N_2_O production as intermediate during incomplete denitrification, or negative in the case of net N_2_O consumption via complete denitrification) in the water column and that from nitrification (BNIT_N_2_O_) and denitrification in the bed sediment (BDENIT_N_2_O_), and EXCH_N_2_O_ is the N_2_O exchange at the water–atmosphere interface (Figure 1). The reaction rates of each inland-water process are subject to change depending on the contemporaneous environmental conditions (such as temperature, hydrology, concentrations of different N forms and oxygen, sediment dynamics, and coupled interactions; see Table S1) specific to that time, location, and waterbody. Further details on the equations (Table S1) and parameters (Table S2b) used in the model to describe inland-water N_2_O dynamics under local hydrological, temperature, and oxic conditions and loadings of N_2_O and N from various sources are in the SI.

Based on refs (16) and (54), we assume that a constant fraction (*f*_N_2_O_ = 1%) of NO_3_^–^ used as an electron acceptor for organic matter degradation is converted to N_2_O during the denitrification processes DENIT and BDENIT; additional N_2_O consumption via reduction to dinitrogen (N_2_) during denitrification occurs when the NO_3_^–^ concentration is lower than the threshold (K_NO_3_,N_2_O reduc_) of 2.7 μmol L^–1^ and temperature is higher than the threshold (*T*_lim,N_2_O reduc_) of 3.1 °C.^21^ Based on ref (113), we assume that a constant fraction (*f*_N_2_O_ = 1%) of NH_4_^+^ consumption during the nitrification processes NIT and BNIT is converted to N_2_O; based on ref (113), we assume that a constant fraction (*f*_N_2_O_ = 1%) of NH_4_^+^ consumption during the nitrifier denitrification processes NIT_DENIT is converted to N_2_O when the DO concentration is lower than the threshold (*S*_O_2_,NIT_DENIT_) of 1.5 mg L^–1^. NIT_DENIT and N_2_O consumption are combined into DENIT when analyzing the N_2_O flux. DENIT_N_2_O_ thus represents the net production of N_2_O via denitrification in the water column depending on temperature, oxic conditions, and concentrations of N_2_O and NO*_x_*^–^, with positive values indicating the net N_2_O production as intermediate and negative values indicating the net N_2_O consumption/uptake by complete denitrification.

To estimate the N_2_O exchange at the water–atmosphere interface, we use the formalism from Alin et al.^114^
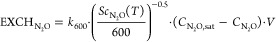
5where *k*_600_ is the gas transfer velocity at the water–atmosphere interface at 20 °C (estimated using flow velocity for streams and windspeed for rivers according to Alin et al.^114^ and using the method of Raymond et al.^85^ for lakes and reservoirs; more details in Table S1), V (in m^3^) is the volume of the waterbody, *C*_N_2_O_ (mol·m^–3^) represents the inland-water N_2_O concentration, and *Sc*_N_2_O_(*T*) is the Schmidt number for N_2_O exchange in freshwater systems at temperature *T* (°C), which according to Wanninkhof^115^ can be expressed as
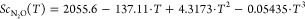
6

### 2.3 Model Performance Assessment

Validation and sensitivity analyses of IMAGE-DGNM simulations of N cycling to changes in inputs and process parameters can be found in an early study.^71^ In this study, we performed a sensitivity analysis of the simulated inland-water N_2_O emissions (including total inland-water emissions and those from different waterbodies) and dissolved N_2_O export to oceans to the model inputs and parameters which are identified as the most important: N_2_O input from groundwater, N_2_O input from atmospheric deposition, N_2_O inland-water production rate via nitrification, N_2_O inland-water production rate via denitrification, total nitrogen, (TN, sum of NH_4_^+^, NO*_x_*^–^, and ON) delivery in inland waters, water discharge, and temperature (Texts S3 and S4 and Figure S10).

To assess the model’s performance at the global scale, we compare the model results with the observed water discharge, N_2_O emission, and concentrations of TN, N_2_O, and DO per site per river basin per year at the overall four levels using available observational data from published databases and literature (Table S3 and Figures S6–S9). First, the temporal patterns of the simulations and observations for each variable among the water discharge, TN, and DO are compared for each of the 16 sites covering different waterbodies and different up-/mid-/downstream locations in the large Mississippi River basin for the period since the 1930s (Figure S6). Second, we compared the root mean squared error (RMSE, calculation method described in Text S2) of the simulated and observed annual average for each variable among water discharge, TN, N_2_O, and DO for each site in global inland waters for the period since the 1920s (Table S3 and Figures S7 and S8). These inland-water systems represent a range in climate, hydrology (sites in up-, mid-, and downstream and different waterbodies), and human activity (land use, dam construction, etc.). Third, we compared the overall N_2_O simulations and observations in global inland waters in terms of different continents, climate zones, and waterbodies at both 1:1 and log_10_ scales (Figure S9). Lastly, we also extensively compare our global N_2_O-emission estimates from different waterbodies for different periods with those reported by existing measurement-based and model-based studies (Sections 3.2, 3.3, 3.5, and 3.6 and Table S4).

## 3 Results and Discussion

### 3.1 Model Performance

Temporally, the historical trends and interannual variability of TN, DO, and water discharge since the 1930s are well captured by the model for 16 different up-/mid-/downstream sites covering different waterbodies in the Mississippi River Basin (Figure S6). Spatially, the simulated water discharge and concentrations of TN, N_2_O, and DO agree with observations per variable per site per river basin per year in global inland waters since the 1920s, with the median RMSE of global simulations and observations lower than 100% for all variables (Figure S8). These observations are from sites covering different climates, population densities, economic levels, degrees of agricultural expansion, hydrological control by dams, waterbodies, and up-/mid-/downstream sites. The differences for sites with RMSE larger than 200% can be partly explained by the different temporal and spatial scales of simulations and observations, such as the limited numbers and inhomogeneous temporal coverage of available observations within a year versus the yearly simulation results. Moreover, the N_2_O simulations and observations in global inland waters show a good agreement in terms of different continents, climate zones, and waterbodies at both 1:1 and log_10_ scales (Figure S9). Our simulated emissions of N_2_O also agree with observations from the literature at both 1:1 and log_10_ scales (Figure S9g,h). In particular, our simulated low N_2_O emissions and even N_2_O sinks in northern high-latitude regions (Figure 4) are consistent with reported N_2_O sinks in boreal aquatic networks;^62,63^ our simulated N_2_O concentrations (0.36–1.1 μg N_2_O-N/L) in the Congo River basin also agree with Congo measurements (0.2–1.1 μg N_2_O-N/L),^116^ with reported undersaturation areas of N_2_O in Congo^117^ reproduced in our simulated N_2_O spatial pattern (Figure 4). This fair agreement between model predictions and independent observations for N_2_O, TN, DO, and hydrological flows in global inland waters gives confidence to the overall methodologically consistent approach.

### 3.2 Changes in the Global Overall Inland-Water N_2_O Fate and Emissions During 1900–2010

**Figure 2:**
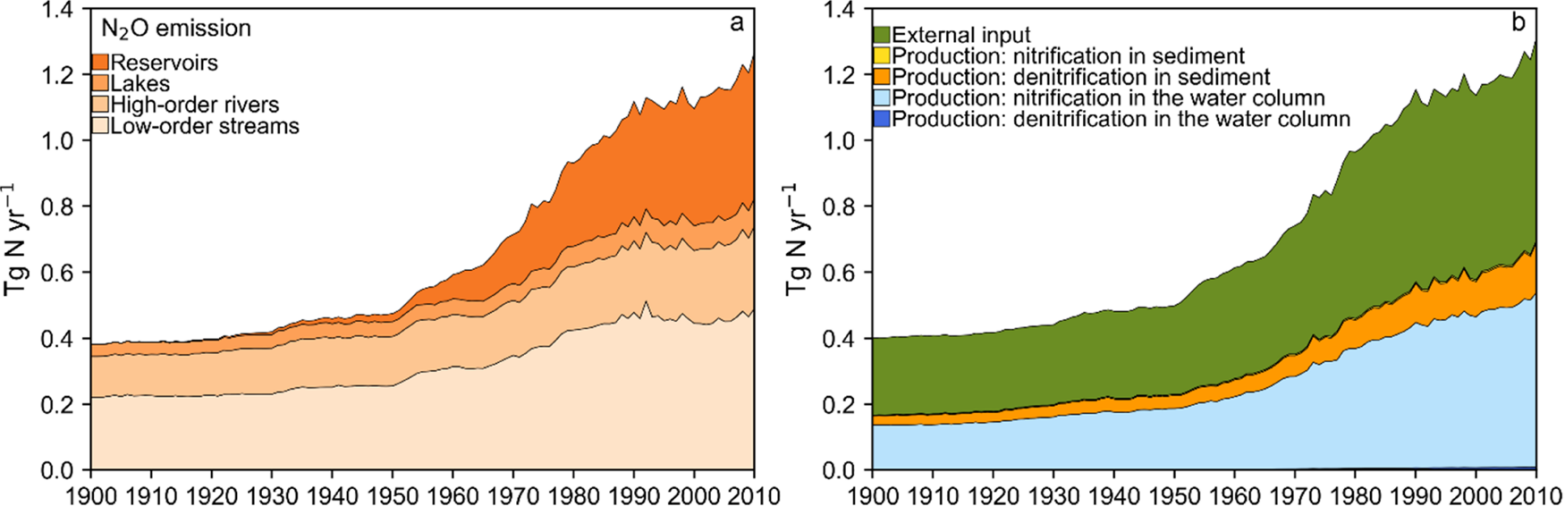
(a) Temporal changes in N_2_O emissions from global inland waters during 1900–2010 and (b) temporal evolution of N_2_O inputs (from the atmosphere and groundwater) and net within-system production (via nitrification and denitrification) in the water column and sediment. Rivers and streams are partitioned into high-order (Strahler order 6 or higher) and low-order (Strahler order 5 or lower) systems. See Figure S2c for the detailed composition of N_2_O inputs from groundwater. The net N_2_O production via denitrification can be positive (net production as intermediate) or negative (net consumption by complete denitrification) for a waterbody type in a grid cell in a year depending on the local temperature, oxic conditions, and concentrations of N_2_O and NO*_x_*^–^ (see Section 2.2.2. Inland-Water N_2_O Dynamics for details).

The simulated global N_2_O emissions from inland waters increased slowly from 0.38 to 0.47 Tg N yr^–1^ before 1950 and rapidly after 1950 to 1.27 Tg N yr^–1^ in 2010 (Figure 2). In contrast to other global estimates for the overall inland waters, we estimate that about half of total inland-water N_2_O emissions during 1900–2010 were from the inflow of N_2_O from groundwater, with its contribution declining from 58% in 1900 to 47% in 2010. The rest was from inland-water N_2_O production.

The total N_2_O and N inputs to inland waters showed similar temporal patterns, with slowly increasing deliveries during the first half of the 20th century and rapid increases afterward. While TN delivery to global inland waters increased from 27 to 68 Tg N yr^–1^ (Figure S2), the total external N_2_O inputs increased from 0.23 to 0.61 Tg N yr^–1^ (Figure 2), mostly from groundwater (Figure S2). Our model estimate of global groundwater N_2_O inputs to inland waters (0.5 Tg N yr^–1^) for the mid-1980s agrees with the estimate (0.4–1.0 Tg N yr^–1^)^70^ based on observations in aquifers.

With increasing riverine N delivery, retention in global inland waters increased from 7 to 27 Tg N yr^–1^,^118^ and the total N_2_O production in global inland waters also increased from 0.17 to 0.70 Tg N yr^–1^ (Figure 2). In 2010, the total production included 0.54 Tg N yr^–1^ in the water column (almost entirely from nitrification) and 0.16 Tg N yr^–1^ in sediments (almost entirely from denitrification).

### 3.3 Role of Different Waterbodies

#### 3.3.1 Streams and Rivers

**Figure 3:**
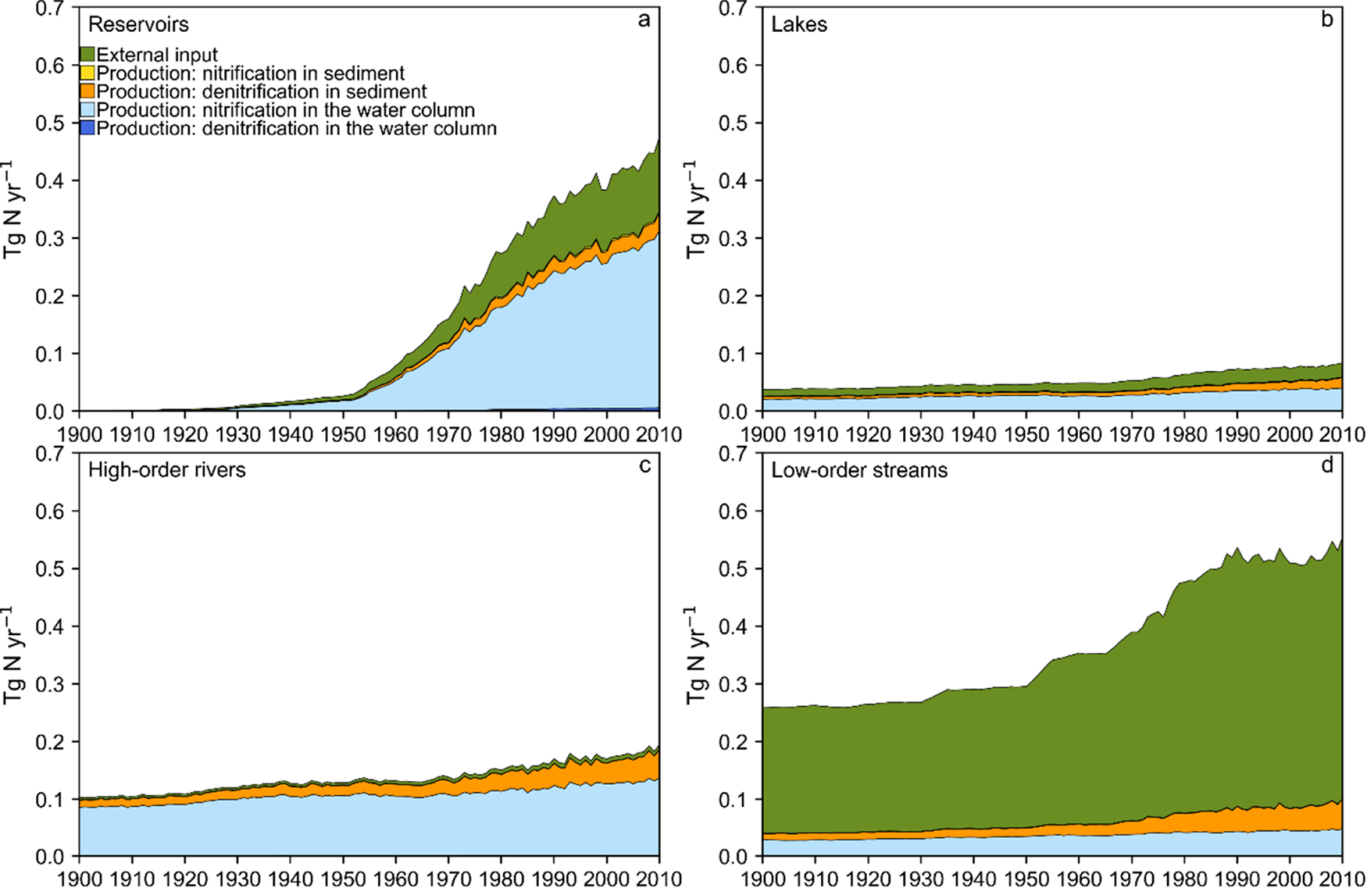
Temporal changes in the N_2_O input and net within-system production through different biogeochemical processes in different waterbodies during 1900–2010: (a) reservoirs, (b) lakes, (c) high-order rivers, and (d) low-order streams. Rivers and streams are partitioned into high-order (Strahler order 6 or higher) and low-order (Strahler order 5 or lower) systems. The net N_2_O production via denitrification can be positive (net production as intermediate) or negative (net consumption by complete denitrification) for a waterbody type in a grid cell in a year depending on the local temperature, oxic conditions, and concentrations of N_2_O and NO*_x_*^–^ (see Section 2.2.2. Inland-Water N_2_O Dynamics for details).

During 1900–2010, the N_2_O emission from low-order streams increased from 0.22 to 0.49 Tg N yr^–1^ and dominated total inland-water N_2_O emissions, but its contribution decreased from 58 to 39% (Figure 2). A large part of the emission from low-order streams stems from groundwater discharge, with the rest from in-stream production (Figure 3). This role of low-order streams as active sites of N_2_O emissions may be related to their long water residence time^119^ and high biogeochemical potential.^120^ Compared with the N_2_O fate in low-order streams, in high-order streams, the role of groundwater input is smaller and that of in-stream production is more important. While the emission of high-order rivers increased from 0.12 to 0.25 Tg N yr^–1^ during 1900–2010, its contribution to global inland-water emissions decreased from 33 to 20% (Figure 2).

The combined N_2_O emissions from high-order rivers and low-order streams increased slowly before 1970, increased rapidly to 0.7 Tg N yr^–1^ in the 1990s, and stabilized afterward. The changes during 1900–1970 were primarily related to the massive land-use changes, with 21% of global natural ecosystems transformed to agriculture, while after 1970, land-use change was less important (4% decline of natural area), but the intensification of agriculture was the main source of N to global inland waters.^121^ Our estimates of riverine N_2_O emissions are lower than those estimated by Seitzinger et al.^56,60^ for the 1990s (1.05 Tg N yr^–1^), by Kroeze et al.^59^ for the mid-1990s (1.26 Tg N yr^–1^), and by IPCC^57,122−124^ for the year 1989 (1.6 Tg N yr^–1^). The differences are primarily related to differences in N loadings because these earlier studies were based on estimated N inputs to global river basins exceeding our estimates by 56–84% (due to their not separating terrestrial from in-stream loss processes) and consequently they obtained higher emission estimates. However, our estimate (0.67 Tg N yr^–1^) is similar to that of Beaulieu et al.^54^ (0.68 Tg N yr^–1^) for the mid-1990s. Our estimate is higher than a recent study (0.07 Tg N yr^–1^)^125^ based on the upscaling of data for the period 1960–2015, and the difference may be due to ignoring the multidecadal increase therein. Yao et al.^25^ reported lower N_2_O emissions of 0.07 Tg N yr^–1^ in 1900 and 0.28 Tg N yr^–1^ in the 2000s for global rivers and streams, compared to our estimates of 0.35 and 0.67 Tg N yr^–1^, respectively. While Yao et al.^25^ presented N_2_O inputs from groundwater discharge for the 2000s (0.39 Tg N yr^–1^) similar to our estimate (0.43 Tg N yr^–1^), their estimate of in-stream N_2_O production in rivers and streams is much lower (0.05 versus our 0.25 Tg N yr^–1^). This difference may be due to their lower rate of in-stream N_2_O production via nitrification (0.1%) compared to other studies^54,56,113^ and ignoring (1) several important N sources (floodplain vegetation, surface runoff from natural and agricultural ecosystems, some point sources, and aquaculture) to inland waters as substrate for in-stream N_2_O production, (2) N_2_O production in sediments, and (3) N_2_O delivered by lakes and reservoirs connected to streams and rivers. Besides, their N_2_O consumption might be overestimated by using N_2_O reduction rates observed in acidic wetlands for open inland waters.^25^

#### 3.3.2 Reservoirs and Lakes

The emission from reservoirs increased from negligible amounts in 1900 to 0.05 Tg N yr^–1^ in the mid-1950s and accelerated to 0.45 Tg N yr^–1^ in 2010. Its contribution to global inland-water emissions increased from 0.2% to 35% during 1900–2010, and reservoirs became the second largest N_2_O source (exceeding that of high-order rivers) since the early-1970s. The increase in reservoir emission (0.44 Tg N yr^–1^) during 1900–2010 accounts for 50% of the total increase in inland-water emission (0.89 Tg N yr^–1^), indicating that reservoirs are currently an important source of N_2_O to the atmosphere. The rapid increase in the reservoir emission can be attributed to the increased number of reservoirs^80^ as well as increased N_2_O production in reservoirs (from ∼0 to 0.35 Tg N yr^–1^) and external N_2_O inputs (from ∼0 to 0.13 Tg N yr^–1^). The increase in the reservoir N_2_O production is the result of increased annual N_2_O-production rates via nitrification in the water column (by over 700-fold) and denitrification in reservoir sediments (by over 300-fold). As the global reservoir volume rapidly increased from the 1950s onwards (Figure S3a), the trapping of organic matter and accumulation in reservoir sediments also increased.^73^ This enhanced the subsequent decay of the accumulated organic matter in reservoir sediments,^118^ which consumed ambient oxygen and created low-oxygen or even hypoxic conditions^126^ favorable for incomplete nitrification and incomplete denitrification as well as associated N_2_O formation.^37^ This is reflected in the resulting rapid increase in N_2_O production in global reservoirs in Figure 3a, which agrees with reports in the literature.^29−37,127^ Consequently, the fraction of the reservoir N_2_O production in global inland-water N_2_O production rapidly increased from 0.3% in 1900 to 40% in the early-1970s, exceeded that of high-order rivers and became the largest among all waterbodies since then, and reached 50% during 2000–2010.

During 1900–2010, the N_2_O emission from lakes increased from 0.04 to 0.08 Tg N yr^–1^ but their contribution to total inland-water emissions decreased from 9 to 7% (Figure 2). Lake N_2_O emission is primarily supported by lake N_2_O production (accounting for 70% on average), with the rest from external N_2_O inputs (mainly from groundwater) (Figure 3). The combined N_2_O emissions of 0.53 Tg N yr^–1^ from lakes and reservoirs in 2010 are close to 0.63 Tg N yr^–1^ estimated by Soued et al.^63^ based on observations, but our estimate of 0.43 Tg N yr^–1^ in 2000 is higher than 0.06 Tg N yr^–1^ estimated in recent studies,^65,66^ possibly because they excluded N_2_O from groundwater, increased reservoir volume, and enhanced N_2_O formation under reservoirs’ changed biogeochemical conditions, and they estimated N transformation fluxes much lower than other studies (e.g., for nitrification, 17 Tg N yr^–1^ by Maavara et al.^66^ versus 42 Tg N yr^–1^ by Seitzinger and Kroeze^60^ and 46 Tg N yr^–1^ in our study).

### 3.4 Spatial Patterns of Inland-Water N_2_O Emissions

**Figure 4:**
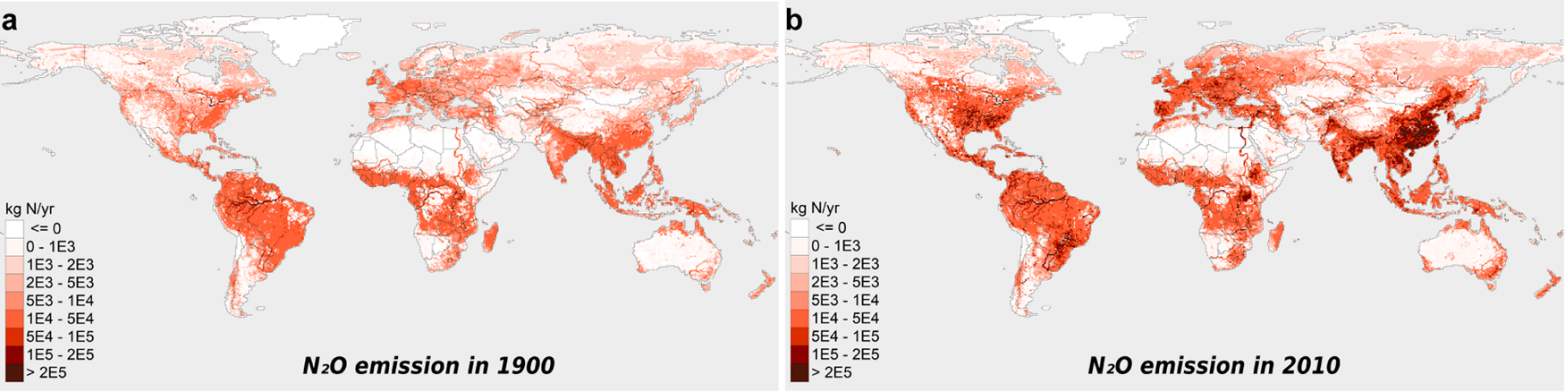
Spatial distributions of the total N_2_O emissions (kg N yr^–1^ per 0.5 × 0.5° grid) from global inland waters in (a) 1900 and (b) 2010.

During the period 1900–2010, the N_2_O emissions increased in most inland waters, with the largest increases in southern and eastern Asia (e.g., Yangtze, Pearl, Amur, Mekong, Salween, Ganges), Europe (e.g., Danube, Loire, Rhine, Rhone), and eastern North America (e.g., Mississippi, St. Lawrence, Missouri) (Figures 4 and S13) as a result of large increases in both groundwater N_2_O input and *in situ* N_2_O production (Figures S11 and S12), especially in and downstream of population centers, agricultural production regions, and coastal areas with increasing N loads. For example, the currently top three most populous countries China (eastern Asia), India (southern Asia), and USA (North America) had 3-fold to 4-fold increases in their populations and 3-fold to 9-fold increases in their total N loads (dominated by agricultural sources) to surface waters during 1900–2010.^10^ This N_2_O spatial pattern is consistent with the high-riverine-N_2_O areas predicted by Turner et al.^18^ and Yao et al.^25^ Moreover, we find that large N_2_O increases are related to reservoir construction areas (Figure 4).

Most of the increases in inland-water N_2_O emissions from these areas mainly occurred after 1950 (Figures 2 and S13) due to massive land-use changes, rapidly developing and intensifying agriculture, larger reservoir volume particularly after 1950, and increasing wastewater discharge along with growing populations (Figure S3). For example, between 1950 and 2010, the global population nearly tripled, and the reservoir volume increased more than 10-fold (Figure S3). After 1990, the N_2_O emissions in Europe, southeastern North America, tropical South America, Africa, and northern Asia stabilized or decreased, while those in eastern and southern Asia continued increasing rapidly (Figures 4 and S13).

Northern high-latitude regions (e.g., Greenland, Mackenzie, Nelson, and Albany rivers and downstream sectors of Ob′ and Yenisey rivers) had low N_2_O emissions and some acted even as N_2_O sinks because of low water temperatures (limiting production^103^ (Figure S12a,b) and increasing N_2_O solubility^112^) and low N_2_O and N (particularly NH_4_^+^ and NO*_x_*^–^) inputs to surface water (Figures S11 and S12). These patterns are consistent with reported N_2_O sinks in boreal aquatic networks.^62,63^ Arid and semiarid river basins (e.g., Nile and Niger in Africa, inland lakes in Oceania, central and southwestern Asia, and western Americas), where wet deposition rates, groundwater discharge, surface runoff, and their N and N_2_O deliveries are low and N_2_O production is limited, also had low N_2_O emissions (Figures S11 and S12).

### 3.5 N_2_O Budget along the River Continuum

**Figure 5:**
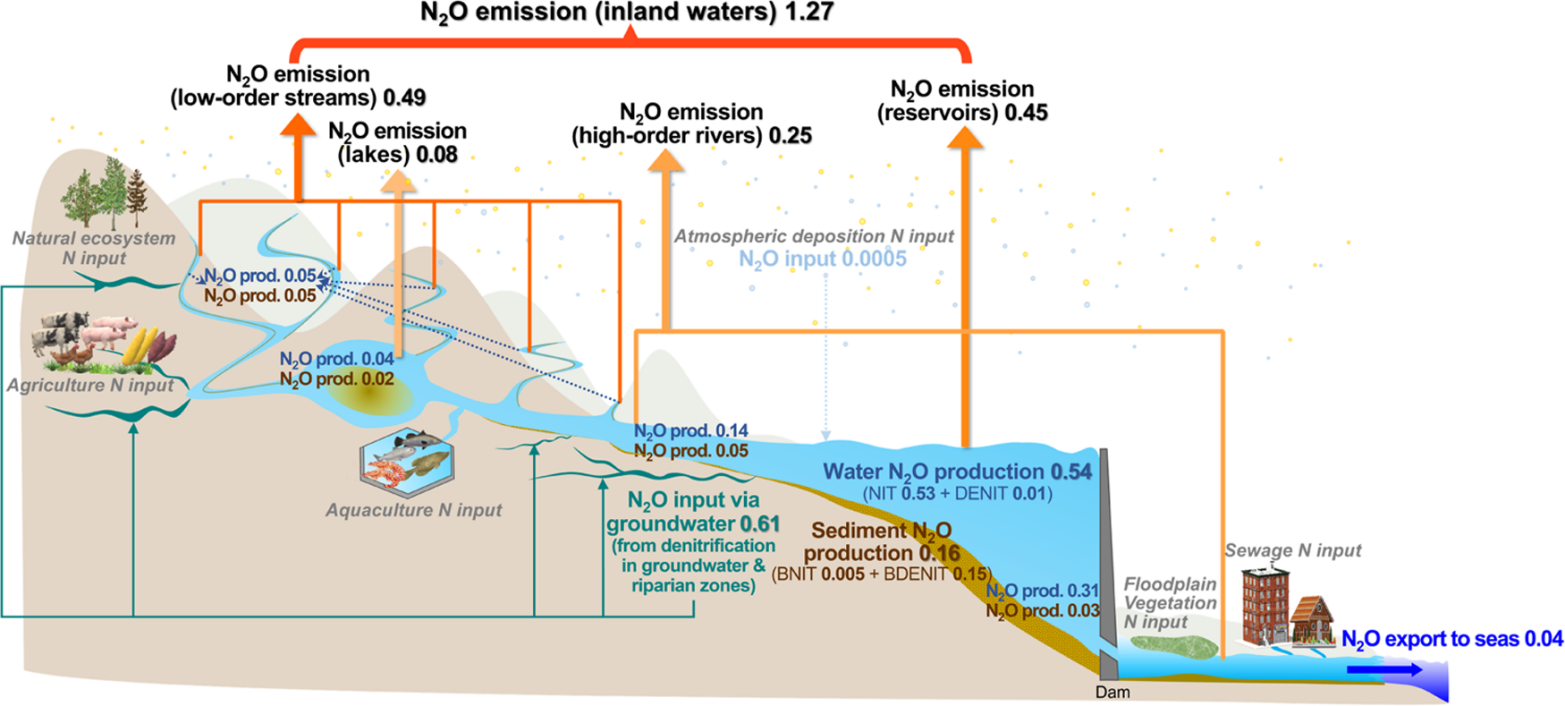
Global N_2_O budget along the aquatic continuum from land to sea in 2010 (in Tg N yr^–1^). The blue and brown fonts indicate the net-N_2_O-production estimates in the water column and sediment, respectively. NIT and DENIT indicate the net inland-water N_2_O production through nitrification and denitrification in the water column, respectively, and BNIT and BDENIT indicate those through nitrification and denitrification in sediment, respectively.

According to the overall freshwater N_2_O budget (Figure 2) and their sensitivities to the key inputs and processes (Figure S10 and Text S4), inland waters are both conduits for the emission of groundwater N_2_O to the atmosphere and compartments of active *in situ* N_2_O production, which is elevated by the increasing anthropogenic N loading (Figure S2). This is consistent with previous reports for streams and rivers.^16,54,70^ Similar to the finding for freshwater methane emission,^128^ we find that groundwater input to low-order streams is high and constitutes their major source of N_2_O emission to the atmosphere, while in high-order rivers, lakes, and reservoirs, inland-water production dominates (Figures 3 and S10 and Text S4). The increase in the global inland-water N_2_O emission by 0.89 Tg N yr^–1^ is balanced by increased N_2_O inputs (0.38 Tg N yr^–1^) from groundwater and increased N_2_O production (0.53 Tg N yr^–1^), mainly in reservoirs. Furthermore, the complete N_2_O budget across different waterbodies along the aquatic continuum also reveals spatial inconsistencies between N_2_O emission and N_2_O inputs or production (Figures 3 and 5). This indicates that where N_2_O is input or produced may not always be where N_2_O is emitted, and that inland systems may receive N_2_O from upstream or export N_2_O to downstream waterbodies. For example, in 2010, N_2_O transport from low-order streams and reservoirs to downstream systems was 0.06 and 0.03 Tg N yr^–1^, respectively, while high-order rivers and lakes vented N_2_O from upstream systems (0.05 and 0.001 Tg N yr^–1^, respectively). The global inland-water N_2_O input and production totaled 1.31 Tg N yr^–1^ and were slightly higher than global emission (1.27 Tg N yr^–1^) because 0.04 Tg N yr^–1^ was exported as dissolved N_2_O by rivers to coastal oceans.

This riverine export of dissolved N_2_O increased from 0.02 to 0.04 Tg N yr^–1^ during 1900–2010 and equaled 3–5% of the N_2_O emission from inland waters (Table S4 and Figure 5). Rapid increases occurred during 1970–2000. Our estimates of river N_2_O export are much lower than those estimated by regression-based models for the same years: our 0.025 Tg N versus 0.15 Tg N with global NEWS^26^ for 1970, our 0.032 Tg N versus 0.22 Tg N with N-model^60^ for 1990, our 0.033 Tg N versus 0.25 Tg N with NEWS-DIN^59^ for 1995, and our 0.035 Tg N versus the range of 0.1–0.6 Tg N yr^–1^ with global NEWS^26^ for 2000 (Table S4). These differences can be attributed to differences in the N load in rivers, as discussed before. However, our estimate for the year 2000 is closer to that of Yao et al.^25^ for the 2000s (0.07 Tg N yr^–1^).

### 3.6 Implications for Global N_2_O Budget

Intensification of agriculture and industry has substantially elevated the biogeochemical cycling of N^10−14^ and increased the atmospheric concentration of N_2_O globally.^1,2,14^ Using both bottom-up and top-down approaches, Tian et al.^7^ estimated that the global N_2_O emissions increased from 15.5 (12.1–20.9) Tg N yr^–1^ in the 1980s to 15.9 (12.2–21.7) Tg N yr^–1^ in the 1990s, 15.9–16.4 (12.3–22.4) Tg N yr^–1^ in the 2000s, and 16.9–17.0 (12.2–23.5) Tg N yr^–1^ during 2007–2016, with rather constant emissions from inland waters, estuaries, and coastal waters of 0.7 (0.5–1.0) Tg N yr^–1^ during the 1980s–2000s and a slight increase to 0.8 (0.5–1.1) Tg N yr^–1^ during 2007–2016.^1,7^

Our results indicate that N_2_O emissions from global inland waters increased from 0.93 to 1.27 Tg N yr^–1^ during 1980–2010, accounting for 6–7% (ranging 4–12%) of the reported global total emissions during the same periods, equivalent to about one-third (22–75%) of that from agricultural soils (Table S5).^3,7^ In the latest simulated year (2010), inland waters contributed 7 (5–10)% to global N_2_O emissions. Our N_2_O emission estimates for inland waters are not only higher than those of IPCC AR6^1^ but also show a steady increase. Assuming that inland-water N_2_O emission in the early 1900s is “natural” and that its increase of 0.89 Tg N yr^–1^ during 1900–2010 is “human-derived”, the human-derived inland-water N_2_O emission thus accounts for 12% (8–19%) of total anthropogenic sources to the atmospheric N_2_O during 2007–2016 estimated by Tian et al.^7^ Moreover, our estimates do not include emissions from estuarine and coastal waters.

Murray et al.^129^ provided a bottom-up, data-based N_2_O-emission estimate from coastal systems of 0.31 (ranging 0.15–0.91) Tg N yr^–1^. Coastal N_2_O emission is supported by both river export of dissolved N_2_O and production within the coastal zone. Assuming that the river export of dissolved N_2_O to coastal waters is entirely vented to the atmosphere (0.04 Tg N yr^–1^), then we estimate roughly by the difference that the emission due to coastal N_2_O production is 0.27 Tg N yr^–1^. Part of this coastal N_2_O production is supported by natural riverine N and oceanic N inputs, while another part can be attributed to anthropogenic N inputs. This partitioning is beyond the scope of this study.

With a conservative assumption that coastal N_2_O production has not been impacted by global warming, increasing hypoxia, and elevated N delivery via rivers and atmosphere, we estimate that N_2_O emissions from global inland, estuarine, and coastal waters increased from 0.7 (0.5–1.3) to 1.6 (1.4–2.2) Tg N yr^–1^ during 1900–2010, and the emissions in 2010 imply a 9–11% contribution to global total N_2_O emissions during 2007–2016 (Table S5). Therefore, the considerable N_2_O fluxes from inland, estuarine, and coastal waters (and their spatial and temporal variabilities) should be properly quantified and included in the global N_2_O budgets. Despite using different approaches, our estimate of 1.4 (1.3–2.0) Tg N yr^–1^ in 1990 was close to that of Seitzinger and Kroeze^60^ (1.3 Tg N yr^–1^) for that period. However, our new estimate is higher than the 0.6 (0.1–2.9) Tg N yr^–1^ reported by IPCC AR5^3^ and 0.8 (0.5–1.1) Tg N yr^–1^ by Tian et al.^7^ and IPCC AR6,^1^ possibly because of the consideration of the long-term changes in groundwater and reservoir contributions in this study.

The rapid increase in inland-water N_2_O emissions is mainly attributed to increases in groundwater N_2_O discharge and reservoir N_2_O production. The groundwater N_2_O discharge is primarily related to N losses from agricultural land. The travel time of groundwater may range from years to decades and longer. Groundwater legacy^130^ of N that entered the system from historical N management years to decades ago, and N_2_O formed in groundwater during transport, can reduce the efficiency of current efforts to mitigate human N_2_O emissions. N_2_O emissions from reservoirs are related to increasing N loading of rivers, increasing reservoir volume, and accumulation of organic-rich material in reservoirs that create conditions prone to N_2_O formation. Considering the continuing increase in N inputs to aquatic environments from human activities in many world regions,^131^ the groundwater legacy, and expected future dam construction^132^ and oxygen depletion in aging reservoirs, the increase in N_2_O emissions from inland waters will persist in the coming decades.

### 3.7 Future Improvements and Implications for Freshwater N_2_O Research

This study provides an integrated, mechanistic view, and consistent quantification of the spatiotemporal changes in the global freshwater N_2_O budget in the Anthropocene, which can only be achieved with (i) a form-explicit N and N_2_O process representation and (ii) spatially explicit and dynamic N_2_O inputs and environmental forcings. The long-term simulations of IMAGE-DGNM show good agreement with available N, N_2_O, DO, and discharge observations since the 1920s, and the simulated global-scale N_2_O-emission estimates agree with the results of some studies based on various approaches. The differences between our estimates and some recent studies can be explained by the differences in estimates of N inputs and inland-water process flows, representations of reservoir and groundwater contributions, and considerations of temporal changes and spatial heterogeneity.

Our model assessment and sensitivity analysis show that improvements in model input parameters and spatiotemporal resolution could result in an improved description of inland-water N_2_O fluxes and better agreement with observations (Text S5). The sensitivity analysis clearly points to the importance of groundwater N_2_O input on the inland-water N_2_O-emission results, while groundwater N_2_O inputs are an important source of uncertainty in terms of their spatial distribution and temporal variability, which needs to be improved to provide more robust N_2_O quantifications and spatial distributions. Some studies (e.g., on urban rivers or lakes) report *f*_N_2_O_ higher or lower than the 1% used for estimating inland-water N_2_O production processes,^28,133−136^ which indicates associated uncertainty. At some locations, the soil-produced N_2_O may enter groundwater and surface waters,^137^ which may increase inland-water N_2_O emissions but is not considered in this study. Moreover, IMAGE-DGNM estimates a smaller low-order stream area than refs (85) and (138), which may lead to an underestimation of low-order streams’ contribution. A better description of the headwater and other waterbody hydrology, for instance from HydroSheds^139^ and HydroLakes^140^ data sets, will improve our inland-water N_2_O estimate. Finally, this study simulates annual fluxes, while for many processes, it may be important to analyze fluxes at shorter time scales. A shorter (monthly or daily) time step would allow the simulation of seasonal and monthly N and N_2_O fluxes to better understand their dynamics and feedback on human activities and climate change. Such an improved model would be instrumental in not only understanding the role of inland waters in the global N_2_O source-to-sink budget and N and carbon cycling, but also providing better information to policymakers for future climate projections and efficient emission-mitigation strategy development. Future research may need to take into account the spatial heterogeneity and long-term changes in the inland-water N_2_O fate revealed in this study, for example, by monitoring inland-water N_2_O changes regularly in the long term and by combining modeling approaches with isotope measurements.

## Supporting Information Available

Summary of observational ratios of N_2_O production to groundwater denitrification; method of statistical comparison (RMSE) of observation and simulation; method and results of sensitivity analysis; uncertainty and limitations; global average N_2_O atmospheric concentration since 1750; N (of different forms) and N_2_O inputs to global inland waters from different sources; global reservoir volume, population, wastewater N discharge to surface water, gross production value of agriculture, manure production, and fertilizer use; IMAGE-DGNM scheme of water and nutrient flows in the soil–hydrosphere system; validation of long-term temporal patterns of water discharge, TN, and DO for different up-, mid-, and downstream sites covering different waterbodies in Mississippi since the 1930s; distribution of sites of observational data from data sets and literature for the validation of water discharge, TN, N_2_O, and DO in global inland waters since the 1920s; validation: RMSE of simulations and observations for water discharge, TN, N_2_O, and DO at monitoring sites in global inland waters for long time series since the 1920s; validation: comparison of simulated and observed N_2_O concentrations per site per year in global inland waters since the 1970s; sensitivity results for variations in model parameters; spatial distributions of N_2_O inputs to global inland waters in 1900 and 2010 simulated by IMAGE-DGNM; spatial distributions of N_2_O production and multiple-form N loads in global inland waters in 1900 and 2010 simulated by IMAGE-DGNM; temporal changes in spatial distributions of the global freshwater N_2_O emissions during 1900–2010 simulated by IMAGE-DGNM; methodology and parameters for inland-water N_2_O dynamics in IMAGE-DGNM; summary of observation data from databases and literature used for comparison with model outputs of concentrations of TN, N_2_O, DO, and water discharge per variable per site per river basin in global inland waters for the period since the 1920s; comparison with reported estimates of global N_2_O emissions from inland waters; and contribution of inland waters to global N_2_O emissions in the 1980s, 1990s, 2000s, and 2010s (PDF)
