# Supplementary material for: Inland Waters Increasingly Produce and Emit Nitrous Oxide
Source: Environ Sci Technol. 2023 Aug 30;57(36):13506–19. doi: 10.1021/acs.est.3c04230 (PMC10501125; doi:10.1021/acs.est.3c04230)
Supplement: Supplementary file 1 — es3c04230_si_001.pdf [file es3c04230_si_001.pdf]

Supporting Information for

Inland waters increasingly produce and emit nitrous oxide

Junjie Wang[1], Lauriane Vilmin[1,2], José M. Mogollón[3], Arthur H.W. Beusen[1,4], Wim J. van Hoek[1], Xiaochen Liu[1]*, Philip A. Pika[5], Jack J. Middelburg[1], Alexander F. Bouwman[1]*

[1] Department of Earth Sciences, Utrecht University, Princetonlaan 8a, Utrecht, Netherlands, 3584CB

[2] Deltares, P.O. Box 177, Delft, Netherlands, 2600MH

[3] Department of Industrial Ecology, Leiden University, Leiden, Netherlands, 2300RA

[4] PBL Netherlands Environmental Assessment Agency, P.O. Box 30314, Hague, Netherlands, 2500GH

[5] Faculty of Science, Earth and Climate, Free University of Amsterdam, de Boelelaan 1105, Amsterdam, Netherlands, 1081HV

*Corresponding author: Junjie Wang (Email: j.wang3@uu.nl), Xiaochen Liu (Email: x.liu@uu.nl)

Supporting Information includes 41 pages, 13 figures, and 5 tables.

Contents:

Supporting Texts:

Text S1. Summary of observational ratios of N_2O production to groundwater denitrification

Text S2. Method of statistical comparison of observation and simulation: Root Mean Squared Error

Text S3. Method of sensitivity analysis

Text S4. Summary of sensitivity analysis results

Text S5. Uncertainty and limitations

Supporting Figures:

Figure S1. Global average N_2O atmospheric concentration during 1750-2020.

Figure S2. N (of different forms) and N_2O inputs to global inland waters from different sources.

Figure S3. Global reservoir volume, population, wastewater N discharge to surface water, gross production value of agriculture, manure production and fertilizer use.

Figure S4. IMAGE-DGNM scheme of water and nutrient flows in the soil-hydrosphere system.

Figure S5. Range of observation-based ratios of N_2O production to groundwater denitrification.

Figure S6. Validation of long-term temporal patterns of water discharge, TN and DO for different up-, mid- and downstream sites covering different waterbodies in Mississippi since the 1930s.

Figure S7. Validation: Distribution of sites of observational data from datasets and literature for validation of water discharge, TN, N_2O and DO in global inland waters since the 1920s.

Figure S8. Validation: RMSE of simulations and observations for water discharge, TN, N_2O and DO at monitoring sites in global inland waters for long time series since the 1920s.

Figure S9. Validation: Comparison of simulated and observed N_2O concentrations and emissions per site per year in global inland waters since the 1970s.

Figure S10. Sensitivity results for variations in model parameters.

Figure S11. Spatial distributions of N_2O inputs to global inland waters in 1900 and 2010 simulated by IMAGE-DGNM.

Figure S12. Spatial distributions of N_2O production and multiple-form N loads in global inland waters in 1900 and 2010 simulated by IMAGE-DGNM.

Figure S13. Temporal changes in spatial distributions of the global freshwater N_2O emissions during 1900-2010.

Supporting Tables:

Table S1. Methodology of inland-water N_2O dynamics in IMAGE-DGNM

Table S2. Parameters used to simulate inland-water N_2O dynamics in IMAGE-DGNM.

Table S3. Summary of observation data from databases and literature used for comparison with model outputs of TN and DO concentrations, N_2O concentration and emission, and water discharge per variable per site per river basin in global inland waters for the period since the 1920s.

Table S4. Comparison with reported estimates of global N_2O emissions from inland waters.

Table S5. Contribution of inland waters to global N_2O emissions in the 1980s, 1990s, 2000s and 2010s

References

Text S1. Summary of observational ratios of N_2O production to groundwater denitrification: range from literature and value used in the model.

The empirical parameter of N_2O production rate via groundwater denitrification (f_{N_2O}) in the model comes from the summary of reported observation-based ratios of N_2O production to groundwater denitrification (i.e., N_2O-N: (N_2-N+N_2O-N)) in literature (Groffman et al., 1998; Groffman et al., 2000; Well et al., 2001; Mookherji et al., 2003; Well et al., 2003; Well et al., 2005b; Weymann et al., 2008; Weymann et al., 2010; Jahangir et al., 2012; Well et al., 2012; Jahangir et al., 2013; Fox et al., 2014; Mander et al., 2014; Gardner et al., 2016; McAleer et al., 2017; Zhou et al., 2018).

Among the total number of 278 of the reported data, the median value of the ratios of N_2O production to groundwater denitrification is 0.010, the average value is 0.041, the lower quartile is 0.002, the upper quartile is 0.041, and outliers account for 14% of all data (Figure S5). The mean value of the data within the range from the lower to upper quartiles is 0.014, which is close to the median of 0.010. Taking ±1 standard deviation as the uncertainty range of data within the lower and upper quartiles, the uncertainty of the ratios of N_2O production to groundwater denitrification is ±0.011 (±76%).

Therefore, in this study, a constant fraction (f_{N_2O}=1%, median of the 278 reported observation-based values) of shallow groundwater denitrification is calculated as the N_2O produced in shallow groundwater, which is transported to surface waters (Figure S4).

Text S2. Method of statistical comparison of observation and simulation: Root Mean Squared Error

DISC-simulated and observed yearly averages are compared for each variable of water discharge and concentrations of TN, N_2O and DO, for each monitoring site in inland waters using data from published databases and literature (Figure S7 and Table S3), for years since the 1920s with at least 4 measurement months (for representativity concerns). At each monitoring site, we assess the discrepancies between simulated and measured results for each variable by calculating the Root Mean Squared Error (RMSE)(Chai and Draxler, 2014) using the equation below:

$$RMSE = \frac{100}{\overline{Obs}} \cdot \sqrt{\frac{\sum_{i=0}^{n}(Sim_i - Obs_i)^2}{n}} \quad \text{(TS2.1)},$$

where Obs_i and Sim_i are the observed and simulated yearly average values of the variable, respectively, $\overline{Obs}$ is the mean of the observations, and n is the number of observations. RMSE values smaller than 50% are considered acceptable (Beusen et al., 2015). RMSE results for validation are summarized in Figure S8.

Text S3. Method of sensitivity analysis

Since our model is based on mass conservation, the modelled global N (including N_2O) fluxes are internally consistent, and temporal changes are the combined result of the changes in hydrology, climate, and N inputs from the land. Therefore, in addition to extensive validation (Figures S6-S9 and Table S3), we perform an analysis of the sensitivity of modelled fluxes to the variation of model input parameters (Figure S10 and Texts S3-S4).

In this study, we use a one-factor method to quantify the sensitivity of model output results to varying input parameter values with a relatively limited number of runs. According to the results shown in the Main text and Supporting Information, the large increase in the global inland-water N_2O emissions for the period 1900-2010 is due to increased N_2O inputs (mainly from groundwater) and increased inland-water N_2O production (mainly via nitrification in the water column and denitrification in sediment) (Figures 2-3). N_2O inputs are mainly from groundwater and minorly from atmospheric deposition (Figure 2), while inland-water N_2O production is closely related to the reactive N availability in waterbodies and N_2O production rates via nitrification and denitrification (Figures 2 and S12). Temperature and discharge are also important environmental factors for N_2O-related transformation and transport processes in inland waters (Figure 1 and Table S1). Therefore, we identify N_2O input from groundwater, N_2O input from atmospheric deposition, N_2O inland-water production rate via nitrification, N_2O inland-water production rate via denitrification, TN delivery in inland waters, discharge and temperature as the most important model input parameters (X_i) to the simulated global inland-water N_2O emissions and export (Y). We perform model runs for sensitivity to the variations in these input parameters (X_i), and examine the results of multi-year averages over the period 1995-2000 for the following output parameters (Y): yearly total N_2O emissions from inland waters, N_2O emission from low-order streams, N_2O emission from high-order rivers, N_2O emission from lakes, N_2O emission from reservoirs, and river dissolved N_2O export to oceans (see Figure S10 below).

In each run, the values of one of the selected model parameters (X_i, except for temperature) are multiplied with a factor of 0.95 or 1.05 (i.e., ±5% variation, X_{im} and X_{ip}, respectively), while the values of other model parameters maintain original default values; for the run of model sensitivity to temperature, an addition of -1°C or 1°C is applied to the default temperature (X_{im} and X_{ip}, respectively), while the values of other model parameters maintain original default values. With the runs for each input parameter performed (with outputs of Y_{io}, Y_{im} and Y_{ip} matching inputs of the default X_{io}, and changed X_{im} and X_{ip}, respectively), the linear regression can be used for evaluating the contribution of the variation in input parameter X_i to the variation in output parameter Y if the coefficient of determination (R^2) is close to 1, i.e., when there is no variation in Y that is not explained with the linear regression model. The output parameter Y corresponding to the variation in input parameter X_i can be expressed using a linear regression approach below:

$$Y = \beta_{\mathrm{i}} X_{\mathrm{i}} + e \qquad \text{(TS3.1)},$$

where β_i is the slope of parameter X_i and e is the intercept of the approximation of Y. The relative variation in Y corresponding to the relative variation in X_i by an increase of 5%, i.e., Cx_i, can be thus expressed as follows:

$$Cx_i = \beta_\mathrm{i}\frac{(X_{ip}-X_{i0})+(X_{i0}-X_{im})}{Y_{i0}}\frac{X_{i0}}{(X_{ip}-X_{i0})+(X_{i0}-X_{m})} * 0.05 = 0.05 * \beta_\mathrm{i}\frac{X_{i0}}{Y_{i0}} \quad \text{(TS3.2)},$$

where Cx_i is independent of units and scale of parameters, a positive Cx_i value indicates that an increased X_i leads to an increased output Y, and a negative Cx_i indicates a decreased output Y with an increased X_i.

Text S4. Summary of sensitivity analysis results

Sensitivity analysis which was earlier conducted for IMAGE-DGNM and the DISC-NITROGEN module revealed the importance of model parameters and input data on the inland-water N cycle (Beusen et al., 2015; Beusen et al., 2016; Vilmin et al., 2020). For example, inputs of multiple N forms and sources influence river N retention and export (Beusen et al., 2016; Vilmin et al., 2020), runoff influences river N delivery, retention and export (Beusen et al., 2015), optimal temperatures for nitrification and denitrification in river basins influence the inland-water transformation of multiple N forms (Vilmin et al., 2020).

In this study, we further analysed the sensitivity of simulated freshwater N_2O emissions (including those from different waterbodies of low-order streams, high-order rivers, lakes and reservoirs) and river export of dissolved N_2O to the variations in N_2O input from groundwater, N_2O input from atmospheric deposition, N_2O inland-water production rate via nitrification, N_2O inland-water production rate via denitrification, TN delivery in inland waters, discharge and temperature (Text S3). The results show that the global total inland-water N_2O emissions are sensitive to N_2O input from groundwater, total reactive N delivery in global inland waters and N_2O inland-water production rate via nitrification, and will increase by 2.1-2.6% as one of these parameters increases by 5% (Figure S10). Total inland-water N_2O emissions are less sensitive to discharge (-1.3%), N_2O inland-water production rate via denitrification (+0.6%), and input from atmospheric deposition (-0.2%). N_2O emission from low-order streams is mainly sensitive to N_2O input from groundwater, and will increase by 4.4% with a 5% increase in N_2O input from groundwater. N_2O emissions from high-order rivers, lakes and reservoirs will increase by 1.0-1.2% with a 5% increase in N_2O input from groundwater, but will increase by 2.4-3.3% with a 5% increase in N_2O inland-water production rate via nitrification and by 2.8-3.8% with a 5% increase in the total reactive N delivery. A 5% increase in N_2O inland-water production rate via denitrification will increase the N_2O emissions from the four individual waterbodies by 0.4-0.9%. This indicates that N_2O emissions from high-order rivers, lakes and reservoirs are mainly sensitive to the total reactive N delivery in global inland waters and N_2O inland-water production rate via nitrification, which are both closely related to the inland-water N_2O production. The dissolved N_2O export also increases (by 3.0, 2.1, 0.7%, and 0.6%, respectively) as the total reactive N delivery, N_2O inland-water production rate via nitrification, N_2O inland-water production rate via denitrification, or N_2O input from groundwater increase by 5%. A 5% increase in water discharge will reduce N_2O emissions from four waterbodies individually and collectively by 1.2-1.3% but will increase river export of dissolved N_2O by 3.3%. An increase in temperature will slightly increase N_2O emissions from four waterbodies individually and collectively (+0.2-1.4%), but slightly reduce river export of dissolved N_2O in turn (-1.5%).

Text S5. Uncertainty and limitations

The discrepancy between simulations and observations can be partly attributed to their different time scales. Though we have used the yearly means of observational data with at least 4 measurement months within the year to reduce relevant influences, available observational data may not be representative due to incomplete temporal coverage or inhomogeneous distribution within each year. Our simulated results are on an annual basis, which may not appropriately capture the pattern of short-term (i.e., daily, monthly or seasonal) observations while some available measurements were only conducted in one or several months (with inhomogeneous distribution within the year) instead of the whole year. Moreover, freshwaters are heavily under-sampled and the lack of representativeness of the few N_2O measurements available before the year 2010 may also contribute to the minor mismatch.

Another reason may be the issue of spatial scales. The spatial input data IMAGE-DGNM can be uncertain due to the coarse resolution, which may have significant influences on the description of N cycling in low-order streams with extremely spatially variable topography (Vilmin et al., 2020) or in draining areas across boundaries of grid cells (Beusen et al., 2015), while the available site-level observations may not represent the average condition of the entire coarse 0.5°×0.5° grid. Moreover, IMAGE-DGNM estimates that low-order streams account for 61-66% of the total inland-water area during 1900-2010, which is lower than the 69% estimated using data from HydroSHEDS (Lehner et al., 2008) by Raymond et al. (2013) and the even higher value by Allen and Pavelsky (2018). This may thus lead to the underestimation of the contribution of low-order streams.

Except for the N, N_2O oxygen, and hydrological flows in global inland waters validated for the period since the 1920s, sensitivity results show that global inland-water N_2O emissions are sensitive to groundwater N_2O input (Figure S10). N_2O was reported to be supersaturated in groundwater compared to the atmosphere, and production (as an intermediate product during the denitrification process of the stepwise reduction of NO_3^- to N_2) or accumulation of N_2O in groundwater, especially under agricultural land cover, was widely reported (Lemon and Lemon, 1981; Bowden and Bormann, 1986; Spalding and Parrott, 1994; Weller et al., 1994; Groffman et al., 1998; Groffman et al., 2000; Well et al., 2005a; Deurer et al., 2008; von der Heide et al., 2008; Jahangir et al., 2012; Jurado et al., 2017). The large uncertainties of the measured ratio of N_2O:denitrification may be subject to vertical and lateral diffusive or convective transport of N_2O in groundwater; i.e., the site where groundwater N_2O is sampled may not necessarily be where N_2O is produced. Besides, N_2O can be produced by nitrification, nitrifier denitrification or denitrification and leached from the unsaturated zone (Spalding and Parrott, 1994; Muhlherr and Hiscock, 1998; Well et al., 2001; DeSimone et al., 2010). Furthermore, N_2O in groundwater (from denitrification) depends on the infiltration of NO_3^- and electron-contributors into the saturated zone in the historical year and denitrification progress during the groundwater transport (Böhlke, 2002; Van Drecht et al., 2003; Keuskamp et al., 2012), which depend on the travel times that are highly variable because groundwater flow to surface water is generally a mixture of water with highly varying travel times. Depending on the local environmental and biogeochemical conditions, denitrification progress can control both the production and consumption of N_2O in groundwater (Bouwman et al., 2013a). Consequently, N_2O production via groundwater denitrification may have a nonlinear relationship with these various controlling factors (Keuskamp et al., 2012).

Though with uncertainties above, our estimate of 0.5 Tg N yr^{-1} of N_2O inputs from groundwater for the mid-1980s is comparable to 0.4-1.0 Tg N yr^{-1} based on observations in aquifers for the same period by Ronen et al. (1988). Moreover, our estimate of N_2O inputs from groundwater to low-order streams of 0.41 Tg N yr^{-1} for the year 2000 is similar to 0.39 Tg N yr^{-1} for the 2000s estimated by Yao et al. (2020). Our estimates of freshwater N_2O concentrations and emissions for the period since the 1970s also agree with observations and some observation-based studies, respectively (Sections 3.2, 3.3, 3.5, and 3.6 in Main text). The differences between our estimates and some recent studies can be explained by the differences in estimates of N inputs and inland-water process flows, representations of reservoir and groundwater contributions, and considerations of temporal changes and spatial heterogeneity.

Overall, the agreement between our simulations and existing observations and estimates gives confidence to the methodologically consistent, process-based approach of IMAGE-DGNM to obtain historical N and N_2O conditions. Furthermore, as the N_2O fluxes in global inland waters simulated by IMAGE-DGNM account for spatial and temporal heterogeneities in N loading and transfers in river networks as well as the hydrological conditions and climate changes that control N transport, transformations and exchange at interfaces, this integrated approach can further be used to identify the controlling factors of N_2O emissions from inland waters regionally and globally over time as next steps, and has the potential to predict future changes in freshwater N_2O emissions under various scenarios.

Figure S1. Global average N_2O atmospheric concentration since 1750

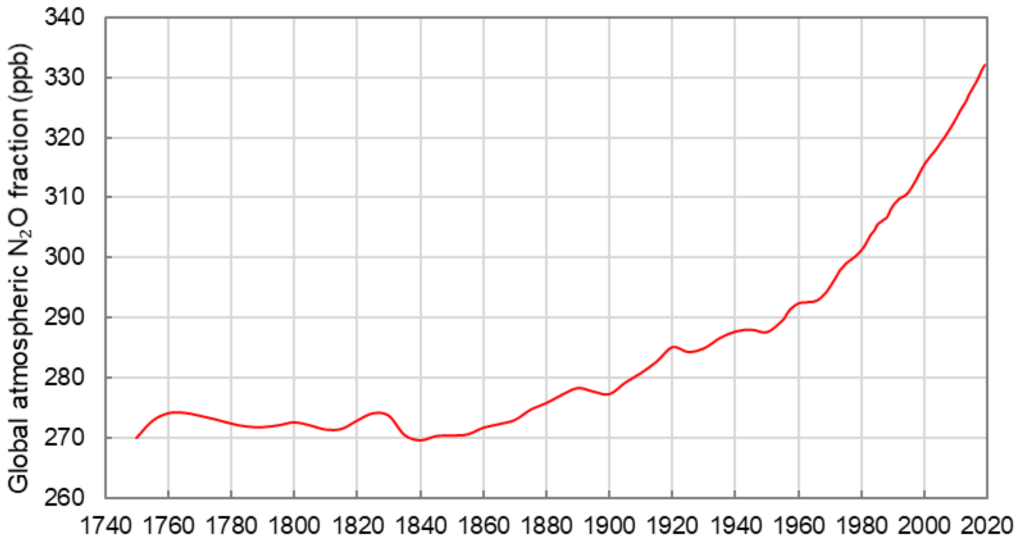

Figure S1. The global average atmospheric N_2O fraction for the period 1750-2020 (unit: ppb). Data derived from the IPCC Sixth Assessment Report (AR6) Climate Change 2021: The Physical Science Basis (IPCC, 2021) and World Meteorological Organization Greenhouse Gas Bulletin 2020 (WMO, 2021).

Figure S2. N (of different forms) and N_2O inputs to global inland waters from different sources.

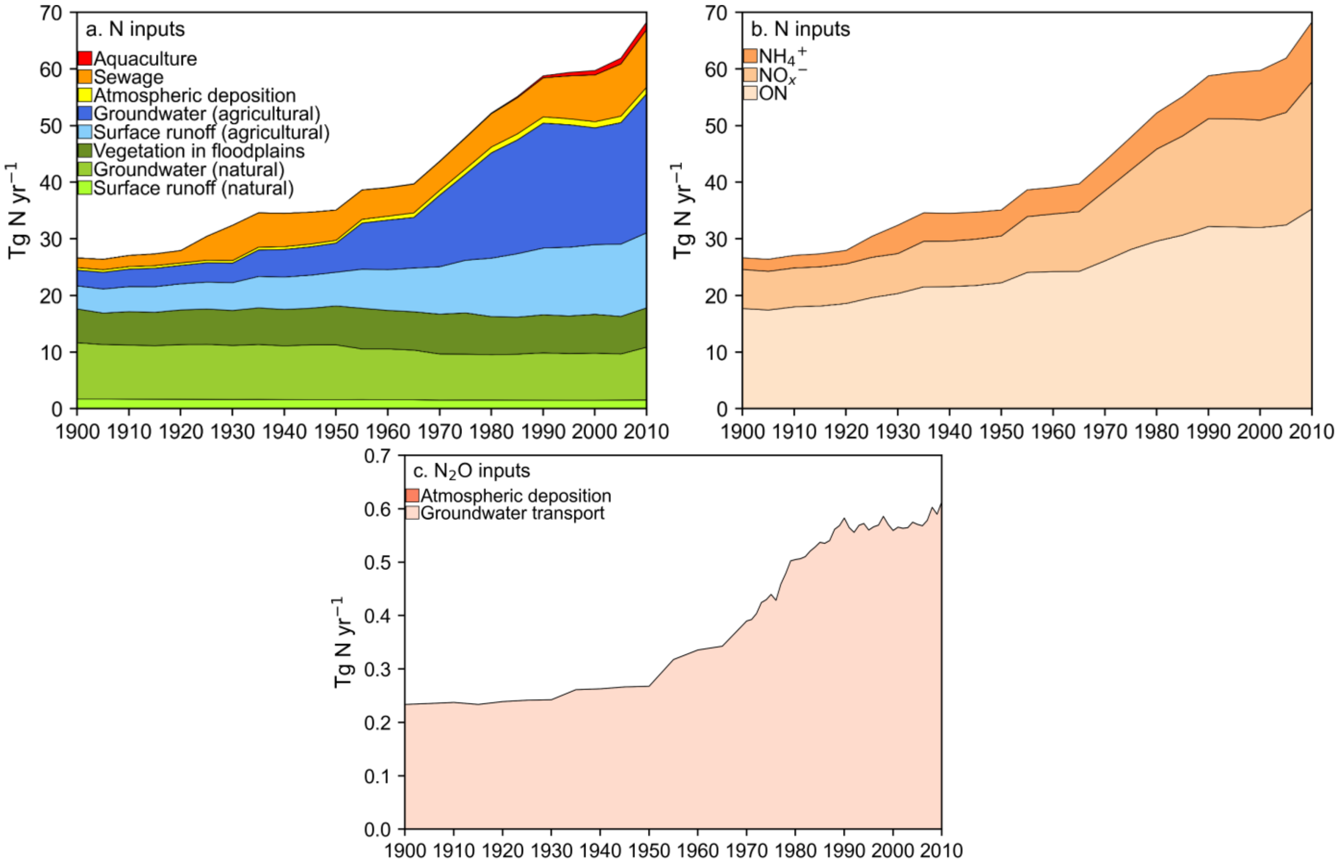

Figure S2. Total nitrogen (TN, excluding dissolved N_2O) inputs to the global river basins simulated by IMAGE-DGNM for the period 1900-2010 (a) from different sources (data from Beusen et al. (2022)), (b) of different major forms including nitrate+nitrite (NO_x^-), ammonium (NH_4^+) and organic nitrogen (ON) (data from Vilmin et al. (2018); Beusen et al. (2022)), and (c) N_2O inputs to the global river basins simulated by IMAGE-DGNM in this study. N_2O input from atmospheric deposition accounted for 0.1-0.2% of the total N_2O inputs during 1900-2010, thus invisible in Figure S2c.

Figure S3. Global reservoir volume, population, gross production value of agriculture and wastewater N discharge to surface water during 1900-2010.

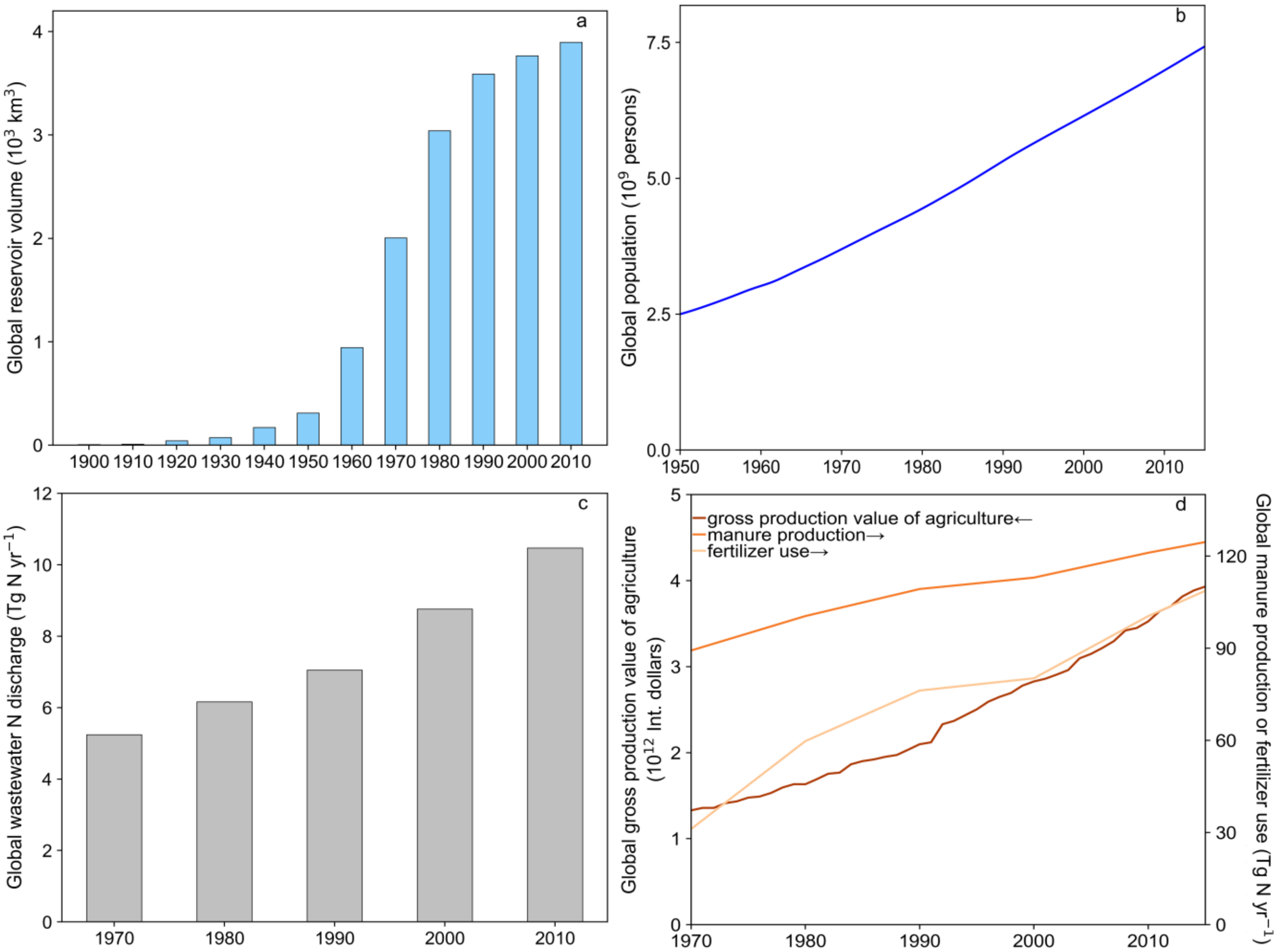

Figure S3. (a) Global reservoir volume for the period 1900-2010 (data from (Lehner et al., 2011)); (b) global population for the period 1950-2018 (data from FAO (2021)); (c) global wastewater N discharge to surface water during 1970-2010 (data from van Puijenbroek et al. (2019)); (d) global gross production value of agriculture, manure production and fertilizer use during 1970-2015 (data from Bouwman et al. (2013b); (2017); FAO (2021)).

Figure S4. IMAGE-DGNM scheme of water and nutrient flows in the soil-hydrosphere system.

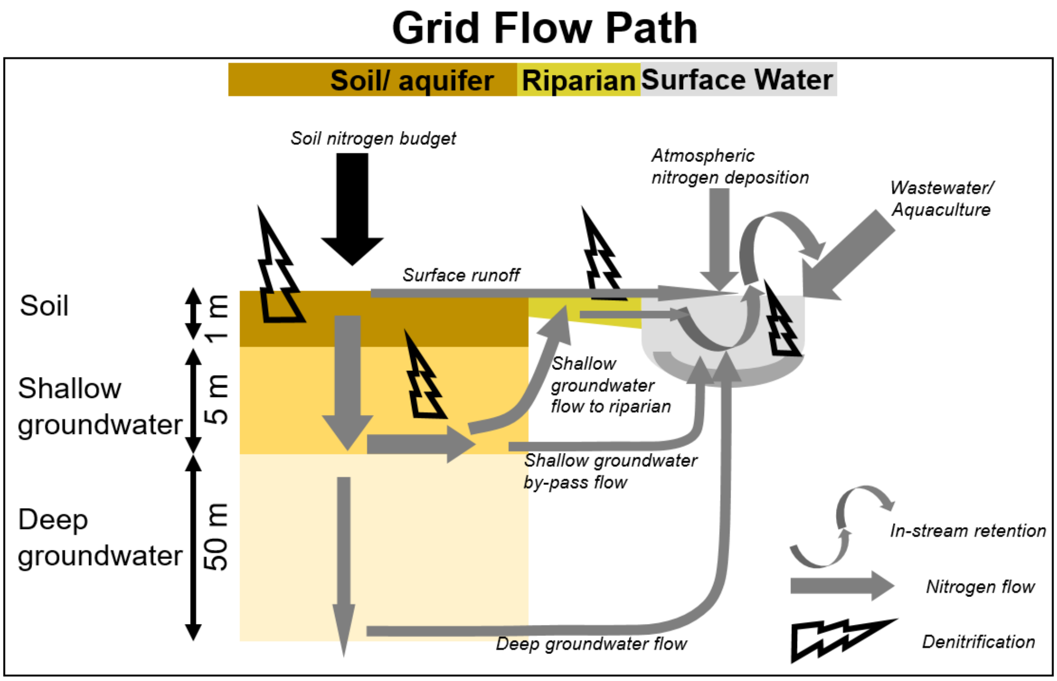

Figure S4. IMAGE-DGNM scheme of water and nutrient flows in the soil-hydrosphere system within a 0.5-by-0.5-degree grid, modified from Beusen et al. (2015).

Figure S5. Range of observation-based ratios of N_2O production to groundwater denitrification

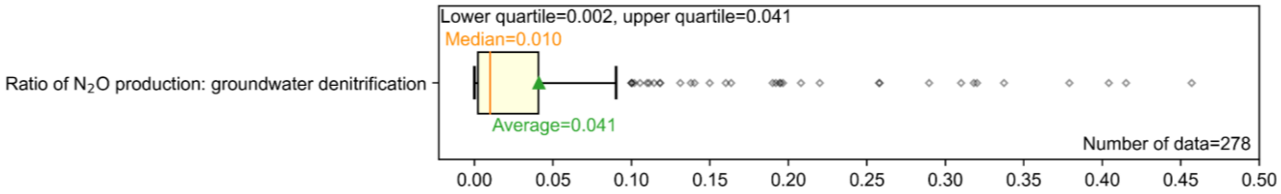

Figure S5. Range of observation-based ratios of N_2O production to groundwater denitrification (f_{N_2O}, i.e., N_2O-N: (N_2-N+N_2O-N), number of data = 278). Observation-based ratios of N_2O production to groundwater denitrification are from Groffman et al. (1998); Groffman et al. (2000); Well et al. (2001); Mookherji et al. (2003); Well et al. (2003); Well et al. (2005b); Weymann et al. (2008); Weymann et al. (2010); Jahangir et al. (2012); Well et al. (2012); Jahangir et al. (2013); Fox et al. (2014); Mander et al. (2014); Gardner et al. (2016); McAleer et al. (2017); Zhou et al. (2018).

Figure S6. Validation of long-term temporal patterns of water discharge, TN concentrations, and DO concentrations for different up-, mid- and downstream stations (covering different waterbodies) within the Mississippi River Basin since the 1930s.

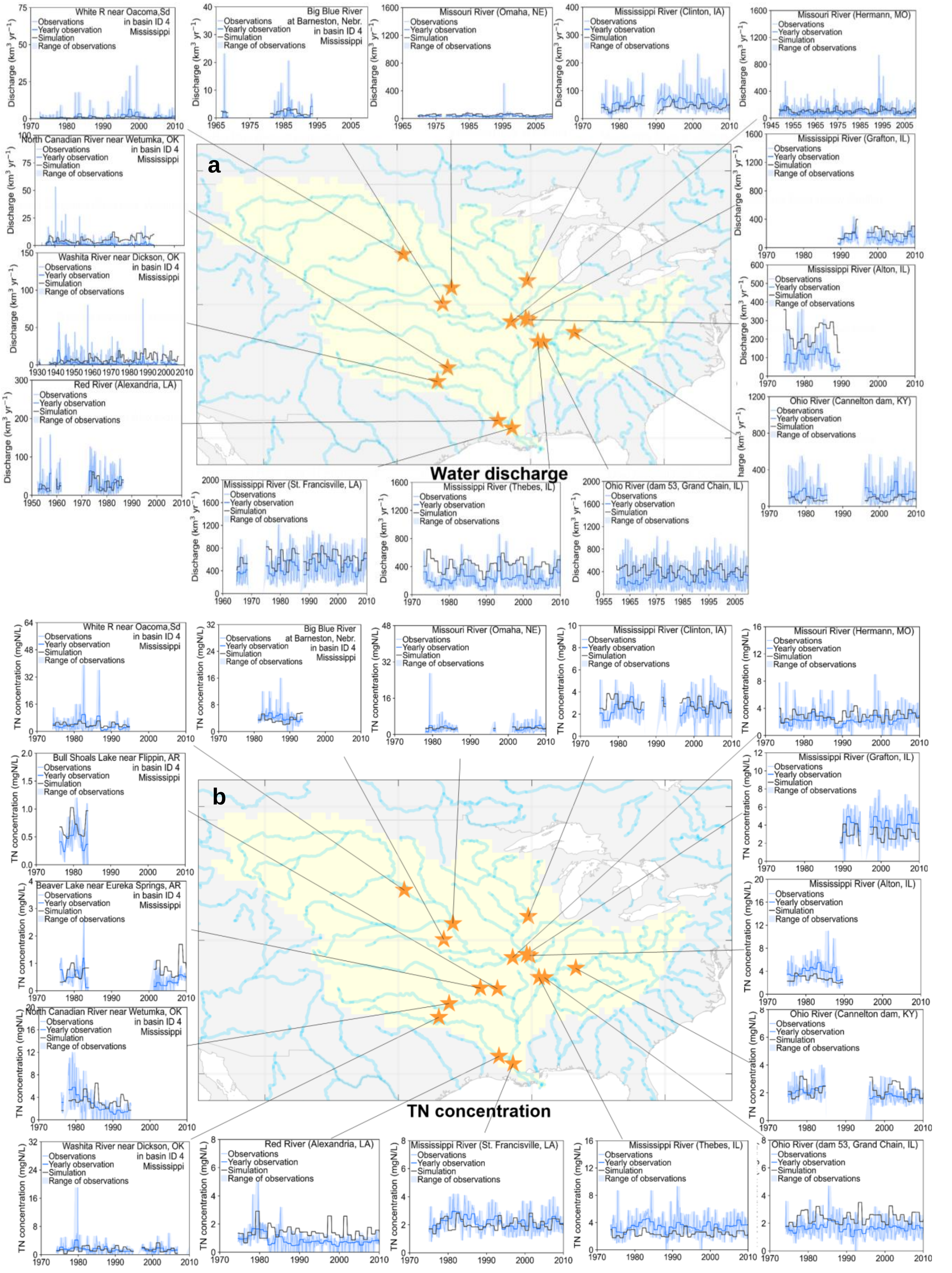

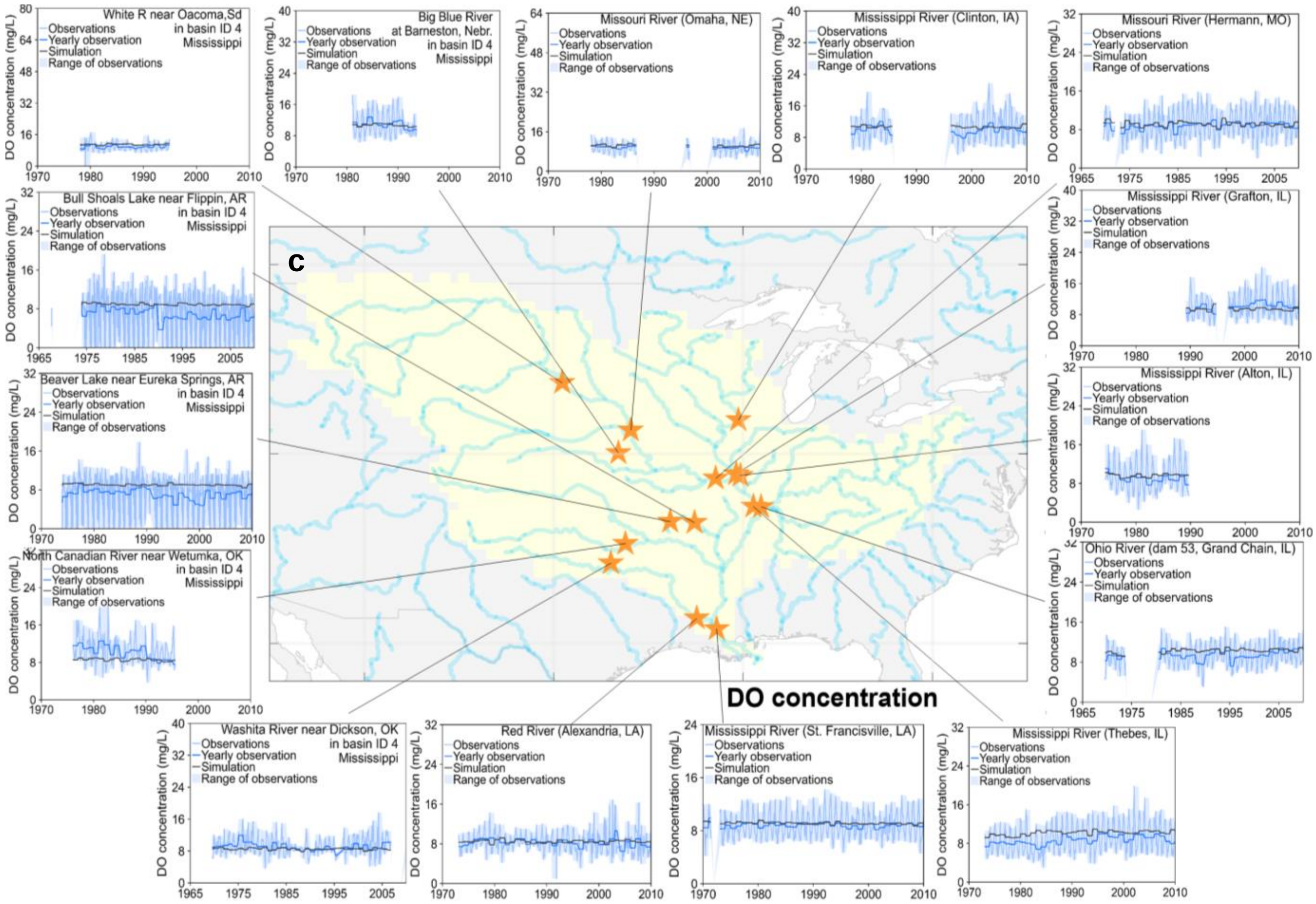

Figure S6. Validation of long-term temporal patterns of (a) water discharge, (b) TN concentrations, and (c) DO concentrations for different up-, mid- and downstream stations within the Mississippi River Basin since the 1930s, covering different waterbodies of streams, rivers, lakes and reservoirs. Observational data for validation are from the USGS database (USGS, 2022).

Figure S7. Validation: Distribution of sites of observational data from datasets and literature for validation of water discharge, TN, N_2O and DO in global inland waters since the 1920s.

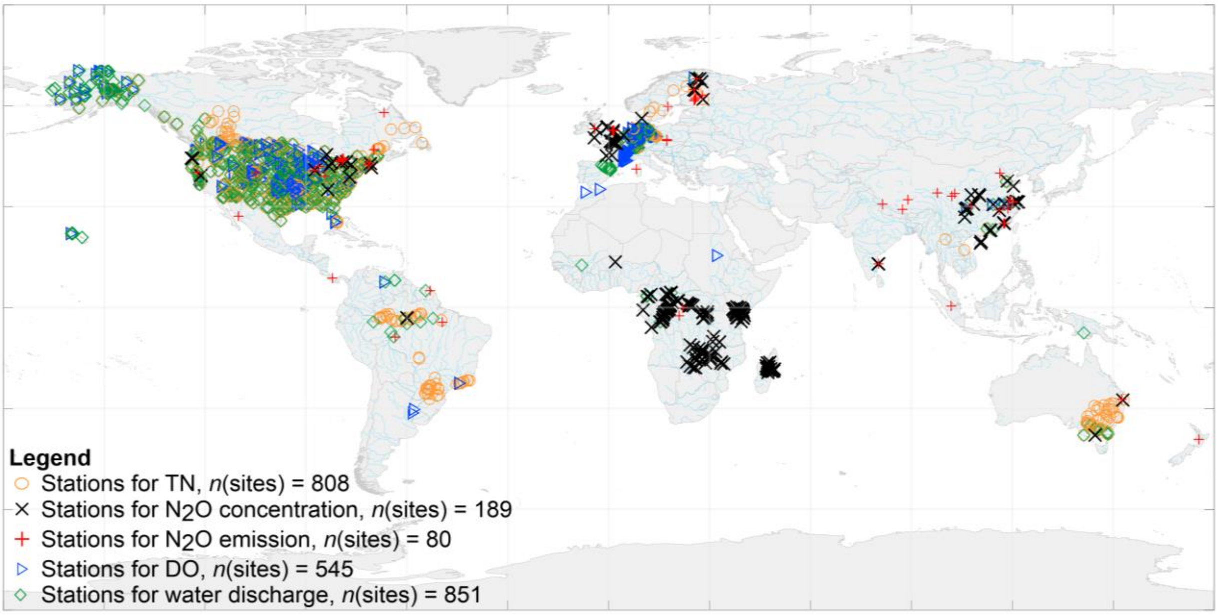

Figure S7. Distribution of sites of observational data from published datasets and literature for validation of water discharge, N_2O emission and concentration, and TN and DO concentrations per variable per site in global inland waters since the 1920s, covering different inland waterbodies, up-, mid- and downstream locations, and river basins in different climate zones.

Figure S8. Validation: RMSE of simulations and observations for water discharge, TN, N_2O and DO at monitoring sites in global inland waters for long time series since the 1920s.

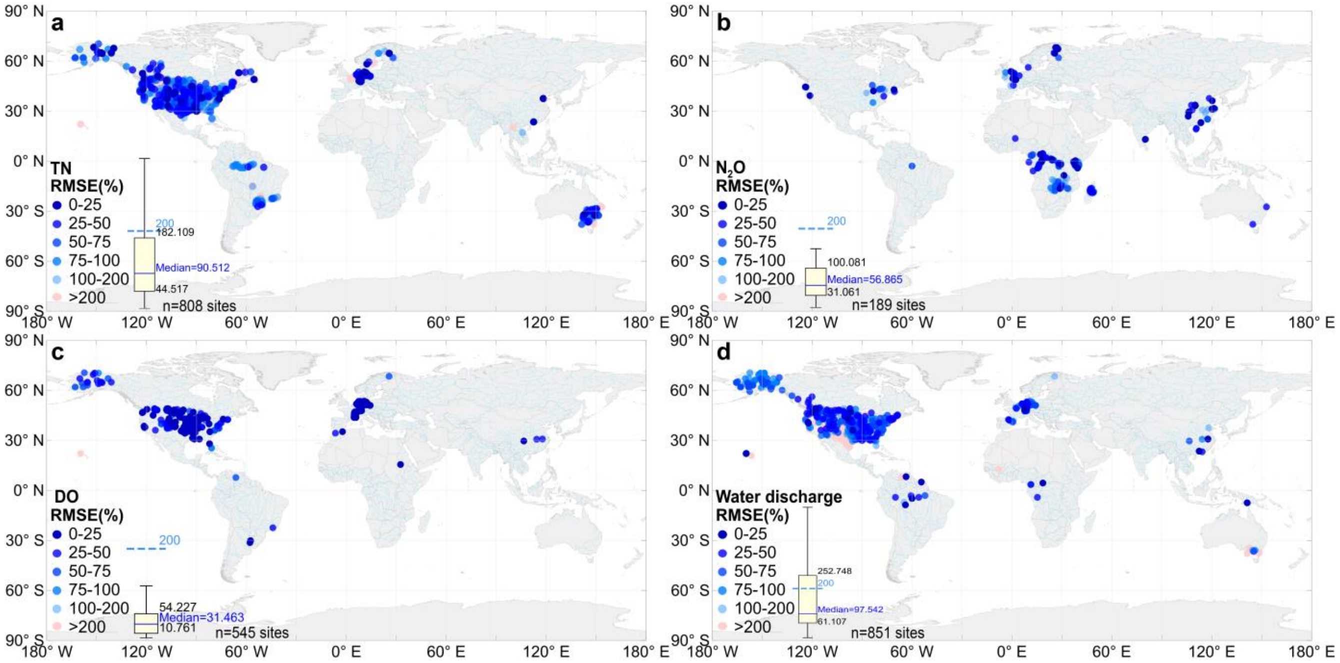

Figure S8. Root Mean Squared Error (RMSE) of the simulated and observed long time series of annual average concentrations of N_2O, TN, DO, and water discharge at monitoring sites in global inland waters for long time series since the 1920s: (a) TN, (b) N_2O, (c) DO, and (d) water discharge. The statistics of RMSE per variable for all stations worldwide are shown in its boxplot, with the median marked with dark blue color and RMSE=200% marked with light blue color. Information on the observational data used is in Table S3.

Figure S9. Validation: Comparison of simulated and observed N_2O concentrations per site per year in global inland waters since the 1970s.

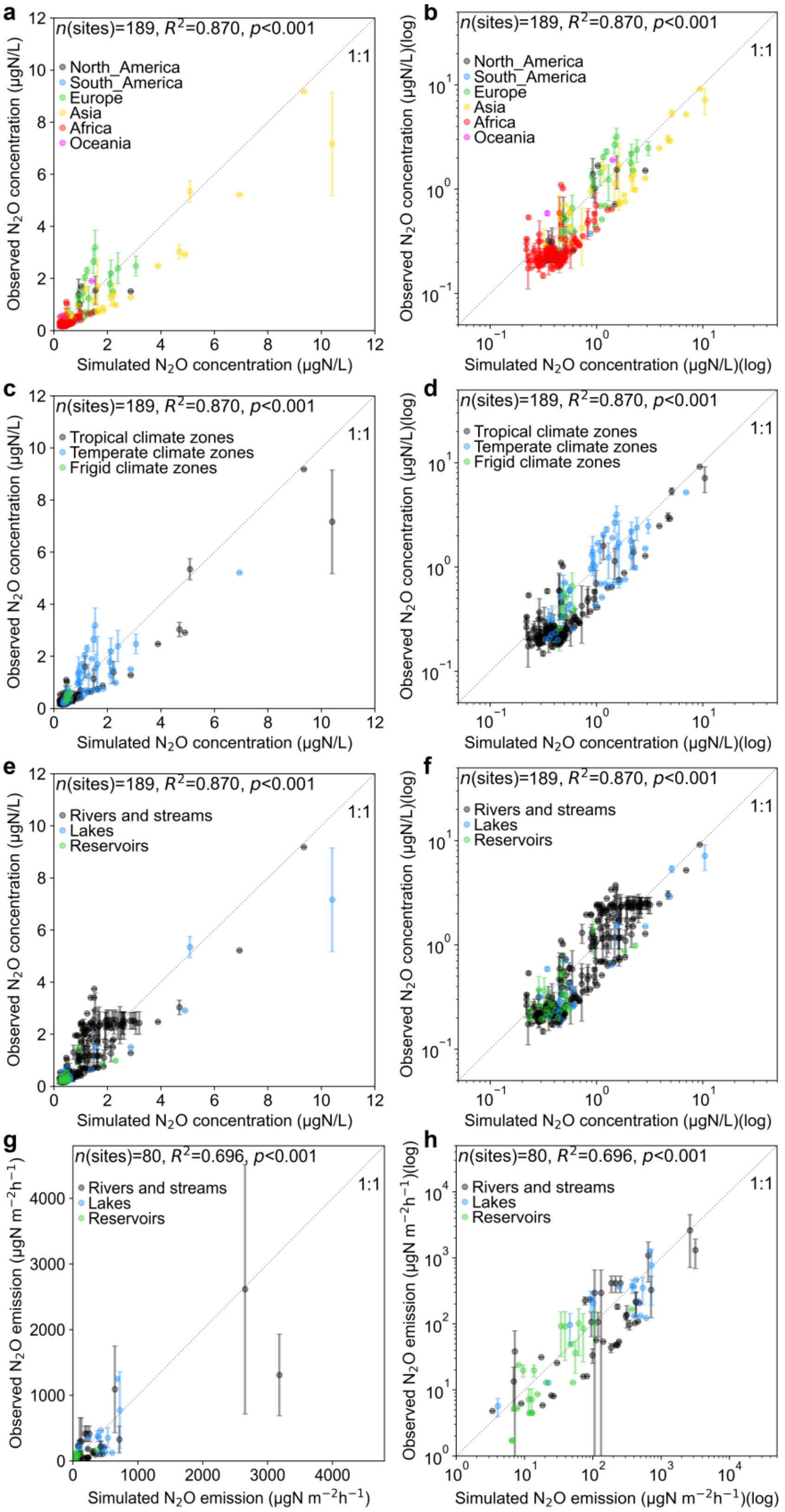

Figure S9. Comparison of simulated and observed N_2O concentrations per site per year in global inland waters since the 1970s in terms of different (a, b) continents, (c, d) climate zones, and (e, f) waterbodies, and comparison for N_2O emissions in different waterbodies (g, h). Figures on the left column (a, c, e, g) are presented on the 1:1 scale and those on the right column (b, d, f, h) are presented on the $\log_{10}$ scale. Error bars represent $\pm 1\sigma$. Information on the observational data used is in Table S3. The distribution of sites for observations is in Figure S7.

Figure S10. Sensitivity results for variations in model parameters, expressed as $\boldsymbol{Cx_i}$ (for Cx_i see Text S3)

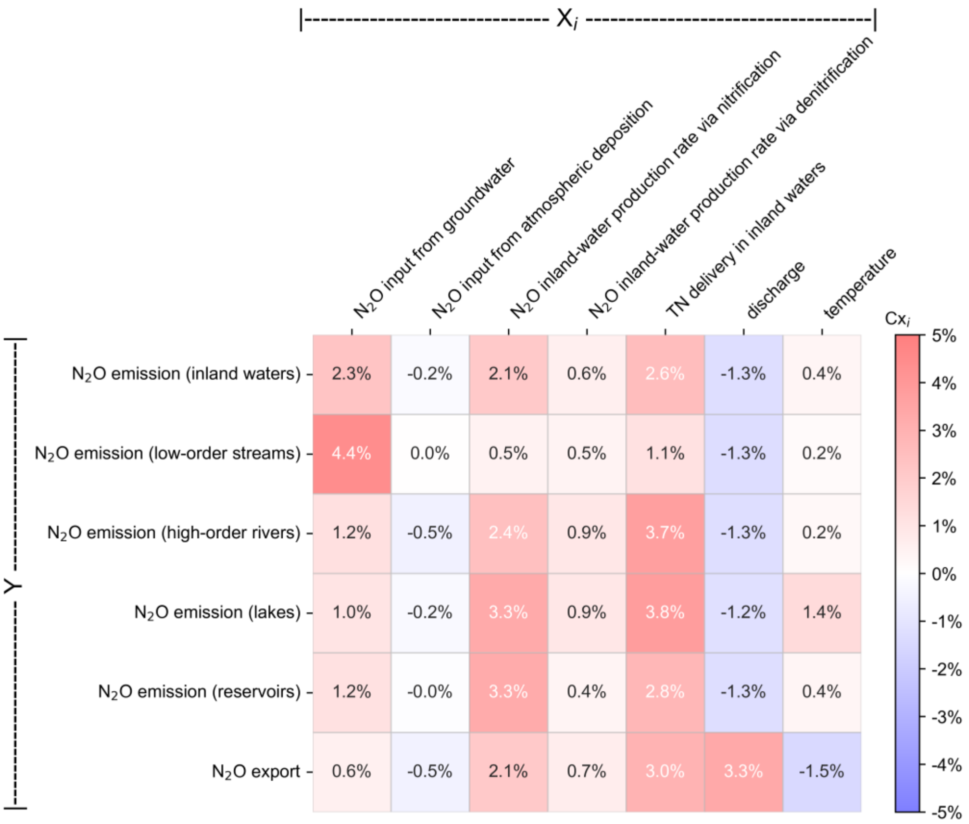

Figure S10. Sensitivity results of multi-year averages over the period 1995-2000 for variations in model parameters, expressed as Cx_i, i.e., the relative variation in output Y corresponding to the relative increase in an input X_i by 5%. Y (y-axis in the figure) includes N_2O emissions from total inland waters, low-order streams, high-order rivers, lakes and reservoirs, and dissolved N_2O export to oceans, and X_i (x-axis in the figure) includes N_2O input from groundwater, N_2O input from atmospheric deposition, N_2O inland-water production rate via nitrification, N_2O inland-water production rate via denitrification, TN (excluding N_2O) delivery in inland waters, discharge and temperature. Details about the method of sensitivity analysis including calculation of Cx_i are in Text S3.

Figure S11. Spatial distributions of N_2O inputs to the global river network in 1900 and 2010 simulated by IMAGE-DGNM

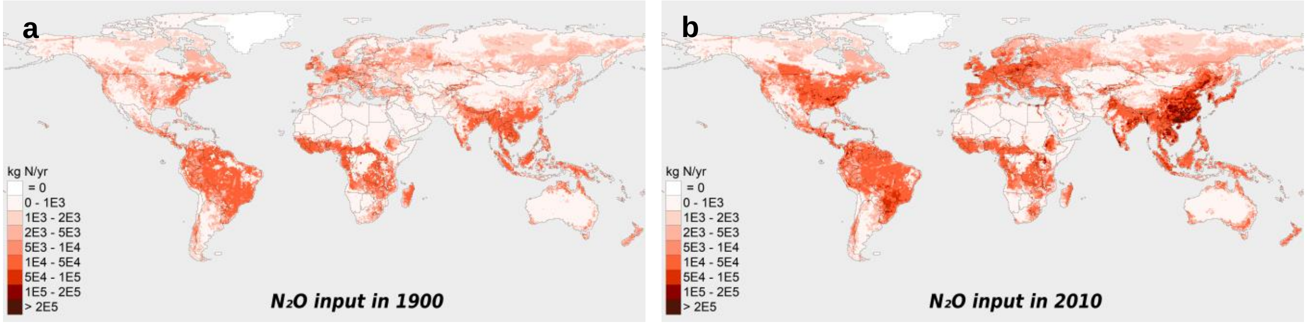

Figure S11. Spatial distribution of the total N_2O inputs (kg N yr^{-1} per 0.5°×0.5° grid) to the global river basins simulated by IMAGE-DGNM in (a) 1900 and (b) 2010.

Figure S12. Spatial distributions of N_2O production and multiple-form N loads in global inland waters in 1900 and 2010 simulated by IMAGE-DGNM

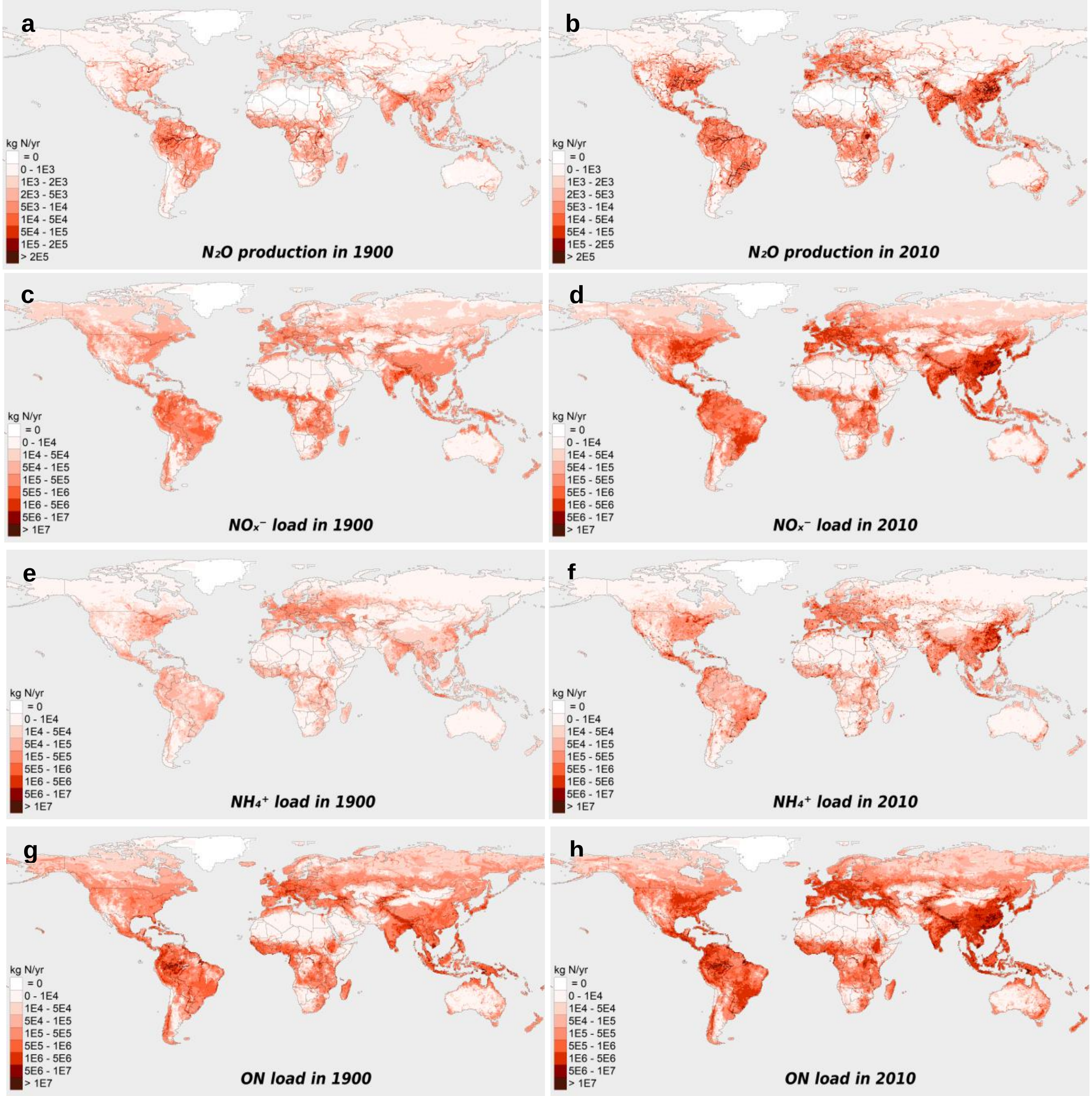

Figure S12. Spatial distribution of the total N_2O production (kg N yr^{-1} per 0.5°×0.5° grid) in global inland waters simulated by DISC-NITROGEN of IMAGE-DGNM in (a) 1900 and (b) 2010, and loads of different N forms to surface freshwaters in 1900 (c, e, g) and 2010 (d, f, i). Loads of different N forms are from Vilmin et al. (2018).

Figure S13. Temporal changes in the spatial distributions of the global freshwater annual N_2O emission during 1900-2010.

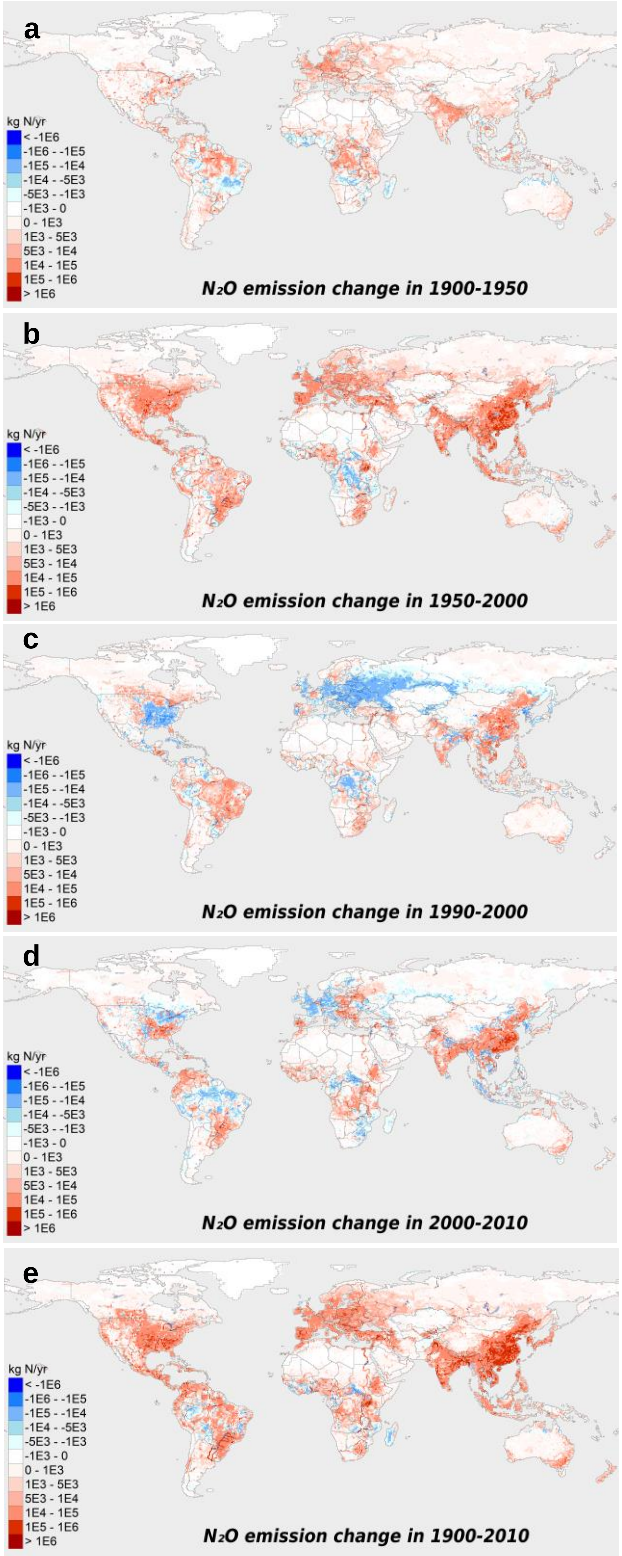

Figure S13. Temporal changes in the spatial distributions of the annual N_2O emission (kg N yr^{-1}) from global inland waters simulated by IMAGE-DGNM at a resolution of 0.5°×0.5° (a) between 1900 and 1950, (b) between 1950 and 2000, (c) between 1990 and 2000, (d) between 2000 and 2010, and (e) between 1900 and 2010.

Table S1. Methodology of inland-water N_2O dynamics in IMAGE-DGNM.

Processes	Descriptions and equations
**NIT** (nitrification in the column)	**Nitrification in the water column**, i.e., **oxidation of NH_4^+ to NO_x^-**, depends on ambient temperature, oxic condition (oxygen concentration), and the amount of reaction substrate NH_4^+.<br><br>**Temperature dependency:**<br>The inland-water temperature (T, in ℃) with a 0.5×0.5° resolution for each year during 1900-2010 is from the global mass-balanced hydrology model PCR-GLOBWB (Van Beek et al., 2011; Sutanudjaja et al., 2018), which simulates hydrological metrics with 5-arcmin resolution.<br>Temperature-dependency for nitrification in the water column is represented according to Billen et al. (1994) and Garnier et al. (2007), using the theoretical function of the process' optimal temperature ($T_{opt,NIT}$) and standard deviation (σ_{NIT}) as well as the local ambient water temperature for that time: $f_{NIT}(T) = e^{-\frac{(T-T_{opt,NIT})^2}{\sigma_{NIT}^2}}$<br><br>**Oxic-condition-dependency** (limiting factor by ambient **oxygen concentrations**):<br>The oxic and anoxic conditions are represented using the theoretical half-saturation oxygen concentration (Helder and de Vries, 1983; Cox, 2003) and the local ambient oxygen concentration simulated by the mass-balanced process-based model:<br>Limiting factor **for oxic conditions for nitrification**: $f_{O_2\_oxic\_NIT} = \frac{C_{O_2}}{K_{O_2,NIT}+C_{O_2}}$<br><br>**Nitrification:**<br>As was described in Vilmin et al. (2020), in IMAGE-DGNM, the equation of mass-balanced nitrification using the reaction stoichiometry of NO_x^- and O_2 and taking into account temperature dependency, oxic-condition-dependency and theoretical maximum nitrification rate (k_{nit}, from Thomann and Mueller (1987); (Cox, 2003)) is represented as:<br>$$\text{NIT} = f_{NIT}(T) \cdot f_{O_2\_oxic\_NIT} \cdot k_{nit} \cdot \min(NH_4^+, \frac{1}{2} O_2)$$<br>As the reaction substrate, NH_4^+ and O_2 can limit the nitrification process by both their absolute amounts and their ratio relative to the reaction stoichiometry.<br><br>**Production of N_2O during nitrification:**<br>While the above function in IMAGE-DGNM assumes that all nitrification in the water column transforms NH_4^+ to NO_x^-, this study focuses on N_2O fate by simulating both the oxidation of NH_4^+ to N_2O and the oxidation of NH_4^+ to NO_x^-, which depend on the above environmental conditions for nitrification and additional conditions for N_2O production. Based on Garnier et al. (2007), IMAGE-DGNM assumes a constant fraction ($f_{N_2O\_NIT}$=1%) of NH_4^+ converted to N_2O during the nitrification process.<br>**NO_x^- production via nitrification** in IMAGE-DGNM is represented as:<br>$$\text{NIT}_{NO_x^-} = (1 - f_{N_2O\_NIT}) \cdot f_{NIT}(T) \cdot f_{O_2\_oxic\_NIT} \cdot k_{nit} \cdot \min(NH_4^+, \frac{1}{2 - f_{N_2O\_NIT}} O_2)$$<br>**N_2O production via nitrification** in IMAGE-DGNM is represented as:<br>$$\text{NIT}_{N_2O} = \frac{1}{2} f_{N_2O\_NIT} \cdot f_{NIT}(T) \cdot f_{O_2\_oxic\_NIT} \cdot k_{nit} \cdot \min(NH_4^+, \frac{1}{2 - f_{N_2O\_NIT}} O_2)$$
**DENIT** (denitrification in the column)	**Denitrification in the water column**, i.e., **reduction of NO_x^- to N_2O and N_2, and reduction of N_2O to N_2**, depends on ambient temperature, anoxic condition (oxygen

concentration), availability of detrital organic matter, and concentrations of substrates (NO_x^- or N_2O).

Temperature dependency:
Temperature-dependency for denitrification in the water column is represented according to Billen et al. (1994) and Garnier et al. (2000), using the theoretical function of the process' optimal temperature ($T_{opt,DENIT}$) and standard deviation (σ_{DENIT}) as well as local water temperature for that time: $f_{DENIT}(T) = e^{-\frac{(T-T_{opt,DENIT})^2}{\sigma^2_{DENIT}}}$

Anoxia-dependency (limiting factor by ambient **oxygen concentrations**):
The oxic and anoxic conditions are represented using the theoretical half-saturation oxygen concentration (Garnier et al., 2007) and the local ambient oxygen concentration simulated by the mass-balanced process-based model:
Limiting factor for **anoxic conditions for denitrification**: $f_{O_2\_anoxic\_DENIT} = 1 - \frac{C_{O_2}}{K_{O_2,MIN}+C_{O_2}}$

Organic matter availability:
Only detrital (i.e., dead) organic matter can be utilized to provide energy for the denitrification process. The detrital organic matter in carbon is converted from detrital organic nitrogen (ON_{DET}) using the ratio of C:N = 106:16 according to Billen et al. (1994) and Garnier et al. (2000), as can be found in the full model description of the mass-balanced process-based IMAGE-DGNM model (Vilmin et al., 2020).

Denitrification:
As was described in Vilmin et al. (2020), in IMAGE-DGNM, the equation of mass-balanced denitrification using the reaction stoichiometry of organic matter and NO_x^- and taking into account temperature dependency, anoxia-dependency and theoretical maximum denitrification rate (k_{DENIT}, from Garnier et al. (2000)) is represented as:

$$DENIT = f_{\mathrm{DENIT}}(T) \cdot f_{O_2\_anoxic\_DENIT} \cdot k_{DENIT} \cdot \min\left(\frac{4}{5} \cdot \frac{106}{16} ON_{DET}, NO_x^-\right)$$

As reaction substrates, detrital organic matter and NO_x^- can limit denitrification by both their absolute amounts and their ratio relative to the reaction stoichiometry.

Incomplete denitrification (producing N_2O) vs. complete denitrification (producing N_2):
While the above function in IMAGE-DGNM assumes that all denitrification in the water column is complete, this study focuses on N_2O fate by simulating both the incomplete denitrification (transforming NO_x^- to N_2O) and complete denitrification (transforming NO_x^- and N_2O to N_2), which depend on the above environmental conditions for denitrification and additional conditions for N_2O production.
Based on Billen et al. (2020) and Beaulieu et al. (2011), IMAGE-DGNM assumes that a constant fraction ($f_{N_2O\_DENIT}$=1%) of NO_3^- used as electron acceptor for organic matter mineralization is converted to N_2O during the denitrification. Besides, according to Baulch et al. (2011), when the ambient temperature is not lower than the temperature threshold $T_{lim,N2O\ reduc}$, N_2O production via incomplete denitrification can occur; when the ambient temperature is lower than $T_{lim,N2O\ reduc}$, N_2O produced via denitrification cannot be preserved and will be fully denitrified, i.e., only complete denitrification occurs. In addition, according to Baulch et al. (2011), if NO_x^- concentration is lower than the threshold $K_{NO3,N2O\ reduc}$, consumption of inland-water N_2O via complete denitrification will also occur, i.e., using N_2O as the electron

acceptor for organic matter mineralization and transforming N_2O to N_2.

N_2O production via incomplete denitrification in IMAGE-DGNM is represented as:

$$\mathrm{DENIT}_{N_2O} = \frac{1}{2} f_{N_2O\_DENIT} \cdot f_{\mathrm{DENIT}}(T) \cdot f_{O_2\_anoxic\_DENIT} \cdot k_{DENIT} \cdot \min\left(\frac{4}{5} \cdot \frac{106}{16} ON_{DET},\ NO_x^-\right) \quad if\ T \geq T_{lim,N_2O\ reduc}$$

N_2 production via complete denitrification in IMAGE-DGNM is represented as:

· NO_x^- to N_2:

$$\mathrm{DENIT}_{N_2(NO_x^-)} = \frac{1}{2} f_{\mathrm{DENIT}}(T) \cdot f_{O_2\_anoxic\_DENIT} \cdot k_{DENIT} \cdot \min\left(\frac{4}{5} \cdot \frac{106}{16} ON_{DET},\ NO_x^-\right) \quad if\ T < T_{lim,N_2O\ reduc}$$

$$\mathrm{DENIT}_{N_2(NO_x^-)} = \frac{1}{2}\left(1 - f_{N_2O\_DENIT}\right) \cdot f_{\mathrm{DENIT}}(T) \cdot f_{O_2\_anoxic\_DENIT} \cdot k_{DENIT} \cdot \min\left(\frac{4}{5} \cdot \frac{106}{16} ON_{DET},\ NO_x^-\right) \quad if\ T \geq T_{lim,N_2O\ reduc}$$

· N_2O to N_2, i.e., N_2O consumption:

N_2O reduction limiting factor for NO_x^- concentration using the threshold $K_{NO3,N2O\ reduc}$ (Baulch et al., 2011):

$$f_{NO_3,N_2O\ reduc} = 1 - \frac{1}{1 + e^{-\left(C_{NO_3^-} - K_{NO_3,N2O\ reduc}\right)}}$$

$$\mathrm{DENIT}_{N_2(N_2O)} = f_{NO_3,N_2O\ reduc} \cdot f_{\mathrm{DENIT}}(T) \cdot f_{O_2\_anoxic\_DENIT} \cdot k_{DENIT} \cdot \min\left(\frac{2}{1} \cdot \frac{106}{16} ON_{DET},\ N_2O\right)$$

Nitrifier denitrification (NIT_DENIT) in IMAGE-DGNM depends on the environmental conditions for nitrification, the amount of nitrification substrate NH_4^+, as well as the theoretical half-saturation oxygen concentration, the theoretical half-saturation NO_x^- concentration, the threshold of maximum oxygen concentration for nitrifier denitrification and the concentrations of reaction substrates oxygen and NO_x^-.

Limiting factor for **oxygen conditions** using the theoretical half-saturation oxygen concentration and the threshold of maximum oxygen concentration **for nitrifier denitrification**: $f_{O_2\_NIT\_DENIT} = \frac{C_{O_2}}{k_{O_2,NIT\_DENIT} + C_{O_2}} \cdot e^{-\left(\frac{C_{O_2}}{S_{O_2,NIT\_DENIT}}\right)^2}$

Limiting factor for **NO_x^- concentrations** using the theoretical half-saturation NO_x^- concentration for **nitrifier denitrification**: $f_{NO_x\_NIT\_DENIT} = \frac{C_{NO_x^-}}{k_{NO_x,NIT\_DENIT} + C_{NO_x^-}}$

$$NIT\_DENIT = f_{NIT}(T) \cdot f_{O_2\_NIT\_DENIT} \cdot f_{NO_x\_NIT\_DENIT} \cdot k_{nit\_denit} \cdot \min\left(NH_4^+,\ \frac{4}{3} O_2\right)$$

N_2O production via nitrifier denitrification:

While the above function in IMAGE-DGNM assumes that all nitrifier denitrification in the water column is complete, this study focuses on N_2O fate by simulating both the incomplete (transforming NO_x^- to N_2O) and complete nitrifier denitrification (transforming NO_x^- to N_2), which depend on the above environmental conditions for nitrifier denitrification and additional conditions for N_2O production. Based on Garnier et al. (2007), we assume a constant fraction ($f_{N_2O\_NIT\_DENIT}$=1%) of NH_4^+ converted to N_2O during the nitrifier denitrification processes NIT_DENIT when the oxygen concentration is lower than the threshold S_{O2,NIT_DENIT} (Garnier et al., 2007).

N_2O production via incomplete nitrifier denitrification in IMAGE-DGNM is represented as:

$$\text{NIT\_DENIT}_{N_2O} = \frac{1}{2} f_{N_2O\_NIT\_DENIT} \cdot f_{NIT}(T) \cdot f_{O_2\_NIT\_DENIT} \cdot f_{NO_x\_NIT\_DENIT} \cdot k_{nit\_denit} \cdot \min\left(NH_4^+, \frac{4}{3+f_{N_2O\_NIT\_DENIT}} O_2\right)$$

N_2 production via complete nitrifier denitrification in IMAGE-DGNM is represented as:

$$\text{NIT\_DENIT}_{N_2} = \frac{1}{2}(1 - f_{N_2O\_NIT\_DENIT}) \cdot f_{NIT}(T) \cdot f_{O_2\_NIT\_DENIT} \cdot f_{NO_x\_NIT\_DENIT} \cdot k_{nit\_denit} \cdot \min\left(NH_4^+, \frac{4}{3+f_{N_2O\_NIT\_DENIT}} O_2\right)$$

BNIT (nitrification in sediments)

Nitrification in sediments, i.e., **NH_4^+ uptake from the water column due to nitrification in the benthic layer**, depends on the depth of the uncompacted sediment layer (Z_f, in m), total bed area (A, in m^2), temperature, oxic condition (oxygen concentration), and the concentration of reaction substrate NH_4^+, according to Billen et al. (2015).

Temperature dependency:

Temperature-dependency for nitrification in sediments is represented according to Billen et al. (2015), using the theoretical function of the process' optimal temperature ($T_{opt,BNIT}$) and standard deviation (σ_{BNIT}) as well as the local temperature for that time:

$$f_{BNIT}(T) = e^{-\frac{\left(T - T_{opt,BNIT}\right)^2}{\sigma_{BNIT}^2}}$$

As was described in Vilmin et al. (2020), in IMAGE-DGNM, the equation of mass-balanced nitrification in sediments using the reaction stoichiometry of NO_x^- and oxygen and taking into account temperature dependency, oxic-condition-dependency, the depth of the uncompacted sediment layer (Z_f), and total bed area (A) is represented as:

$$\text{BNIT} = f_{\text{BNIT}}(T) \cdot \frac{C_{O_2}}{C_{O_2,sat}} \cdot A \cdot \left(0.015 \cdot z_f + 0.00125 \cdot \frac{z_f}{z_f + 0.002} \cdot \min\left(C_{NH_4^+}, \frac{1}{2} C_{O_2}\right)\right)$$

As reaction substrates, NH_4^+ and O_2 can limit the benthic nitrification process by both their absolute amounts and their ratio relative to the reaction stoichiometry. $C_{O_2,sat}$ is calculated using temperature according to APHA (1992).

Production of N_2O during nitrification in sediments:

While the above function in IMAGE-DGNM assumes that all nitrification in sediments transforms NH_4^+ to NO_x^-, this study focuses on N_2O fate by simulating both the oxidation of NH_4^+ to N_2O and the oxidation of NH_4^+ to NO_x^-, which depend on the above environmental conditions for benthic nitrification and additional conditions for N_2O production. Based on Garnier et al. (2007), IMAGE-DGNM assumes a constant fraction ($f_{N_2O\_BNIT}$=1%) of NH_4^+ converted to N_2O during the nitrification process.

NO_x^- production via nitrification in sediments in IMAGE-DGNM is represented as:

$$\text{BNIT}_{NO_x^-} = (1 - f_{N_2O\_BNIT}) \cdot f_{\text{BNIT}}(T) \cdot \frac{C_{O_2}}{C_{O_2,sat}} \cdot A \cdot \left(0.015 \cdot z_f + 0.00125 \cdot \frac{z_f}{z_f + 0.002} \cdot \min\left(C_{NH_4^+}, \frac{1}{2 - f_{N_2O\_BNIT}} C_{O_2}\right)\right)$$

N_2O production via nitrification in sediments in IMAGE-DGNM is represented as:

$$\text{BNIT}_{N_2O} = \frac{1}{2} f_{N_2O\_BNIT} \cdot f_{\text{BNIT}}(T) \cdot \frac{C_{O_2}}{C_{O_2,sat}} \cdot A \cdot \left(0.015 \cdot z_f + 0.00125 \cdot \frac{z_f}{z_f + 0.002} \cdot \min\left(C_{NH_4^+}, \frac{1}{2 - f_{N_2O\_BNIT}} C_{O_2}\right)\right)$$

BDENIT (denitrification

Denitrification in sediments, i.e., **reduction of NO_x^- to N_2O and N_2**, depends on temperature, anoxic condition (oxygen concentration), availability of detrital organic

in sediments)	matter, and concentrations of substrate NO_x^-.<br><br>**Temperature dependency:**<br>Temperature-dependency for denitrification in the water column is represented according to Billen et al. (2015), using the theoretical function of the process' optimal temperature ($T_{opt,DENIT}$) and standard deviation (σ_{DENIT}) as well as the local ambient water temperature for that time: $f_{BDENIT}(T) = e^{-\frac{(T-T_{opt,BDENIT})^2}{\sigma^2_{BDENIT}}}$<br><br>**Organic matter availability**:<br>Only detrital (i.e., dead) organic matter in sediments can be utilized to provide energy for the benthic denitrification process. The detrital organic matter in carbon is converted from detrital organic nitrogen ($ON_{DET,b}$) using the ratio of C:N = 106:16 according to Billen et al. (2015), as can be found in the full model description of the mass-balanced process-based IMAGE-DGNM model (Vilmin et al., 2020). According to Billen et al. (2015), the integrated carbon oxidant demand of organic matter degradation (Coxd, in equ. $m^{-2}\cdot h^{-1}$) for benthic denitrification depends on amounts of benthic organic matter ($ON_{DET,b}$) and sediments (TBS), maximum compaction rate (C_{max}) and maximum mineralization rate (k_{BMIN}). Coxd (Billen et al., 2015) is thus represented as: Coxd $= \frac{4}{12} \cdot \left(k_{BMIN} \cdot ON_{DET,b} + C_{max} \cdot (TBS - TBS_{lim}) \cdot ON_{DET,b}\right)$<br><br>**Anoxia-dependency** (limiting factor by **oxygen concentrations**):<br>The oxic and anoxic conditions are represented according to Billen et al. (2015) using the relative ratio of the local oxygen and NO_x^- concentrations:<br>Limiting factor for **anoxic conditions for benthic denitrification**:<br>$f_{O_2\_anoxic\_BDENIT} = 2 \cdot \frac{\frac{C_{NO_3^-}}{C_{O_2}}}{\frac{C_{NO_3^-}}{C_{O_2}}+1.8}$<br><br>**Denitrification in sediments:**<br>As was described in Vilmin et al. (2020), in IMAGE-DGNM, the equation of mass-balanced denitrification in sediments using the reaction stoichiometry of benthic organic matter and NO_x^- and taking into account temperature dependency, anoxia-dependency, depth of the uncompacted sediment layer (Z_f), and theoretical maximum denitrification rate (k_{BDENIT}, from Billen et al. (2015)) is represented as:<br>$BDENIT = f_{\text{BDENIT}}(T) \cdot f_{O_2\_anoxic\_BDENIT} \cdot k_{BDENIT} \cdot \frac{1-Coxd^{0.7}}{Coxd^{0.7}+\left(C_{NO_3^-} \cdot \frac{0.0005}{Z_f+0.0005}\right)^{0.7}} \cdot \min\left(\frac{4}{5} \cdot \frac{106}{16} ON_{DET,b}, NO_x^-\right)$<br>As the reaction substrates, benthic detrital organic matter and NO_x^- can limit the benthic denitrification process by both their absolute amounts and their ratio relative to the reaction stoichiometry.<br><br>**Incomplete denitrification (producing N_2O) vs. complete denitrification (producing N_2):**<br>While the above function in IMAGE-DGNM assumes that all sedimentary denitrification is complete, this study focuses on N_2O fate by simulating both the incomplete (transforming NO_x^- to N_2O) and complete denitrification in sediments (transforming NO_x to N_2), which depend on the above environmental conditions for benthic denitrification and additional conditions for N_2O production. Based on Billen et al. (2020) and Beaulieu et al. (2011), IMAGE-DGNM assumes that a constant

	fraction ($f_{N_2O\_BDENIT}$=1%) of NO_3^- used as electron acceptor for benthic organic matter mineralization is converted to N_2O during denitrification in sediments.<br><br>**N_2O production via incomplete sedimentary denitrification** in IMAGE-DGNM is represented as:<br>$\mathrm{BDENIT}_{N_2O} = \frac{1}{2} f_{N_2O\_BDENIT} \cdot f_{\mathrm{BDENIT}}(T) \cdot f_{O_{2}\_anoxic\_BDENIT} \cdot k_{BDENIT} \cdot \frac{1-Coxd^{0.7}}{Coxd^{0.7}+\left(C_{NO_3^-} \cdot \frac{0.0005}{Z_f+0.0005}\right)^{0.7}} \cdot \min\left(\frac{4}{5} \cdot \frac{106}{16} ON_{DET,b}, NO_x^-\right)$<br><br>**$N_2$ production via complete sedimentary denitrification** in IMAGE-DGNM is represented as:<br>$\mathrm{BDENIT}_{N_2} = \frac{1}{2}(1 - f_{N_2O\_BDENIT}) \cdot f_{\mathrm{BDENIT}}(T) \cdot f_{O_{2}\_anoxic\_BDENIT} \cdot k_{BDENIT} \cdot \frac{1-Coxd^{0.7}}{Coxd^{0.7}+\left(C_{NO_3^-} \cdot \frac{0.0005}{Z_f+0.0005}\right)^{0.7}} \cdot \min\left(\frac{4}{5} \cdot \frac{106}{16} ON_{DET,b}, NO_x^-\right)$
**$EXCH_{N_2O}$** (inland-water N_2O exchange with the atmosphere)	**N_2O exchange at the water-atmosphere interface** can be positive if N_2O is emitted from the water to the atmosphere, or negative if N_2O from the atmosphere is taken up by inland waters. According to Alin et al. (2011), N_2O exchange at the water-atmosphere interface depends on the temperature-dependent N_2O saturation concentration ($C_{N_2O,sat}(T)$, in mol·m^{-3}), gas transfer velocity (k_a, in m·h^{-1}), waterbody volume (V, in m^3) and local inland-water N_2O concentration.<br><br>**Gas transfer velocity k_a** at the freshwater-atmosphere interface is calculated according to Alin et al. (2011) from the gas transfer velocity at 20°C (k_{600}) and Schmidt value for N_2O in freshwater at temperature T (Sc(T)) as: $k_a = k_{600} \cdot \left(\frac{\mathrm{Sc(T)}}{600}\right)^{-0.5}$<br><br>**$k_{600}$** is calculated using (1) flow velocity (U, in m·s^{-1}) for streams with width<100 m: $\mathrm{k}_{600} = \frac{13.82+0.35 \cdot \mathrm{U} \cdot 0.01}{100}$, (2) constant wind speed of 10 km·h^{-1} for rivers with width ≥ 100 m: k_{600} = 0.2421, and (3) method and maximum for lakes and reservoirs from Raymond et al. (2013).<br><br>**The temperature-dependent Sc(T) for N_2O** is represented according to Wanninkhof (1992) as: $\mathrm{Sc(T)} = 2055.6 - 137.11 \cdot \mathrm{T} + 4.3173 \cdot \mathrm{T}^2 - 0.05435 \cdot \mathrm{T}^3$<br><br>**The N_2O saturation concentration $C_{N_2O,sat}(T)$ for each location and time point** is represented using temperature and the mole fraction of atmospheric N_2O in dry air (pN_2O, data in Figure S1) according to Weiss and Price (1980):<br>$C_{N_2O,sat}(T) = 1000 \cdot pN_2O \cdot \exp\left(-165.8806 + \frac{222.8743 \cdot 100}{\mathrm{T}+273.15} + 92.0792 \cdot \ln\left(\frac{\mathrm{T}+273.15}{100}\right) - 1.48425 \cdot \left(\frac{\mathrm{T}+273.15}{100}\right)^2\right)$<br><br>**$N_2O$ exchange at the water-atmosphere interface** in IMAGE-DGNM is represented as:<br>$EXCH_{N_2O} = k_a \cdot (C_{N_2O,sat} - C_{N_2O}) \cdot V$

Note: Process rates are expressed in mol·h^{-1}. For species *i*, the amount of *i* is expressed as X_i (e.g., ON_{pp}) in mol, C_i is the concentration of *i* in the water column in mol·m^{-3}. The units, values, and sources of the mentioned parameters are in Table S2. For the equations, stoichiometry, parameters, units and descriptions of other N processes in IMAGE-DGNM, see the full model description in Vilmin et al. (2020).

Table S2a. Sources of model inputs of IMAGE-DGNM

Model inputs	Data source
Surface water area, volume, precipitation, runoff, and discharge in rivers, lakes, and reservoirs	PCR-GLOBWB (Van Beek et al., 2011; Sutanudjaja et al., 2018)
Lake area	Global Lakes and Wetlands Database (GLWD1) (Lehner and Döll, 2004)
Reservoir area	Global Reservoir and Dam database (GRanD v1.3) (Lehner et al., 2011)
Temperature, population, land cover, water use	IMAGE (Stehfest et al., 2014)
Inputs of ON, NO_x^-, NH_4^+, DO and TSS to surface waters	IMAGE-DGNM (Vilmin et al., 2020)

Table S2b. Parameters used to simulate inland-water N_2O dynamics in IMAGE-DGNM.

Parameter	Description	Unit	Value	Source
$T_{opt,DENIT}$	Optimal temperature for denitrification in water column	°C	25	(Garnier et al., 2000)
σ_{DENIT}	Standard deviation of temperature-dependency function for denitrification	°C	15	(Garnier et al., 2000)
k_{DENIT}, k_{MIN}	Maximum denitrification rate in water column and organic matter mineralization rate	h^{-1}	0.002*	(Garnier et al., 2000)
$k_{O2,MIN}$	O_2 half-saturation concentration for oxic organic matter mineralization in water column	$mol \cdot m^{-3}$	0.015625	(Garnier et al., 2007)
$T_{lim,N2O\ reduc}$	The lowest temperature for N_2O reduction to N_2	°C	3.1	(Baulch et al., 2011)
$K_{NO3,N2O\ reduc}$	The highest NO_3^- concentration for N_2O reduction to N_2	$mol \cdot m^{-3}$	0.0027	(Baulch et al., 2011)
$T_{opt;NIT}$, $T_{opt;NIT\_DENIT}$	Optimal temperature for nitrification (or nitrifier denitrification)	°C	23	(Garnier et al., 2007)
σ_{NIT}, σ_{NIT_DENIT}	Standard deviation of temperature-dependency function for nitrification (or nitrifier denitrification)	°C	12	(Garnier et al., 2007)
k_{nit}, k_{nit_denit}	Maximum nitrification (or nitrifier denitrification) rate in water column	h^{-1}	0.5	(Thomann and Mueller, 1987; Cox, 2003)
$k_{O2,NIT}$	O_2 half-saturation concentration for nitrification in water column	$mol \cdot m^{-3}$	0.046875	(Helder and de Vries, 1983; Cox, 2003)
k_{O2,NIT_DENIT}	O_2 half-saturation concentration for nitrifier denitrification	$mol \cdot m^{-3}$	0.01875	(Garnier et al., 2007)
k_{NOx,NIT_DENIT}	NO_x half-saturation concentration for nitrifier denitrification	$mol \cdot m^{-3}$	0.07143	(Garnier et al., 2007)
S_{O2,NIT_DENIT}	The highest O_2 concentration for nitrifier denitrification	$mol \cdot m^{-3}$	0.046875	(Garnier et al., 2007)
$T_{opt,BDENIT}$	Optimal temperature for benthic denitrification	°C	20	(Billen et al., 2015)
σ_{BDENIT}	Standard deviation of temperature-dependency function for benthic denitrification	°C	17	(Billen et al., 2015)
k_{BDENIT}, k_{BMIN}	First-order benthic organic matter mineralization rate and denitrification rate	h^{-1}	0.002*	(Billen et al., 2015)
C_{max}	Maximal compaction rate	h^{-1}	0.0005	(Billen et al., 2015)
$T_{opt,BNIT}$	Optimal temperature for benthic nitrification	°C	20	(Billen et al., 2015)
σ_{BNIT}	Standard deviation of temperature-dependency function for benthic nitrification	°C	17	(Billen et al., 2015)

Note: * assuming 40, 40 and 20% of highly biodegradable, moderately biodegradable and refractory organic matter, respectively. For descriptions of other N processes in IMAGE-DGNM, see the full model description in Vilmin et al. (2020).

Table S3. Summary of observation data from databases and literature used for comparison with model outputs of TN and DO concentrations, N_2O concentration and emission, and water discharge per variable per site per river basin in global inland waters for the period since the 1920s, covering different waterbodies, up-, mid- and downstream sites and river basins in different climate zones. The spatial distribution of all observational sites used for validation is in Figure S7. Validation results are in Figures S8-S9.

Variable	Description	Period covered	Waterbodies covered	Climate zones covered	Continents covered	Data source
**TN concentration**	Total nitrogen, NO_x^- + NH_4^+ + ON	1967-2010	River mainstreams, headwaters, lakes, reservoirs	Tropical, temperate, frigid	North America, South America, Europe, Asia, Oceania	(Hartmann et al., 2019; Rijkswaterstaat, 2022; USGS, 2022)
**N_2O concentration and emission**	Dissolved nitrous oxide concentration, and nitrous oxide emission	1977-2010 (observations during 2011-2019 are compared with simulations in 2010)	River mainstreams, headwaters, lakes, reservoirs	Tropical, temperate, frigid	North America, South America, Europe, Asia, Africa, Oceania	(Hahn and Junge, 1977; Kaplan et al., 1978; McElroy et al., 1978; Dowdell et al., 1979; Lemon and Lemon, 1981; de Angelis and Gordon, 1985; Davidson and Firestone, 1988; Richey et al., 1988; Hemond and Duran, 1989; Davidson and Swank, 1990; Bange et al., 1996; Mengis et al., 1996; Paludan and Blicher-Mathiesen, 1996; Robinson et al., 1998; McMahon and Dennehy, 1999; De Wilde and De Bie, 2000; Marty et al., 2001; Amouroux et al., 2002; De Bie et al., 2002; Dong et al., 2002; Huttunen et al., 2002a; Huttunen et al., 2002b; Lima et al., 2002; Harrison and Matson, 2003; Hiscock et al., 2003; Huttunen et al., 2003; LaMontagne et al., 2003; Dong et al., 2004; Huttunen et al., 2004; Laursen and Seitzinger, 2004; Yan et al., 2004; Hendzel et al., 2005; Xu et al., 2005; Garnier et al., 2006; Hlaváčová et al., 2006; Wang et al., 2006; Zhang et al., 2006; Ferrón et al., 2007; Beaulieu et al., 2008; Chen et al., 2008; Guérin et al., 2008; Nirmal Rajkumar et al., 2008; Silvennoinen et al., 2008; Wilcock and Sorrell, 2008; Zhang et al., 2008; Wang et al., 2009; Zhao et al., 2009a; Zhao et al., 2009b; Beaulieu et al., 2010; Li, 2010; Zhang et al., 2010; Baulch et al., 2011; Chen et al., 2011; Liu et al., 2011; Baulch et al., 2012; Jauhiainen and Silvennoinen, 2012; Outram and Hiscock, 2012; Rosamond et al., 2012; Hinshaw and Dahlgren, 2013; Wong et al., 2013; Beaulieu et al., 2014; Chen et al., 2014; Musenze et al., 2014;

						Sturm et al., 2014; Borges et al., 2015; Chen et al., 2015a; Chen et al., 2015b; Zhu et al., 2015; Mach et al., 2016; Schade et al., 2016; Soued et al., 2016; Wang et al., 2016; Audet et al., 2017; Hama-Aziz et al., 2017; He et al., 2017; Upstill-Goddard et al., 2017; Wang et al., 2017a; Wang et al., 2017b; Davis and David, 2018; Yan et al., 2018; Borges et al., 2019; Smith and Böhlke, 2019; Xiao et al., 2019a; Xiao et al., 2019b; Yang et al., 2019; Zhou et al., 2019; Kortelainen et al., 2020; Wang et al., 2020; Zhao and Zhang, 2021; Barthel et al., 2022; Wang et al., 2022)
**DO concentration**	Dissolved oxygen	1952-2010	River mainstreams, headwaters, lakes, reservoirs	Tropical, temperate, frigid	North America, South America, Europe, Asia, Africa	(Hartmann et al., 2019; Rijkswaterstaat, 2022; USGS, 2022)
**Discharge**	Water discharge	1925-2010	River mainstreams, headwaters, lakes, reservoirs	Tropical, temperate, frigid	North America, South America, Europe, Asia, Africa, Oceania	(Ministry_of_Water_Resources_of_the_People's_Republic_of_China, 2003; Hartmann et al., 2019; Rijkswaterstaat, 2022; USGS, 2022)

Table S4. Comparison with reported estimates of global N_2O emissions from inland waters.

Covering period	Spatial resolution	Temporal resolution	Method+ Reference	Time	Waterbody type	N_2O emission ($Tg\ N\ yr^{-1}$)
1988-1990	-	-	Upscaling (Law et al., 1992)	1988-1990	Estuaries	0.22
1991-1992	-	-	Upscaling (Bange et al., 1996)	1991-1992	Estuaries	3.7-5.7
1994-1995	-	-	Upscaling (Robinson et al., 1998)	1994-1995	Estuaries	0.13-0.45
1993-1996	-	-	Upscaling (De Wilde and De Bie, 2000)	1993-1993	Estuaries+coastal zones	1.5
1980s-2010s	-	-	Upscaling (Murray et al., 2015)	1980s-2010s	Estuaries	0.23 (0.13-0.44)
					Estuaries+coastal zones	0.31 (0.15-0.91)
1990	1°×1°	-	N-Model (Seitzinger and Kroeze, 1998; Seitzinger et al., 2000)	1990	Rivers	1.05
					Estuaries	0.22
1989	-	-	Emission factor (IPCC, 1996; Mosier et al., 1998; Kroeze et al., 1999)	1989	Rivers	0.5
					Rivers(+groundwater)	1.6
1995	0.5°×0.5°	-	NEWS-DIN (Kroeze et al., 2005; IPCC, 2007)	1995	Rivers	1.256
					Estuaries	0.251
1970, 2000	0.5°×0.5°	-	NEWS (Kroeze et al., 2010)	1970	Rivers	0.45
					Estuaries	0.15
				2000	Rivers	0.3-1.6
					Estuaries	0.1-0.6
1990s	-	-	AR4, emission factor (IPCC, 2013)	1990s	Rivers+estuaries	1.7 (0.5-2.9)
1990, 2000	-	-	Emission factor (Syakila and Kroeze, 2011)	1990	Rivers+estuaries	1.1
				2000	Rivers+estuaries	1.1
Mid-1990s, 2006-2011	-	-	AR5, emission factor (IPCC, 2013)	1995	Rivers+estuaries	0.6 (0.1-2.9)
				2006-2011	Rivers+estuaries	0.6 (0.1-2.9)
Mid-1990s	0.5°×0.5°	-	FrAMES-N + upscaling(Beaulieu et al., 2011)	Mid-1990s	Rivers+streams	0.68
2000s	Basin scale	-	NEWS2-DIN-S + upscaling (Hu et al., 2016)	2000s	Rivers	0.03 (0.01-0.07)
1990-2010	-	-	Upscaling (Deemer et al., 2016)	1990-2010	Reservoirs	0.03
1970s-2010s	-	-	Upscaling (Soued et al., 2016)	1970s-2010s	Inland waters	0.82
					Rivers+streams	0.19
					Lakes+reservoirs	0.63
2000	0.5°×0.5°	-	Static mechanistic LOAC model+ NEWS2 (Maavara et al., 2019)	2000	Rivers+reservoirs+estuaries	0.15-0.28
					Rivers+streams	0.05
					Reservoirs	0.04-0.07
					Estuaries	0.06-0.16
2000	0.5°×0.5°	-	Static mechanistic LOAC model+	2000	Lake+reservoirs	0.063
					Lakes	0.03

			NEWS2 (Lauerwald et al., 2019)		Reservoirs	0.034
1970s-2010s	-	-	Upscaling (DelSontro et al., 2018)	1970s-2010s	Lakes+reservoirs	0.16-0.27
1970s-2010s	-	-	SPW model (DelSontro et al., 2018)	1970s-2010s	Lakes+reservoirs	0.26-0.38
1960-2015	1 km×1 km	-	Data-driven machine learning model+ upscaling(Marzadri et al., 2021)	1960-2015	Rivers+streams	0.073
1900-2016	0.5°×0.5°	1 year	Mechanistic model: DLEM (Yao et al., 2020)	1900	Rivers+streams	0.07
				2000s	Rivers+streams	0.3
					Rivers	0.04
					Streams	0.24
					Estuaries	0.068
				2007-2016	Rivers+streams	0.3
1980-2016	0.5°×0.5°	1 year	AR6 (Tian et al., 2020; IPCC, 2021)	1980s	Inland+estuaries+coastal zones	0.7 (0.5-0.9)
				1990s	Inland+estuaries+coastal zones	0.7 (0.5-0.9)
				2000s	Inland+estuaries+coastal zones	0.7 (0.5-1.0)
				2007-2016	Inland+estuaries+coastal zones	0.8 (0.5-1.1)
1988-2018	-	1 year	Upscaling+regression (Yan et al., 2021)	1988-2018	Reservoirs	0.04
**1900-2010**	**0.5°×0.5°**	**1 year**	**This study (Mechanistic model: IMAGE-DGNM)**	**1900**	**Inland waters**	**0.38**
					**Streams**	**0.22**
					**Rivers**	**0.12**
					**Lakes**	**0.04**
					**Reservoirs**	**0.001**
					**Estuaries**	**0.02**
				**1970**	**Inland waters**	**0.71**
					**Streams**	**0.35**
					**Rivers**	**0.17**
					**Lakes**	**0.05**
					**Reservoirs**	**0.15**
					**Estuaries**	**0.02**
				**1980**	**Inland waters**	**0.93**
					**Streams**	**0.42**
					**Rivers**	**0.19**
					**Lakes**	**0.06**
					**Reservoirs**	**0.25**
					**Estuaries**	**0.03**
				**1990**	**Inland waters**	**1.12**
					**Streams**	**0.48**
					**Rivers**	**0.22**
					**Lakes**	**0.07**
					**Reservoirs**	**0.35**
					**Estuaries**	**0.03**
				**2000**	**Inland waters**	**1.10**
					**Streams**	**0.45**
					**Rivers**	**0.22**
					**Lakes**	**0.07**
					**Reservoirs**	**0.36**
					**Estuaries**	**0.04**
				**2010**	**Inland waters**	**1.27**
					**Streams**	**0.49**
					**Rivers**	**0.25**
					**Lakes**	**0.08**
					**Reservoirs**	**0.45**
					**Estuaries**	**0.04**

Table S5. Contribution of inland waters to global N_2O emissions in the 1980s, 1990s, 2000s and 2010s

Period	N_2O source to the atmosphere	Data source	N_2O emission ($Tg\ N\ yr^{-1}$)		
			Mean	Min	Max
**2006-2011**	Total	AR5 (IPCC, 2013)	17.9	8.1	30.7
**2007-2016**	Total	AR6 (Tian et al., 2020; IPCC, 2021)	17.0	12.2	23.5
**2006-2011**	Agricultural soil	AR5 (IPCC, 2013)	4.1	1.7	4.8
**2007-2016**	Agricultural soil	AR6 (Tian et al., 2020; IPCC, 2021)	3.8	2.5	5.6
**2006-2011**	Inland, estuarine and coastal waters	AR5 (IPCC, 2013)	0.6	0.1	2.9
**2007-2016**	Inland, estuarine and coastal waters	AR6 (Tian et al., 2020; IPCC, 2021)	0.8	0.5	1.1
**2010s**	**Inland waters**	**This study**	**1.27**	**--**	**--**
**2010s**	**Estuaries and coastal zones**	**(Murray et al., 2015)**	**0.31**	**0.15**	**0.91**
**2010s**	**Inland, estuarine and coastal waters**	**This study and (Murray et al., 2015)**	**1.58**	**1.42**	**2.18**
**2010s**	**Total**	**AR5 (IPCC, 2013) adapted with results of this study**	**18.9**	**9.4**	**30.0**
**2010s**	**Total**	**AR6 (Tian et al., 2020; IPCC, 2021) adapted with results of this study**	**17.8**	**13.1**	**24.6**
**2006-2011**	Total	AR5 (IPCC, 2013)	17.9	8.1	30.7
**2000s**	Total	AR6 (Tian et al., 2020; IPCC, 2021)	16.4	12.3	22.4
**2006-2011**	Agricultural soil	AR5 (IPCC, 2013)	4.1	1.7	4.8
**2000s**	Agricultural soil	AR6 (Tian et al., 2020; IPCC, 2021)	3.4	2.3	5.1
**2006-2011**	Inland, estuarine and coastal waters	AR5 (IPCC, 2013)	0.6	0.1	2.9
**2000s**	Inland, estuarine and coastal waters	AR6 (Tian et al., 2020; IPCC, 2021)	0.7	0.5	1.0
**2000s**	**Inland waters**	**This study**	**1.10**	**--**	**--**
**2000s**	**Estuaries and coastal zones**	**(Murray et al., 2015)**	**0.31**	**0.15**	**0.91**
**2000s**	**Inland, estuarine and coastal waters**	**This study and (Murray et al., 2015)**	**1.41**	**1.25**	**2.01**
**2000s**	**Total**	**AR5 (IPCC, 2013) adapted with results of this study**	**18.7**	**9.2**	**29.8**
**2000s**	**Total**	**AR6 (Tian et al., 2020; IPCC, 2021) adapted with results of this study**	**17.1**	**13.0**	**23.4**
**mid-1990s**	Total	AR5 (IPCC, 2013)	17.5	8.1	30.7
**mid-1990s**	Agricultural soil	AR5 (IPCC, 2013)	3.7	1.7	4.8
**mid-1990s**	Inland, estuarine and coastal waters	AR5 (IPCC, 2013)	0.6	0.1	2.9
**mid-1990s**	**Inland waters**	**This study**	**1.13**	**--**	**--**
**mid-1990s**	**Estuaries and coastal zones**	**(Murray et al., 2015)**	**0.31**	**0.15**	**0.91**
**mid-1990s**	**Inland, estuarine and coastal waters**	**This study and (Murray et al., 2015)**	**1.41**	**1.24**	**2.00**
**mid-1990s**	**Total**	**AR5 (IPCC, 2013) adapted with results of this study**	**18.3**	**9.2**	**29.8**
**1990s**	Total	AR4 (IPCC, 2013)	17.7	8.5	27.7
**1990s**	Total	AR6 (Tian et al., 2020; IPCC, 2021)	15.9	12.2	21.7
**1990s**	Agricultural soil	AR4 (IPCC, 2013)	2.8	1.7	4.8
**1990s**	Agricultural soil	AR6 (Tian et al., 2020; IPCC, 2021)	3.0	2.1	4.8
**1990s**	Inland, estuarine and coastal waters	AR4 (IPCC, 2013)	1.7	0.5	2.9
**1990s**	Inland, estuarine and coastal waters	AR6 (Tian et al., 2020; IPCC, 2021)	0.7	0.5	0.9
**1990s**	**Inland waters**	**This study**	**1.12**	**--**	**--**
**1990s**	**Estuaries and coastal zones**	**(Murray et al., 2015)**	**0.31**	**0.15**	**0.91**
**1990s**	**Inland, estuarine and coastal waters**	**This study and (Murray et al., 2015)**	**1.43**	**1.27**	**2.03**
**1990s**	**Total**	**AR4 (IPCC, 2013) adapted with results of this study**	**17.4**	**9.3**	**26.8**

**1990s**	**Total**	**AR6 (Tian et al., 2020; IPCC, 2021) adapted with results of this study**	**16.6**	**13.0**	**22.8**
**1980s**	Total	AR6 (Tian et al., 2020; IPCC, 2021)	15.5	12.1	20.9
**1980s**	Agricultural soil	AR6 (Tian et al., 2020; IPCC, 2021)	2.6	1.8	4.1
**1980s**	Inland, estuarine and coastal waters	AR6 (Tian et al., 2020; IPCC, 2021)	0.7	0.5	0.9
**1980s**	**Inland waters**	**This study**	**0.93**	**--**	**--**
**1980s**	**Estuaries and coastal zones**	**(Murray et al., 2015)**	**0.31**	**0.15**	**0.91**
**1980s**	**Inland, estuarine and coastal waters**	**This study and (Murray et al., 2015)**	**1.24**	**1.08**	**1.84**
**1980s**	**Total**	**AR6 (Tian et al., 2020; IPCC, 2021) adapted with results of this study**	**16.0**	**12.7**	**21.8**

Reference

Alin, S.R., Rasera, M.d.F.F.L., Salimon, C.I., Richey, J.E., Holtgrieve, G.W., Krusche, A.V., Snidvongs, A., 2011. Physical controls on carbon dioxide transfer velocity and flux in low-gradient river systems and implications for regional carbon budgets. J. Geophys. Res. 116.

Allen, G.H., Pavelsky, T.M., 2018. Global extent of rivers and streams. Science.

Amouroux, D., Roberts, G., Rapsomanikis, S., Andreae, M.O., 2002. Biogenic gas (CH_4, N_2O, DMS) emission to the atmosphere from near-shore and shelf waters of the north-western Black Sea. Estuar. Coast. Shelf Sci. 54, 575-587.

APHA, 1992. Standard methods for the examination of water and wastewater, Vol 4, 18th Edition. American Public Health Association (APHA), American Water Works Association (AWWA) and Water Pollution Control Federation (WPCF), Washington DC.

Audet, J., Wallin, M.B., Kyllmar, K., Andersson, S., Bishop, K., 2017. Nitrous oxide emissions from streams in a Swedish agricultural catchment. Agriculture, Ecosystems & Environment 236, 295-303.

Bange, H.W., Rapsomanikis, S., Andreae, M.O., 1996. Nitrous oxide in coastal waters. Global Biogeochem. Cycles 10, 197-207.

Barthel, M., Bauters, M., Baumgartner, S., Drake, T.W., Bey, N.M., Bush, G., Boeckx, P., Botefa, C.I., Dériaz, N., Ekamba, G.L., Gallarotti, N., Mbayu, F.M., Mugula, J.K., Makelele, I.A., Mbongo, C.E., Mohn, J., Mandea, J.Z., Mpambi, D.M., Ntaboba, L.C., Rukeza, M.B., Spencer, R.G.M., Summerauer, L., Vanlauwe, B., Van Oost, K., Wolf, B., Six, J., 2022. Low N_2O and variable CH_4 fluxes from tropical forest soils of the Congo Basin. Nat. Commun. 13, 330.

Baulch, H.M., Dillon, P.J., Maranger, R., Venkiteswaran, J.J., Wilson, H.F., Schiff, S.L., 2012. Night and day: short-term variation in nitrogen chemistry and nitrous oxide emissions from streams. Freshw. Biol. 57, 509-525.

Baulch, H.M., Schiff, S.L., Maranger, R., Dillon, P.J., 2011. Nitrogen enrichment and the emission of nitrous oxide from streams. Global Biogeochem. Cycles 25.

Beaulieu, J.J., Arango, C.P., Hamilton, S.K., Tank, J.L., 2008. The production and emission of nitrous oxide from headwater streams in the Midwestern United States. Glob. Chang. Biol. 14, 878-894.

Beaulieu, J.J., Shuster, W.D., Rebholz, J.A., 2010. Nitrous oxide emissions from a large, impounded river: The Ohio river. Environ. Sci. Technol. 44, 7527-7533.

Beaulieu, J.J., Smolenski, R.L., Nietch, C.T., Townsend-Small, A., Elovitz, M.S., Schubauer-Berigan, J.P., 2014. Denitrification alternates between a source and sink of nitrous oxide in the hypolimnion of a thermally stratified reservoir. Limnol. Oceanogr. 59, 495-506.

Beaulieu, J.J., Tank, J.L., Hamilton, S.K., Wollheim, W.M., Hall Jr, R.O., Mulholland, P.J., Peterson, B.J., Ashkenas, L.R., Cooper, L.W., Dahm, C.N., Dodds, W.K., Grimm, N.B., Johnson, S.L., McDowell, W.H., Poole, G.C., Maurice Valett, H., Arango, C.P., Bernot, M.J., Burgin, A.J., Crenshaw, C.L., Helton, A.M., Johnson, L.T., O'Brien, J.M., Potter, J.D., Sheibley, R.W., Sobota, D.J., Thomas, S.M., 2011. Nitrous oxide emission from denitrification in stream and river networks. Proc. Nat. Acad. Sci. U.S.A. 108, 214-219.

Beusen, A.H.W., Bouwman, A.F., Van Beek, L.P.H., Mogollón, J.M., Middelburg, J.J., 2016. Global riverine N and P transport to ocean increased during the 20th century despite increased retention along the aquatic continuum. Biogeosciences 13, 2441-2451.

Beusen, A.H.W., Doelman, J.C., Van Beek, L.P.H., Van Puijenbroek, P.J.T.M., Mogollón, J.M., Van Grinsven, H.J.M., Stehfest, E., Van Vuuren, D.P., Bouwman, A.F., 2022. Exploring river nitrogen and phosphorus loading and export to global coastal waters in the Shared Socio-economic pathways. Glob. Environ. Chang. 72, 102426.

Beusen, A.H.W., Van Beek, L.P.H., Bouwman, A.F., Mogollón, J.M., Middelburg, J.J., 2015. Coupling global models for hydrology and nutrient loading to simulate nitrogen and phosphorus retention in surface water. Description of IMAGE-GNM and analysis of performance. Geosci. Model Dev. 8, 4045–4067.

Billen, G., Garnier, J., Grossel, A., Thieu, V., Théry, S., Hénault, C., 2020. Modeling indirect N_2O emissions along the N cascade from cropland soils to rivers. Biogeochemistry 148, 207-221.

Billen, G., Garnier, J., Hanset, P., 1994. Modelling phytoplankton development in whole drainage networks: the RIVERSTRAHLER Model applied to the Seine river system. Hydrobiologia 289, 119-137.

Billen, G., Garnier, J., Silvestre, M., 2015. A simplified algorithm for calculating benthic nutrient fluxes in river systems. Ann. Limnol. 51, 37-47.

Böhlke, J.K., 2002. Groundwater recharge and agricultural contamination. Hydrol. J. 10, 153-179.

Borges, A.V., Darchambeau, F., Lambert, T., Morana, C., Allen, G.H., Tambwe, E., Toengaho Sembaito, A., Mambo, T., Nlandu Wabakhangazi, J., Descy, J.P., Teodoru, C.R., Bouillon, S., 2019. Variations in dissolved greenhouse gases (CO_2, CH_4, N_2O) in the Congo River network overwhelmingly driven by fluvial-wetland connectivity. Biogeosciences 16, 3801-3834.
Borges, A.V., Darchambeau, F., Teodoru, C.R., Marwick, T.R., Tamooh, F., Geeraert, N., Omengo, F.O., Guérin, F., Lambert, T., Morana, C., Okuku, E., Bouillon, S., 2015. Globally significant greenhouse-gas emissions from African inland waters. Nat. Geosci. 8, 637-642.
Bouwman, A.F., Beusen, A.H.W., Griffioen, J., Van Groenigen, J.W., Hefting, M.M., Oenema, O., Van Puijenbroek, P.J.T.M., Seitzinger, S., Slomp, C.P., Stehfest, E., 2013a. Global trends and uncertainties in terrestrial denitrification and N_2O emissions. Philosophical Transactions of the Royal Society B: Biological Sciences 368.
Bouwman, A.F., Beusen, A.H.W., Lassaletta, L., Van Apeldoorn, D.F., Van Grinsven, H.J.M., Zhang, J., van Ittersum, M.K., 2017. Lessons from temporal and spatial patterns in global use of N and P fertilizer on cropland. Sci. Rep. 7, 40366.
Bouwman, A.F., Klein Goldewijk, K., Van der Hoek, K.W., Beusen, A.H.W., Van Vuuren, D.P., Willems, W.J., Rufino, M.C., Stehfest, E., 2013b. Exploring global changes in nitrogen and phosphorus cycles in agriculture induced by livestock production over the 1900-2050 period. Proc. Nat. Acad. Sci. U.S.A. 110, 20882-20887, doi/20810.21073/pnas.1012878108.
Bowden, W.B., Bormann, F.W., 1986. Transport and loss of nitrous oxide in soil water after forest clear cutting. Science 233, 867-869.
Chai, T., Draxler, R.R., 2014. Root mean square error (RMSE) or mean absolute error (MAE)? – Arguments against avoiding RMSE in the literature. Geosci. Model Dev. 7, 1247-1250.
Chen, C.-T.A., Wang, S.-L., Lu, X.-X., Zhang, S.-R., Lui, H.-K., Tseng, H.-C., Wang, B.-J., Huang, H.-I., 2008. Hydrogeochemistry and greenhouse gases of the Pearl River, its estuary and beyond. Quat. Int. 186, 79-90.
Chen, H., Wang, M., Wu, N., Wang, Y., Zhu, D., Gao, Y., Peng, C., 2011. Nitrous oxide fluxes from the littoral zone of a lake on the Qinghai-Tibetan Plateau. Environ. Monit. Assess. 182, 545-553.
Chen, J., Cao, W., Cao, D., Huang, Z., Liang, Y., 2015a. Nitrogen loading and nitrous oxide emissions from a river with multiple hydroelectric reservoirs. Bull. Environ. Contam. Toxicol. 94, 633-639.
Chen, N., Chen, Z., Wu, Y., Hu, A., 2014. Understanding gaseous nitrogen removal through direct measurement of dissolved N_2 and N_2O in a subtropical river-reservoir system. Ecol. Eng. 70, 56-67.
Chen, N., Wu, J., Zhou, X., Chen, Z., Lu, T., 2015b. Riverine N_2O production, emissions and export from a region dominated by agriculture in Southeast Asia (Jiulong River). Agriculture, Ecosystems & Environment 208, 37-47.
Cox, B., 2003. A review of dissolved oxygen modelling techniques for lowland rivers. Sci. Total Environ. 314-316, 303-334.
Davidson, E.A., Firestone, M.K., 1988. Measurement of nitrous oxide dissolved in soil solution. Soil Sci. Soc. Am. J. 52, 1201-1203.
Davidson, E.A., Swank, W.T., 1990. Nitrous oxide dissolved in soil solution: an insignificant pathway of nitrogen loss from a southeastern hardwood forest. Water Resour. Res. 26, 1687-1690.
Davis, M.P., David, M.B., 2018. Nitrous oxide fluxes from agricultural streams in east-central Illinois. Water, Air, Soil Pollut. 229, 354.
de Angelis, M.A., Gordon, L.I., 1985. Upwelling and river runoff as sources of dissolved nitrous oxide to the Alsea estuary, Oregon. Estuar. Coast. Shelf Sci. 20, 375-386.
De Bie, M.J.M., Middelburg, J.J., Starink, M., Laanbroek, H.J., 2002. Factors controlling nitrous oxide at the microbial community and estuarine scale. Mar. Ecol. Prog. Ser. 240, 1-9.
De Wilde, H.P.J., De Bie, M.J.M., 2000. Nitrous oxide in the Schelde estuary: production by nitrification and emission to the atmosphere. Mar. Chem. 69, 203-216.
Deemer, B.R., Harrison, J.A., Li, S., Beaulieu, J.J., Delsontro, T., Barros, N., Bezerra-Neto, J.F., Powers, S.M., Dos Santos, M.A., Vonk, J.A., 2016. Greenhouse gas emissions from reservoir water surfaces: A new global synthesis. Bioscience 66, 949-964.
DelSontro, T., Beaulieu, J.J., Downing, J.A., 2018. Greenhouse gas emissions from lakes and impoundments: Upscaling in the face of global change. Limnology and Oceanography Letters 3, 64-75.
DeSimone, J., Macrae, M.L., Bourbonniere, R.A., 2010. Spatial variability in surface N_2O fluxes across a riparian zone and relationships with soil environmental conditions and nutrient supply. Agriculture, Ecosystems & Environment 138, 1-9.

Deurer, M., von der Heide, C., Bottcher, J., Duijnisveld, W.H.M., Weymann, D., Well, R., 2008. The dynamics of N_2O near the groundwater table and the transfer of N_2O into the unsaturated zone: A case study from a sandy aquifer in Germany. CATENA 72, 362-373.
Dong, L., Nedwell, D., B., Underwood , G., J. C., Thornton, D.C.O., Rusmana, I., 2002. Nitrous oxide formation in the Colne Estuary, England: the central role of nitrite. Appl. Environ. Microbiol. 68, 1240-1249.
Dong, L., Nedwell, D.B., Colbeck, I., Finch, J., 2004. Nitrous oxide emission from some English and Welsh Rivers and estuaries. Water, Air, and Soil Pollution: Focus 4, 127-134.
Dowdell, R.J., Burford, J.R., Crees, R., 1979. Losses of nitrous oxide dissolved in drainage water from agricultural land. Nature 278, 342-343.
FAO 2021. FAOSTAT database collections. Accessed on April 12, 2022. Rome. Food and Agriculture Organization of the United Nations. http://www.fao.org/faostat/en/#data.
Ferrón, S., Ortega, T., Gómez-Parra, A., Forja, J.M., 2007. Seasonal study of dissolved CH_4, CO_2 and N_2O in a shallow tidal system of the bay of Cádiz (SW Spain). J. Mar. Sys. 66, 244-257.
Fox, R.J., Fisher, T.R., Gustafson, A.B., Jordan, T.E., Kana, T.M., Lang, M.W., 2014. Searching for the missing nitrogen: biogenic nitrogen gases in groundwater and streams. The Journal of Agricultural Science 152, 96-106.
Gardner, J.R., Fisher, T.R., Jordan, T.E., Knee, K.L., 2016. Balancing watershed nitrogen budgets: accounting for biogenic gases in streams. Biogeochemistry 127, 231-253.
Garnier, J., Billen, G., Cébron, A., 2007. Modelling nitrogen transformations in the lower Seine River and estuary (France): Impact of wastewater release on oxygenation and N_2O emission. Hydrobiologia 588, 291-302.
Garnier, J., Billen, G., Palfner, L., 2000. Understanding the oxygen budget and related ecological processes in the river Mosel: the RIVERSTRAHLER approach. Hydrobiologia 410, 151-166.
Garnier, J., Cébron, A., Tallec, G., Billen, G., Sebilo, M., Martinez, A., 2006. Nitrogen behaviour and nitrous oxide emission in the tidal Seine River estuary (France) as influenced by human activities in the upstream watershed. Biogeochemistry 77, 305-326.
Groffman, P.M., Gold, A.J., Addy, K., 2000. Nitrous oxide production in riparian zones and its importance to national emission inventories. Chemosphere - Global Change Science 2, 291-299.
Groffman, P.M., Gold, A.J., Jacinthe, P.A., 1998. Nitrous oxide production in riparian zones and groundwater. Nutri. Cycl. Agroecosyst. 52, 179-186.
Guérin, F., Abril, G., Tremblay, A., Delmas, R., 2008. Nitrous oxide emissions from tropical hydroelectric reservoirs. Geophys. Res. Lett. 35.
Hahn, J., Junge, C., 1977. Atmospheric nitrous oxide: A critical review. Zeitschrift für Naturforschung A 32, 190-214.
Hama-Aziz, Z.Q., Hiscock, K.M., Cooper, R.J., 2017. Indirect nitrous oxide emission factors for agricultural field drains and headwater streams. Environ. Sci. Technol. 51, 301-307.
Harrison, J., Matson, P., 2003. Patterns and controls of nitrous oxide emissions from waters draining a subtropical agricultural valley. Global Biogeochem. Cycles 17.
Hartmann, J., Lauerwald, R., Moosdorf, N., 2019. GLORICH - Global river chemistry database. PANGAEA.
He, Y., Wang, X., Chen, H., Yuan, X., Wu, N., Zhang, Y., Yue, J., Zhang, Q., Diao, Y., Zhou, L., 2017. Effect of watershed urbanization on N_2O emissions from the Chongqing metropolitan river network, China. Atmos. Environ. 171, 70-81.
Helder, W., de Vries, R.T.P., 1983. Estuarine nitrite maxima and nitrifying bacteria (Ems-Dollard estuary). Neth. J. Sea Res. 17, 1-18.
Hemond, H.F., Duran, A.P., 1989. Fluxes of N_2O at the sediment-water and water-atmosphere boundaries of a nitrogen-rich river. Water Resour. Res. 25, 839-846.
Hendzel, L.L., Matthews, C.J.D., Venkiteswaran, J.J., St. Louis, V.L., Burton, D., Joyce, E.M., Bodaly, R.A., 2005. Nitrous oxide fluxes in three experimental boreal forest reservoirs. Environ. Sci. Technol. 39, 4353-4360.
Hinshaw, S.E., Dahlgren, R.A., 2013. Dissolved nitrous oxide concentrations and fluxes from the eutrophic San Joaquin River, California. Environ. Sci. Technol. 47, 1313-1322.
Hiscock, K.M., Bateman, A.S., Muhlherr, I.H., Fukada, T., Dennis, P.F., 2003. Indirect emissions of nitrous oxide from regional aquifers in the United Kingdom. Environ. Sci. Technol. 37, 3507-3512.
Hlaváčová, E., Rulík, M., Čáp, L., Mach, V., 2006. Greenhouse gas (CO_2, CH_4, N_2O) emissions to the atmosphere from a small lowland stream in Czech Republic. Archiv Für Hydrobiologie, 165(3), 339–353. doi:10.1127/0003-9136/2006/0165-0339 Archiv Für Hydrobiologie 165, 339-353.

Hu, M., Chen, D., Dahlgren, R.A., 2016. Modeling nitrous oxide emission from rivers: a global assessment. Glob. Chang. Biol. 22, 3566-3582.

Huttunen, J., Hammar, T., Manninen, P., Servomaa, K., Martikainen, P., Manninen, T., Servomaa, P., Martikainen, K., 2004. Potential springtime greenhouse gas emissions from a small southern boreal lake (Keihäsjärvi, Finland). Boreal Environ. Res. 9.

Huttunen, J.T., Alm, J., Liikanen, A., Juutinen, S., Larmola, T., Hammar, T., Silvola, J., Martikainen, P.J., 2003. Fluxes of methane, carbon dioxide and nitrous oxide in boreal lakes and potential anthropogenic effects on the aquatic greenhouse gas emissions. Chemosphere 52, 609-621.

Huttunen, J.T., Väisänen, T.S., Heikkinen, M., Hellsten, S., Nykänen, H., Nenonen, O., Martikainen, P.J., 2002a. Exchange of CO_2, CH_4 and N_2O between the atmosphere and two northern boreal ponds with catchments dominated by peatlands or forests. Plant Soil 242, 137-146.

Huttunen, J.T., Väisänen, T.S., Hellsten, S.K., Heikkinen, M., Nykänen, H., Jungner, H., Niskanen, A., Virtanen, M.O., Lindqvist, O.V., Nenonen, O.S., Martikainen, P.J., 2002b. Fluxes of CH_4, CO_2, and N_2O in hydroelectric reservoirs Lokka and Porttipahta in the northern boreal zone in Finland. Global Biogeochem. Cycles 16, 3-1-3-17.

IPCC 1996. Revised 1996 Guidelines for National Greenhouse Gas Inventories National Greenhouse Gas Inventory Program (NGGIP), Houghton, J.T., Meira Filho, L.G., Lim, B., Treanton, K., Mamaty, I., Bonduki, Y., Griggs, D.J., Callender, B.A., eds. (Intergovernmental panel on Climate Change / Organization for Economic Cooperation and Development).

IPCC, 2007. Climate change 2007. The physical science basis. Contribution of Working Group I to the Fourth Assessment Report of the Intergovernmental Panel on Climate Change (edited by Solomon, S., Chin, D., Manning, M., Marquis, M., Averyt, K., Tignor, M.M.B., Le Roy Miller jr., H., Chen, Z.). Cambridge University Press, New York, 996 pp. p.

IPCC 2013. Climate Change 2013: The Physical Science Basis. Contribution of Working Group I to the Fifth Assessment Report of the Intergovernmental Panel on Climate Change (Cambridge, United Kingdom and New York, NY, USA).

IPCC 2021. Climate Change 2021: The Physical Science Basis. Contribution of Working Group I to the Sixth Assessment Report of the Intergovernmental Panel on Climate Change, Masson-Delmotte, V., Zhai, P., Pirani, A., Connors, S.L., Péan, C., Berger, S., Caud, N., Chen, Y., Goldfarb, L., Gomis, M.I., Huang, M., Leitzell, K., Lonnoy, E., Matthews, J.B.R., Maycock, T.K., Waterfield, T., Yelekçi, O., Yu, R., Zhou, B., eds.

Jahangir, M.M., Johnston, P., Barrett, M., Khalil, M.I., Groffman, P.M., Boeckx, P., Fenton, O., Murphy, J., Richards, K.G., 2013. Denitrification and indirect N_2O emissions in groundwater: hydrologic and biogeochemical influences. J. Contam. Hydrol. 152, 70-81.

Jahangir, M.M.R., Johnston, P., Khalil, M.I., Hennessy, D., Humphreys, J., Fenton, O., Richards, K.G., 2012. Groundwater: A pathway for terrestrial C and N losses and indirect greenhouse gas emissions. Agriculture, Ecosystems & Environment 159, 40-48.

Jauhiainen, J., Silvennoinen, H., 2012. Diffusion GHG fluxes at tropical peatland drainage canal water surfaces. Suo 63, 93-105.

Jurado, A., Borges, A.V., Brouyère, S., 2017. Dynamics and emissions of N_2O in groundwater: A review. Sci. Total Environ. 584-585, 207-218.

Kaplan, W.A., Elkins, J.W., Kolb, C.E., McElroy, M.B., Wofsy, S.C., Duran, A.P., 1978. Nitrous oxide in fresh water systems: an estimate for the yield of atmospheric N_2O associated with disposal of human waste. Pagoph 116, 423-438.

Keuskamp, J.A., Van Drecht, G., Bouwman, A.F., 2012. European-scale modelling of groundwater denitrification and associated N_2O production. Environ. Pollut. 165, 67-76.

Kortelainen, P., Larmola, T., Rantakari, M., Juutinen, S., Alm, J., Martikainen, P.J., 2020. Lakes as nitrous oxide sources in the boreal landscape. Glob. Chang. Biol. 26, 1432-1445.

Kroeze, C., Dumont, E., Seitzinger, S., 2010. Future trends in emissions of N_2O from rivers and estuaries. Journal of Integrative Environmental Sciences 7, 71-78.

Kroeze, C., Dumont, E., Seitzinger, S.P., 2005. New estimates of global emissions of N_2O from rivers and estuaries. Environmental Sciences 2, 159-165.

Kroeze, C., Mosier, A., Bouwman, L., 1999. Closing the global N_2O budget: A retrospective analysis 1500-1994. Global Biogeochem. Cycles 13, 1-8.

LaMontagne, M.G., Duran, R., Valiela, I., 2003. Nitrous oxide sources and sinks in coastal aquifers and coupled estuarine receiving waters. Sci. Total Environ. 309, 139-149.

Lauerwald, R., Regnier, P., Figueiredo, V., Enrich-Prast, A., Bastviken, D., Lehner, B., Maavara, T., Raymond, P., 2019. Natural lakes are a minor global source of N_2O to the atmosphere. Global Biogeochem. Cycles 33, 1564-1581.
Laursen, A.E., Seitzinger, S.P., 2004. Diurnal patterns of denitrification, oxygen consumption and nitrous oxide production in rivers measured at the whole-reach scale. Freshw. Biol. 49, 1448-1458.
Law, C.S., Rees, A.P., Owens, N.J.P., 1992. Nitrous oxide: Estuarine sources and atmospheric flux. Estuar. Coast. Shelf Sci. 35, 301-314.
Lehner, B., Döll, P., 2004. Development and validation of a global database of lakes, reservoirs and wetlands. J. Hydrol. 296, 1-22.
Lehner, B., Reidy Liermann, C., Revenga, C., Vörösmarty, C., Fekete, B., Crouzet, P., Döll, P., Endejan, M., Frenken, K., Magome, J., Nilsson, C., Robertson, J.C., Rödel, R., Sindorf, N., Wisser, D., 2011. High-resolution mapping of the world's reservoirs and dams for sustainable river-flow management. Front. Ecol. Environ. 9, 494-502.
Lehner, B., Verdin, K., Jarvis, A., 2008. New global hydrography derived from spaceborne elevation data. Eos 89, 93-94.
Lemon, E., Lemon, D., 1981. Nitrous oxide in fresh waters of the Great Lakes Basin. Limnol. Oceanogr. 26, 867-879.
Li, P., 2010. Methane and nitrous oxide in the Yellow River Estuary, the Yellow Sea and the Bohai Sea. Ocean University of China, Qingdao.
Lima, I.B.T., Victoria, R.L., Novo, E.M.L.M., Feigl, B.J., Ballester, M.V.R., Ometto, J.P., 2002. Methane, carbon dioxide and nitrous oxide emissions from two Amazonian Reservoirs during high water table. SIL Proceedings, 1922-2010 28, 438-442.
Liu, X., Liu, C., Li, S., Wang, F., Wang, B., Wang, Z., 2011. Spatiotemporal variations of nitrous oxide (N_2O) emissions from two reservoirs in SW China. Atmos. Environ. 45, 5458-5468.
Maavara, T., Lauerwald, R., Laruelle, G.G., Akbarzadeh, Z., Bouskill, N.J., Van Cappellen, P., Regnier, P., 2019. Nitrous oxide emissions from inland waters: Are IPCC estimates too high? Glob. Chang. Biol. 25, 473-488.
Mach, V., Bednařík, A., Čáp, L., Šipoš, J., Rulík, M., 2016. Seasonal Measurement of greenhouse gas concentrations and emissions along the longitudinal profile of a small stream. Pol. J. Environ. Stud. 25, 2047-2056.
Mander, Ü., Well, R., Weymann, D., Soosaar, K., Maddison, M., Kanal, A., Lõhmus, K., Truu, J., Augustin, J., Tournebize, J., 2014. Isotopologue ratios of N_2O and N_2 measurements underpin the importance of denitrification in differently N-loaded riparian alder forests. Environ. Sci. Technol. 48, 11910-11918.
Marty, D., Bonin, P., Michotey, V., Bianchi, M., 2001. Bacterial biogas production in coastal systems affected by freshwater inputs. Cont. Shelf Res. 21, 2105-2115.
Marzadri, A., Amatulli, G., Tonina, D., Bellin, A., Shen, L.Q., Allen, G.H., Raymond, P.A., 2021. Global riverine nitrous oxide emissions: The role of small streams and large rivers. Sci. Total Environ. 776, 145148.
McAleer, E.B., Coxon, C.E., Richards, K.G., Jahangir, M.M.R., Grant, J., Mellander, P.E., 2017. Groundwater nitrate reduction versus dissolved gas production: A tale of two catchments. Sci. Total Environ. 586, 372-389.
McElroy, M.B., Elkins, J.W., Wofsy, S.C., Kolb, C.E., Duran, A.R., kaplan, W.A., 1978. Production and release of N_2O from the Potomac Estuarie. Limnol. Oceanogr. 23, 1168-1182.
McMahon, P.B., Dennehy, K.F., 1999. N_2O emissions from a nitrogen-enriched river. Environ. Sci. Technol. 33, 21-25.
Mengis, M., Gächter, R., Wehrli, B., 1996. Nitrous oxide emissions to the atmosphere from an artificially oxygenated lake. Limnol. Oceanogr. 41, 548-553.
Ministry_of_Water_Resources_of_the_People's_Republic_of_China, 2003. China River Sediment Bulletin 2002.
Mookherji, S., McCarty, G.W., Angier, J.T., 2003. Dissolved gas analysis for assessing the fate of nitrate in wetlands. JAWRA Journal of the American Water Resources Association 39, 381-387.
Mosier, A., Kroeze, C., Nevison, C., Oenema, O., Seitzinger, S., van Cleemput, O., 1998. Closing the global N_2O budget: nitrous oxide emissions through the agricultural nitrogen cycle. Nutri. Cycl. Agroecosyst. 52, 225-248.
Muhlherr, I.H., Hiscock, K.M., 1998. Nitrous oxide production and consumption in British limestone aquifers. J. Hydrol. 211, 126-139.

Murray, R.H., Erler, D.V., Eyre, B.D., 2015. Nitrous oxide fluxes in estuarine environments: response to global change. Glob. Chang. Biol. 21, 3219-3245.

Musenze, R.S., Grinham, A., Werner, U., Gale, D., Sturm, K., Udy, J., Yuan, Z., 2014. Assessing the spatial and temporal variability of diffusive methane and nitrous oxide emissions from subtropical freshwater reservoirs. Environ. Sci. Technol. 48, 14499-14507.

Nirmal Rajkumar, A., Barnes, J., Ramesh, R., Purvaja, R., Upstill-Goddard, R.C., 2008. Methane and nitrous oxide fluxes in the polluted Adyar River and estuary, SE India. Mar. Pollut. Bull. 56, 2043-2051.

Outram, F.N., Hiscock, K.M., 2012. Indirect nitrous oxide emissions from surface water bodies in a lowland arable catchment: a significant contribution to agricultural greenhouse gas budgets? Environ. Sci. Technol. 46, 8156-8163.

Paludan, C., Blicher-Mathiesen, G., 1996. Losses of inorganic carbon and nitrous oxide from a temperate freshwater wetland in relation to nitrate loading. Biogeochemistry 35, 305-326.

Raymond, P.A., Hartmann, J., Lauerwald, R., Sobek, S., McDonald, C., Hoover, M., Butman, D., Striegl, R., Mayorga, E., Humborg, C., Kortelainen, P., Dürr, H., Meybeck, M., Ciais, P., Guth, P., 2013. Global carbon dioxide emissions from inland waters. Nature 503, 355-359.

Richey, J.E., Devol, A.H., Wofsy, S.C., Victoria, R., Riberio, M.N.G., 1988. Biogenic gases and the oxidation and reduction of carbon in Amazon River and floodplain waters. Limnol. Oceanogr. 33, 551-561.

Rijkswaterstaat, 2022. Historische waterkwantiteit-en waterkwaliteitsgegevens. Retrieved from https://www.rijkswaterstaat.nl/water/waterdata-en-waterberichtgeving/waterdata. Last accessed January 10, 2022.

Robinson, A.D., Nedwell, D.B., Harrison, R.M., Ogilvie, B.G., 1998. Hypernutrified estuaries as sources of N_2O emission to the atmosphere:the estuary of the River Colne, Essex, UK. Mar. Ecol. Prog. Ser. 164, 59-71.

Ronen, D., Magaritz, M., Almon, E., 1988. Contaminated aquifers are a forgotten component of the global N_2O budget. Nature 335, 57-59.

Rosamond, M.S., Thuss, S.J., Schiff, S.L., 2012. Dependence of riverine nitrous oxide emissions on dissolved oxygen levels. Nat. Geosci. 5, 715-718.

Schade, J.D., Bailio, J., McDowell, W.H., 2016. Greenhouse gas flux from headwater streams in New Hampshire, USA: Patterns and drivers. Limnol. Oceanogr. 61, S165-S174.

Seitzinger, S.P., Kroeze, C., 1998. Global distribution of nitrous oxide production and N inputs in freshwater and coastal marine ecosystems. Global Biogeochem. Cycles 12, 93-113.

Seitzinger, S.P., Kroeze, C., Styles, R.V., 2000. Global distribution of N_2O emissions from aquatic systems: natural emissions and anthropogenic effects. Chemosphere - Global Change Science 2, 267-279.

Silvennoinen, H., Liikanen, A., Rintala, J., Martikainen, P.J., 2008. Greenhouse gas fluxes from the eutrophic Temmesjoki River and its Estuary in the Liminganlahti Bay (the Baltic Sea). Biogeochemistry 90, 193-208.

Smith, R.L., Böhlke, J.K., 2019. Methane and nitrous oxide temporal and spatial variability in two midwestern USA streams containing high nitrate concentrations. Sci. Total Environ. 685, 574-588.

Soued, C., del Giorgio, P.A., Maranger, R., 2016. Nitrous oxide sinks and emissions in boreal aquatic networks in Québec. Nat. Geosci. 9, 116-120.

Spalding, R.F., Parrott, J.D., 1994. Shallow groundwater denitrification. Sci. Total Environ. 141, 17-25.

Stehfest, E., Van Vuuren, D.P., Kram, T., Bouwman, A.F., 2014. Integrated Assessment of Global Environmental Change with IMAGE 3.0. Model description and policy applications. PBL Netherlands Environmental Assessment Agency, The Hague.

Sturm, K., Yuan, Z., Gibbes, B., Grinham, A., 2014. Methane and nitrous oxide sources and emissions in a subtropical freshwater reservoir, south east Queensland, Australia. Biogeosciences 11, 5245–5258.

Sutanudjaja, E.H., Van Beek, R., Wanders, N., Wada, Y., Bosmans, J.H.C., Drost, N., Van Der Ent, R.J., De Graaf, I.E.M., Hoch, J.M., De Jong, K., Karssenberg, D., López López, P., Peßenteiner, S., Schmitz, O., Straatsma, M.W., Vannametee, E., Wisser, D., Bierkens, M.F.P., 2018. PCR-GLOBWB 2: A 5 arcmin global hydrological and water resources model. Geosci. Model Dev. 11, 2429-2453.

Syakila, A., Kroeze, C., 2011. The global nitrous oxide budget revisited. Greenhouse Gas Measurement and Management 1, 17-26.

Thomann, R.V., Mueller, J.A., 1987. Principles of water quality modeling and control. Harper Collins Publishers, New York.

Tian, H., Xu, R., Canadell, J.G., Thompson, R.L., Winiwarter, W., Suntharalingam, P., Davidson, E.A., Ciais, P., Jackson, R.B., Janssens-Maenhout, G., Prather, M.J., Regnier, P., Pan, N., Pan, S., Peters, G.P.,

Shi, H., Tubiello, F.N., Zaehle, S., Zhou, F., Arneth, A., Battaglia, G., Berthet, S., Bopp, L., Bouwman, A.F., Buitenhuis, E.T., Chang, J., Chipperfield, M.P., Dangal, S.R.S., Dlugokencky, E., Elkins, J.W., Eyre, B.D., Fu, B., Hall, B., Ito, A., Joos, F., Krummel, P.B., Landolfi, A., Laruelle, G.G., Lauerwald, R., Li, W., Lienert, S., Maavara, T., MacLeod, M., Millet, D.B., Olin, S., Patra, P.K., Prinn, R.G., Raymond, P.A., Ruiz, D.J., van der Werf, G.R., Vuichard, N., Wang, J., Weiss, R.F., Wells, K.C., Wilson, C., Yang, J., Yao, Y., 2020. A comprehensive quantification of global nitrous oxide sources and sinks. Nature 586, 248-256.

Upstill-Goddard, R.C., Salter, M.E., Mann, P.J., Barnes, J., Poulsen, J., Dinga, B., Fiske, G.J., Holmes, R.M., 2017. The riverine source of CH_4 and N_2O from the Republic of Congo, western Congo Basin. Biogeosciences 14, 2267-2281.

USGS 2022. United States Geological Survey: Water Quality Samples for the Nation. (United States Geological Survey).

Van Beek, L.P.H., Wada, Y., Bierkens, M.F.P., 2011. Global monthly water stress: 1. Water balance and water availability. Water Resour. Res. 47, W07517.

Van Drecht, G., Bouwman, A.F., Knoop, J.M., Beusen, A.H.W., Meinardi, C.R., 2003. Global modeling of the fate of nitrogen from point and nonpoint sources in soils, groundwater and surface water. Global Biogeochem. Cycles 17, 1115.

van Puijenbroek, P., Beusen, A.H.W., Bouwman, A.F., 2019. Global nitrogen and phosphorus in urban waste water based on the Shared Socio-economic pathways. J. Environ. Manag. 231, 446-456.

Vilmin, L., Mogollón, J.M., Beusen, A.H.W., Bouwman, A.F., 2018. Forms and subannual variability of nitrogen and phosphorus loading to global river networks over the 20th century. Glob. Planet. Chang. 163, 67-85.

Vilmin, L., Mogollón, J.M., Beusen, A.H.W., van Hoek, W.J., Liu, X., Middelburg, J.J., Bouwman, A.F., 2020. Modeling process‐based biogeochemical dynamics in surface fresh waters of large watersheds with the IMAGE‐DGNM framework. Journal of Advances in Modeling Earth Systems 12, 1-19.

von der Heide, C., Böttcher, J., Deurer, M., Weymann, D., Well, R., Duijnisveld, W.H.M., 2008. Spatial variability of N_2O concentrations and of denitrification-related factors in the surficial groundwater of a catchment in Northern Germany. J. Hydrol. 360, 230-241.

Wang, F., Yu, Q., Yan, W., Tian, S., Zhang, P., Wang, J., 2022. Basin-scale control on N_2O loss rate and emission in the Changjiang River network, China. Frontiers in Marine Science 9.

Wang, H., Wang, W., Yin, C., Wang, Y., Lu, J., 2006. Littoral zones as the “hotspots” of nitrous oxide (N_2O) emission in a hyper-eutrophic lake in China. Atmos. Environ. 40, 5522-5527.

Wang, H., Zhang, L., Yao, X., Xue, B., Yan, W., 2017a. Dissolved nitrous oxide and emission relating to denitrification across the Poyang Lake aquatic continuum. Journal of Environmental Sciences 52, 130-140.

Wang, L., Ye, W., Song, D., Hou, J., Zheng, W., Zhang, G., 2016. Seasonal variation of dissolved nitrous oxide concentration and flux in the downstream Yellow River and their regulating factors. Acta Scientiae Circumstantiae 36, 1917-1927 (in Chinese with English abstract).

Wang, R., Zhang, H., Zhang, W., Zheng, X., Butterbach-Bahl, K., Li, S., Han, S., 2020. An urban polluted river as a significant hotspot for water–atmosphere exchange of CH_4 and N_2O. Environ. Pollut. 264, 114770.

Wang, S., Liu, C., Yeager, K.M., Wan, G., Li, J., Tao, F., Lü˘, Y., Liu, F., Fan, C., 2009. The spatial distribution and emission of nitrous oxide (N_2O) in a large eutrophic lake in eastern China: Anthropogenic effects. Sci. Total Environ. 407, 3330-3337.

Wang, X., He, Y., Yuan, X., Chen, H., Peng, C., Yue, J., Zhang, Q., Diao, Y., Liu, S., 2017b. Greenhouse gases concentrations and fluxes from subtropical small reservoirs in relation with watershed urbanization. Atmos. Environ. 154, 225-235.

Wanninkhof, R., 1992. Relationship between wind speed and gas exchange over the ocean. J. Geophys. Res. 97, 7373-7382.

Weiss, R.F., Price, B.A., 1980. Nitrous oxide solubility in water and seawater. Mar. Chem. 8, 347-359.

Well, R., Augustin, J., Davis, J., Griffith, S.M., Meyer, K., Myrold, D.D., 2001. Production and transport of denitrification gases in shallow groundwater. Nutri. Cycl. Agroecosyst. 60, 65-75.

Well, R., Augustin, J., Meyer, K., Myrold, D.D., 2003. Comparison of field and laboratory measurement of denitrification and N_2O production in the saturated zone of hydromorphic soils. Soil Biol. Biochem. 35, 783-799.

Well, R., Eschenbach, W., Flessa, H., von der Heide, C., Weymann, D., 2012. Are dual isotope and isotopomer ratios of N_2O useful indicators for N_2O turnover during denitrification in nitrate-contaminated aquifers? Geochim. Cosmochim. Acta 90, 265-282.
Well, R., Flessa, H., Jaradat, F., Toyoda, S., Yoshida, N., 2005a. Measurement of isotopomer signatures of N_2O in groundwater. J. Geophys. Res. Biogeosci. 110, n/a-n/a.
Well, R., Weymann, D., Flessa, H., 2005b. Recent research progress on the significance of aquatic systems for indirect agricultural N_2O emissions. Environmental Sciences 2, 143-151.
Weller, D.E., Correll, D.L., Jordan, T.E., 1994. Denitrification in riparian forests receiving agricultural discharges. Global Wetlands: Old World and New, 117-131.
Weymann, D., Geistlinger, H., Well, R., von der Heide, C., Flessa, H., 2010. Kinetics of N_2O production and reduction in a nitrate-contaminated aquifer inferred from laboratory incubation experiments. Biogeosciences 7, 1953-1972.
Weymann, D., Well, R., Flessa, H., von der Heide, C., Deurer, M., Meyer, K., Konrad, C., Walther, W., 2008. Groundwater N_2O emission factors of nitrate-contaminated aquifers as derived from denitrification progress and N_2O accumulation. Biogeosciences 5, 1215-1226.
Wilcock, R.J., Sorrell, B.K., 2008. Emissions of greenhouse gases CH_4 and N_2O from low-gradient streams in agriculturally developed catchments. Water, Air, Soil Pollut. 188, 155-170.
WMO 2021. World Meteorological Organization (WMO) Greenhouse Gas Bulletin No.17: The State of Greenhouse Gases in the Atmosphere Based on Global Observations through 2020.
Wong, W.W., Grace, M.R., Cartwright, I., Cardenas, M.B., Zamora, P.B., Cook, P.L.M., 2013. Dynamics of groundwater-derived nitrate and nitrous oxide in a tidal estuary from radon mass balance modeling. Limnol. Oceanogr. 58, 1689-1706.
Xiao, Q., Hu, Z., Fu, C., Bian, H., Lee, X., Chen, S., Shang, D., 2019a. Surface nitrous oxide concentrations and fluxes from water bodies of the agricultural watershed in Eastern China. Environ. Pollut. 251, 185-192.
Xiao, Q., Xu, X., Zhang, M., Duan, H., Hu, Z., Wang, W., Xiao, W., Lee, X., 2019b. Coregulation of nitrous oxide emissions by nitrogen and temperature in China's third largest freshwater lake (Lake Taihu). Limnol. Oceanogr. 64, 1070-1086.
Xu, J., Wang, Y., Yin, J., Wang, Q., Zhang, F., He, L., Sun, C., 2005. Transformation of dissolved inorganic nitrogen species and nitrification and denitrification processes in the near sea section of Zhujiang River. Acta Scientiae Circumstantiae 25, 686-692 (in Chinese with English abstract).
Yan, F., Sillanpää, M., Kang, S., Aho, K.S., Qu, B., Wei, D., Li, X., Li, C., Raymond, P.A., 2018. Lakes on the Tibetan Plateau as conduits of greenhouse gases to the atmosphere. J. Geophys. Res. Biogeosci. 123, 2091-2103.
Yan, W., Laursen, A., Wang, F., Sun, P., Seitzinger, S., 2004. Measurement of Denitrification in the Changjiang River. Environmental Chemistry - ENVIRON CHEM 1, 95-98.
Yan, X., Thieu, V., Garnier, J., 2021. Long-term evolution of greenhouse gas emissions from global reservoirs. Frontiers in Environmental Science 9, 705477.
Yang, L., Li, H., Wang, J., 2019. Spatial and temporal variability of nitrous oxide emissions from a large subtropical reservoir in Eastern China. Pol. J. Environ. Stud. 28, 3497-3503.
Yao, Y., Tian, H., Shi, H., Pan, S., Xu, R., Pan, N., Canadell, J.G., 2020. Increased global nitrous oxide emissions from streams and rivers in the Anthropocene. Nat. Clim. Chang. 10, 138-142.
Zhang, G., Zhang, J., Ren, J., Li, J., Liu, S., 2008. Distributions and sea-to-air fluxes of methane and nitrous oxide in the North East China Sea in summer. Mar. Chem. 110, 42-55.
Zhang, G., Zhang, J., Xu, J., Zhang, F., 2006. Distributions, sources and atmospheric fluxes of nitrous oxide in Jiaozhou Bay. Estuar. Coast. Shelf Sci. 68, 557-566.
Zhang, G.L., Zhang, J., Liu, S.M., Ren, J.L., Zhao, Y.C., 2010. Nitrous oxide in the Changjiang (Yangtze River) Estuary and its adjacent marine area: Riverine input, sediment release and atmospheric fluxes. Biogeosciences 7, 3505-3516.
Zhao, B., Zhang, Q., 2021. N_2O emission and its influencing factors in subtropical streams, China. Ecological Processes 10, 54.
Zhao, J., Zhang, G., Wu, Y., Zhang, J., 2009a. Distribution and emission of nitrous oxide from the Changjiang River. Huanjing Kexue Xuebao/Acta Scientiae Circumstantiae 29, 1995-2002.
Zhao, J., Zhang, G., Wu, Y., Zhang, Y., 2009b. Distribution and emission of nitrous oxide from the Changjiang River. Acta Scientiae Circumstantiae 29, 1995-2002 (in Chinese with English abstract).

Zhou, W., Ma, Y., Well, R., Wang, H., Yan, X., 2018. Denitrification in shallow groundwater below different arable land systems in a high nitrogen-loading region. J. Geophys. Res. Biogeosci. 123, 991-1004.

Zhou, Y., Xu, X., Han, R., Li, L., Feng, Y., Yeerken, S., Song, K., Wang, Q., 2019. Suspended particles potentially enhance nitrous oxide (N2O) emissions in the oxic estuarine waters of eutrophic lakes: Field and experimental evidence. Environ. Pollut. 252, 1225-1234.

Zhu, D., Wu, Y., Wu, N., Chen, H., He, Y., Zhang, Y., Peng, C., Zhu, Q.a., 2015. Nitrous oxide emission from infralittoral zone and pelagic zone in a shallow lake: Implications for whole lake flux estimation and lake restoration. Ecol. Eng. 82, 368-375.
